# Supplementary material for: Decatungstate-catalyzed radical disulfuration through direct C-H functionalization for the preparation of unsymmetrical disulfides
Source: Nat Commun. 2022 Jul 6;13:3886. doi: 10.1038/s41467-022-31617-5 (PMC9259577; doi:10.1038/s41467-022-31617-5)
Supplement: Supplementary file 1 — Supplementary Information [file 41467_2022_31617_MOESM1_ESM.pdf]

Decatungstate-Catalyzed Radical Disulfuration Through Direct C-H Functionalization for the Preparation of Unsymmetrical Disulfides

Jingjing Zhang, Armido Studer*

Institute of Organic Chemistry, University of Münster, Corrensstrasse 40, 48149 Münster, Germany

Contents

1. Supplementary Methods 2

1.1 General information 2

1.2 Synthesis of tetrasulfides, SS-(tert-butyl) 4-methylbenzenesulfono(dithioperoxoate) and substrates.... 4

1.3 Optimization of conditions with cyclohexane as the substrate and **3a** as the disulfuration reagent 5

1.4 General synthetic procedure for the synthesis of disulfides **4** and **6** 6

1.5 Gram-scale experiments 8

1.6 Mechanism studies 9

1.6.1 Mechanism study for trapping radical intermediates by TEMPO 9

1.6.2 Mechanism study for trapping radical intermediates by methyl acrylate 11

1.6.3 KIE experiments 11

1.6.4 Stern-Volmer quenching experiments 11

1.7 Characterization data 14

2. Supplementary Figures 32

3. Supplementary References 87

1. Supplementary Methods

1.1 General information

Chemicals: All reactions involving air- or moisture-sensitive reagents or intermediates were carried out in pre-heated glassware using standard Schlenk techniques at ambient room temperature, unless otherwise specified. All commercially available reagents were purchased from Sigma-Aldrich, Alfa Aesar, TCI Chemicals, Acros Organics or ABCR in the highest purity grade and used without further purification. Acetonitrile (99.8%, extra dry, AcroSeal) was used as received from Acros Organics. Thin layer chromatography (TLC) was performed on Merck silica gel 60 F-254 plates and visualized by fluorescence quenching under UV light. Column chromatography was performed on Merck or Fluka silica gel 60 (40-63 μm). Reversed phase medium pressure liquid chromatography (RP-MPLC) was carried out on an automatic flash-system by Reveleris®IES and on a Büchi C-850 FlashPrep device with commercially available 4 g Reveleris®-C18-flash cartridges as the stationary phase. The detection was performed by UV-absorption (λ = 210, 230, 254, 320 nm).

NMR Spectroscopy: ^{1}H-NMR, ^{13}C-NMR and ^{19}F-NMR spectra were recorded using a Bruker DPX 300, Bruker AV 300, Bruker AV 400, Agilent DD2 500 or Agilent DD2 600 spectrometer at 299 K or 300 K. ^{1}H-NMR chemical shifts are given relative to TMS and residual monoprotic solvent peaks were used as an internal reference for ^{1}H-NMR ($CDCl_3$: δ = 7.26 ppm) and ^{13}C-NMR spectra ($CDCl_3$: δ = 77.16 ppm). ^{19}F-NMR spectra are referenced according to the proton signal as the primary reference for the unified chemical shift scale. Coupling constants (*J*) are quoted to the nearest 0.1 Hz. The following abbreviations (or combinations thereof) were used to describe ^{1}H-, ^{13}C and ^{19}F-NMR multiplicities: s = singlet, broad singlet = brs, d = doublet, t = triplet, q = quartet, hept = heptet, m = multiplet.

Mass Spectrometry: High-resolution (HRMS) ESI (m/z) spectra were measured on a Bruker MicroTof or ThermoFisher Scientific LTQ XL Orbitrap. High- resolution APCI (m/z) spectra were measured on a Thermo-Fisher Scientific Orbitrap LTQ XL. High-resolution EI (m/z) spectra were measured on a Thermo-Fischer Scientific Exactive GC Orbitrap GC-MS System. MassLinx 4.0 of Water-Micromass was used for data analysis.

GC-FID: GC-FID was conducted on an Agilent GC 6890 equipped with a flame ionization detector (FID) and an Agilent HP-5, Methyl Siloxan (Model No: 19091Z-413) column using H_2 as carrier gas with a flow rate of 1.5 mL min^{-1}. The method used starts with the injection temperature T_0, the column is heated to temperature T_1 (ramp) and this temperature is held for an additional time *t* (T_0 = 50 °C, T_1 = 300 °C, ramp = 10 °C min^{-1}, *t* = 15 min).

Light Sources: Photochemical reactions were performed with one Kessil PR160L (40 W, 390 nm) as light sources. The reaction temperature was measured to be 60 ± 2 °C using this setup through fixing the distance between the lamp and the reaction tube to 3 cm.

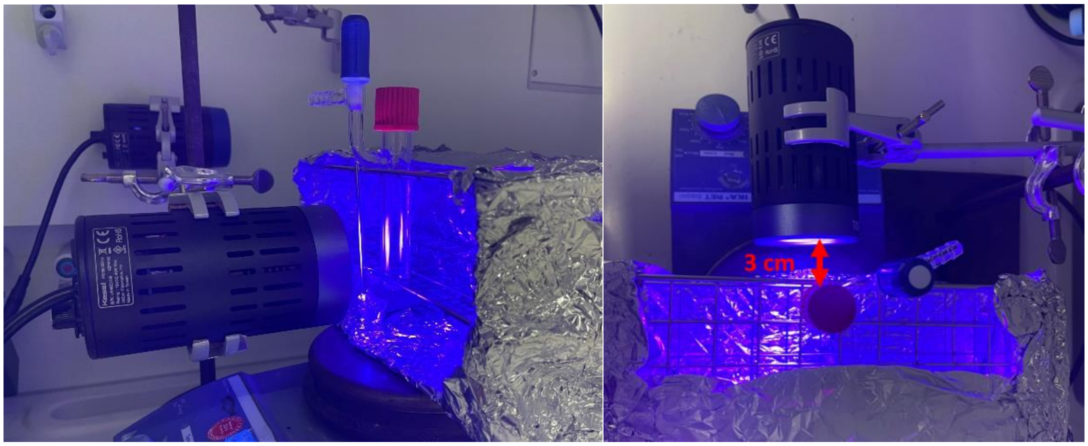

Supplementary Figure 1. Photoreactors used in this work.

1.2 Synthesis of tetrasulfides, SS-(tert-butyl) 4-methylbenzenesulfono(dithioperoxoate) and substrates.

Tetrasulfides **3a**[1], **3b**[2], **3d**[3], **3h**[1], **3i**[4], **3j**[5], **3k**[6], **3l**[1] and SS-(tert-butyl) 4-methylbenzenesulfono(dithioperoxoate)[7] were synthesized through the reported methods. Substrate **5m**[8] and **5n**[9] were synthesized through the reported methods.

Tetrasulfides were synthesized by the following general method:

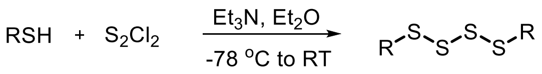

Thiol (10 mmol) and Et_3N (10 mmol) were added dropwise as a solution in dry ether (30 mL) to a solution of freshly distilled S_2Cl_2 (5 mmol) in dry ether (30 mL) cooled to -78°C in a dry ice/acetone bath. After the addition is complete, the solution was stirred at - 78°C for an additional 30 minutes, after which it was diluted with ether (100 mL) and washed with water, Na_2CO_3 (sat.) and brine. The organic layer was dried over $MgSO_4$, filtered and concentrated in vacuo. The crude oil was purified by column chromatography using 5% EtOAc in hexane to obtain the corresponding tetrasulfides.

The SS-(tert-butyl) 4-methylbenzenesulfono(dithioperoxoate) was synthesized by the following method:

To a solution of *ᵗ*BuSS*ᵗ*Bu (10 mmol) in Et_2O (40 mL) was added SO_2Cl_2 (10 mmol) slowly at 0 °C and then the mixture was stirred at the same temperature for 1 hour. Then a solution of TsSK (20 mmol) in acetone (50 mL) was added slowly at 0 °C and continue stirred at room temperature for 2 hours. The precipitate was filtered and evaporated under reduced pressure and purified by column chromatography.

5m was synthesized by the following method:

To a solution of dehydrocholic acid (2.5 mmol, 1.0 equiv.), 4-dimethylaminopyridine (0.13 mmol, 5.0 mol%) and 4-hydroxybenzaldehyde (2.5 mmol, 1.0 equiv.) in CH_2Cl_2 (10 mL) was added 1-ethyl- (3-(3-dimethylamino)propyl)carbodiimide hydrochloride (2.8 mmol, 1.1 equiv.). The reaction mixture was stirred at 0 °C for 1 hour and then overnight at rt. The reaction was quenched with H_2O and extracted with CH_2Cl_2. The combined organics were dried over $MgSO_4$, concentrated in vacuo and purified by chromatography on silica gel (pentane/EtOAc = 1/1) to afford the title compound.

5n was synthesized by the following method:

To a stirred solution of commercially available indomethacin (1 mmol, 1 equiv.) in DCM (5 mL) were added p-hydroxybenzaldehyde (1.2 mmol, 1.2 equiv.) and DCC (1.5 mmol, 1.5 equiv.). The resulting mixture was stirred at room temperature for 16 h. Then, the crude reaction mixture was filtered over a pad of Celite eluting with EtOAc. This yellow solution was concentrated by rotary evaporation and the residue was purified by column chromatography using hexane: DCM mixtures as eluent to afford the desired compound.

1.3 Optimization of conditions with cyclohexane as the substrate and 3a as the disulfuration reagent

Supplementary Table 1. Optimization of reaction conditions.[a]

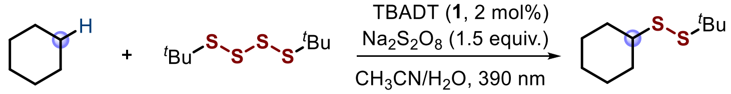

2a, 3 mmol | **3a**, 0.3 mmol | **4a**

Entry	Deviation from standard conditions	Yield[b]
1	standard conditions	86%
2	without TBADT	trace
3	CH_2Cl_2/H_2O as solvent	trace[c]
4	CH_3CN as solvent	18%
5	25 °C	46%
6	without $Na_2S_2O_8$	12%
7	without $Na_2S_2O_8$, CH_3CN/(HCl aq.) as solvent	28%[d]
8	$Na_2S_2O_4$ in replace of $Na_2S_2O_8$	trace
9	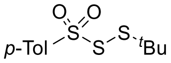 as disulfuration reagent	trace[e]

[a]Reaction conditions: tetrasulfide **3a** (0.3 mmol, 1.0 equiv.), cyclohexane **2a** (3 mmol, 10 equiv.), photocatalyst TBADT 2 mol%, $Na_2S_2O_8$ (0.45 mmol, 1.5 equiv.), solvent 3 mL (CH_3CN/H_2O, *v*/*v*, 2/1), 390 nm, Ar, 60 °C, and 12 h. [b]Isolated yield. [c]Solvent 3 mL (CH_2Cl_2/H_2O, *v*/*v*, 2/1). [d]Solvent 3 mL [CH_3CN/(HCl aq. 1.0 M), *v*/*v*, 2/1]. [e]Without $Na_2S_2O_8$.

Reaction conditions: To an oven dried Schlenk tube with a magnetic stirring bar, the tetrasulfide **3a** (0.3 mmol, 1.0 equiv.), the cyclohexane **2a** (3 mmol, 10 mmol), photocatalyst TBADT (2 mol%), $Na_2S_2O_8$ (0.45 mmol, 1.5 equiv.) and 3.0 mL mixed solvent (CH_3CN/H_2O, *v*/*v*, 2/1) were added under argon atmosphere using standard Schlenk techniques at ambient temperature. After backfilling with nitrogen, the tube was placed in a photoreactor, stirred and irradiated with a Kessil 40 W 390 nm lamp for 12 h at 60 °C. The solvent was removed under reduced pressure and the crude residue was purified by column chromatography with hexane as the eluent to afford the desired product.

1.4 General synthetic procedure for the synthesis of disulfides 4 and 6

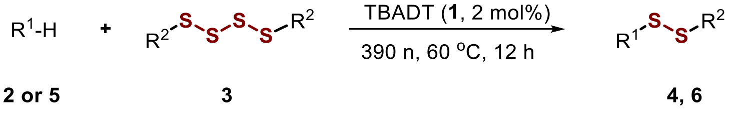

General procedure A: To an oven dried Schlenk tube with a magnetic stirring bar, the tetrasulfides **3** (0.3 mmol, 1.0 equiv.), the substrates (3 mmol, 10 equiv.), $Na_2S_2O_8$ (0.45 mmol, 1.5 equiv.), photocatalyst TBADT (2 mol%), and 3.0 mL mixed solvent (CH_3CN/H_2O, *v*/*v*, 2/1) were added under argon atmosphere using standard Schlenk techniques at ambient temperature. After backfilling with nitrogen, the tube was placed in a photoreactor, stirred and irradiated with a Kessil 40 W 390 nm lamp for 12 h at 60 ^oC. The solvent was removed under reduced pressure and the crude residue was purified by column chromatography with hexane as the eluent to afford the desired product.

General procedure B: To an oven dried Schlenk tube with a magnetic stirring bar, the tetrasulfide **3** (0.3 mmol, 1.0 equiv.), the substrate (3 mmol, 10 equiv.), photocatalyst TBADT (2 mol%) and 3.0 mL mixed solvent [CH_3CN/(HCl aq., 1.0 M), *v*/*v*, 2/1] were added under argon atmosphere using standard Schlenk techniques at ambient temperature. After backfilling with nitrogen, the tube was placed in a photoreactor, stirred and irradiated with a Kessil 40 W 390 nm lamp for 12 h at 60 ^oC. The solvent was removed under reduced pressure and the crude residue was purified by column chromatography with hexane as the eluent to afford the desired product.

General procedure C: To an oven dried Schlenk tube with a magnetic stirring bar, the substrate (0.3 mmol, 1.0 equiv.), the tetrasulfide **3** (0.45 mmol, 1.5 equiv.), photocatalyst TBADT (2 mol%) and 3.0 mL mixed solvent [CH_3CN/(HCl aq., 1.0 M), *v*/*v*, 2/1] were added under argon atmosphere using standard Schlenk techniques at ambient temperature. After backfilling with nitrogen, the tube was placed in a photoreactor, stirred and irradiated with a Kessil 40 W 390 nm lamp for 12 h at 60 ^oC. The solvent was removed under reduced pressure and the crude residue was purified by column chromatography with hexane as the eluent to afford the desired product.

General procedure D: To an oven dried Schlenk tube with a magnetic stirring bar, the tetrasulfide **3** (0.3 mmol, 1.0 equiv.), the substrate (1.5 mmol, 5.0 equiv.), $Na_2S_2O_8$ (0.45 mmol, 1.5 equiv.), photocatalyst TBADT (2 mol%) and 3.0 mL mixed solvent (CH_3CN/H_2O, *v*/*v*, 2/1) were added under argon atmosphere using standard Schlenk techniques at ambient temperature. After backfilling with nitrogen, the tube was placed in a photoreactor, stirred and irradiated with a Kessil 40 W 390 nm lamp for 12 h at 60 ^oC. The solvent was removed under reduced pressure and the crude residue was purified by column chromatography with hexane as the eluent to afford the desired product.

General procedure E: To an oven dried Schlenk tube with a magnetic stirring bar, the substrate (0.3 mmol, 1.0 equiv.), the tetrasulfide **3** (0.45 mmol, 1.5 equiv.), $Na_2S_2O_8$ (0.45 mmol, 1.5 equiv.), photocatalyst TBADT (2 mol%) and 3.0 mL mixed solvent (CH_3CN/H_2O, *v*/*v*, 2/1) were added under argon atmosphere using standard

Schlenk techniques at ambient temperature. After backfilling with nitrogen, the tube was placed in a photoreactor, stirred and irradiated with a Kessil 40 W 390 nm lamp for 12 h at 60 ^{o}C. The solvent was removed under reduced pressure and the crude residue was purified by column chromatography with hexane as the eluent to afford the desired product.

1.5 Gram-scale experiments

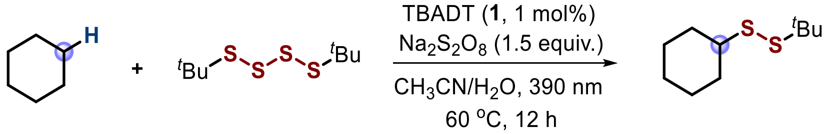

2a, 50 mmol **3a**, 5.0 mmol **4a**, 84%, 1.72 g

Reaction conditions: To an oven dried Schlenk tube with a magnetic stirring bar, the tetrasulfide **3a** (5.0 mmol, 1.0 equiv.), the substrate **2a** (50 mmol, 10 equiv.), $Na_2S_2O_8$ (7.5 mmol, 1.5 equiv.), photocatalyst TBADT (0.05 mmol, 1 mol%) and 30 mL mixed solvent (CH_3CN/H_2O, *v*/*v*, 2/1) were added under argon atmosphere using standard Schlenk techniques at ambient temperature. After backfilling with nitrogen, the tube was placed in a photoreactor, stirred and irradiated with a Kessil 40 W 390 nm lamp for 12 h at 60 ^oC. The solvent was removed under reduced pressure and the crude residue was purified by column chromatography with hexane as the eluent to afford the desired product **4a** (8.4 mmol, 1.72 g, 84% yield).

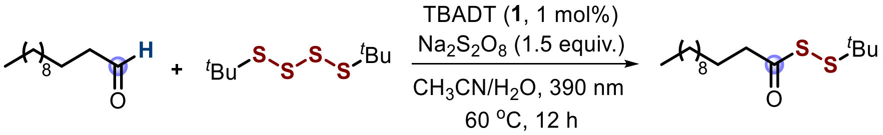

5g, 10 mmol **3a**, 15 mmol **6g**, 88%, 2.67 g

Reaction conditions: To an oven dried Schlenk tube with a magnetic stirring bar, substrate **5g** (10 mmol, 1.0 equiv.), the tetrasulfide **3a** (15 mmol, 1.5 equiv.), $Na_2S_2O_8$ (15 mmol, 1.5 equiv.), photocatalyst TBADT (0.1 mmol, 1 mol%) and 30 mL mixed solvent (CH_3CN/H_2O, *v*/*v*, 2/1) were added under argon atmosphere using standard Schlenk techniques at ambient temperature. After backfilling with nitrogen, the tube was placed in a photoreactor, stirred and irradiated with a Kessil 40 W 390 nm lamp for 12 h at 60 ^oC. The solvent was removed under reduced pressure and the crude residue was purified by column chromatography with EtOAc and hexane (hexane/EtOAc = 10/1, v/v)) as the eluent to afford the desired product **6g** (8.8 mmol, 2.67 g, 88% yield).

1.6 Mechanism studies

1.6.1 Mechanism study for trapping radical intermediates by TEMPO

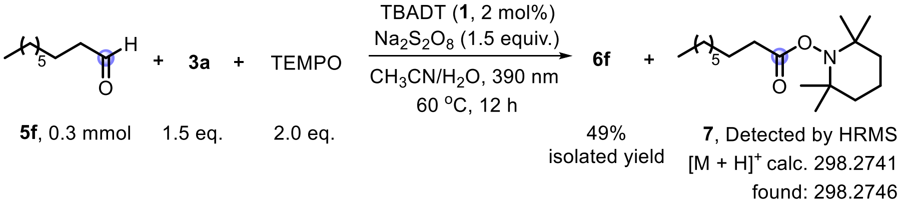

Reaction conditions: To an oven dried Schlenk tube with a magnetic stirring bar, the substrate **5f** (0.3 mmol, 1.0 equiv.), the tetrasulfide **3a** (0.45 mmol, 1.5 equiv.), $Na_2S_2O_8$ (0.45 mmol, 1.5 equiv.), TEMPO (0.6 mmol, 2.0 eruiv.), photocatalyst TBADT (2 mol%) and 3.0 mL mixed solvent (CH_3CN/H_2O, *v*/*v*, 2/1) were added under argon atmosphere using standard Schlenk techniques at ambient temperature. After backfilling with nitrogen, the tube was placed in a photoreactor, stirred and irradiated with a Kessil 40 W 390 nm lamp for 12 h at 60 °C. The resulting mixture was analyzed by HRMS (ESI), and the TEMPO-trapping product **7** was detected. Then, the solvent was removed under reduced pressure and the crude residue was purified by column chromatography with hexane as the eluent to afford the desired product **6f** (0.147 mmol, 38.5 mg, 49% yield).

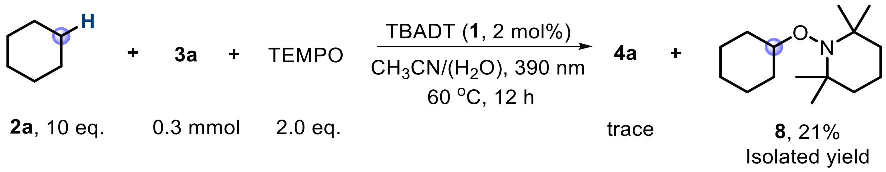

Reaction conditions: To an oven dried Schlenk tube with a magnetic stirring bar, the tetrasulfide **3a** (0.3 mmol, 1.0 equiv.), the substrate **2a** (3 mmol, 10 equiv.), TEMPO (0.6 mmol, 2.0 eruiv.), photocatalyst TBADT (2 mol%) and 3.0 mL mixed solvent (CH_3CN/H_2O, *v*/*v*, 2/1) were added under argon atmosphere using standard Schlenk techniques at ambient temperature. After backfilling with nitrogen, the tube was placed in a photoreactor, stirred and irradiated with a Kessil 40 W 390 nm lamp for 12 h at 60 °C. Then the solvent was removed under reduced pressure and the crude residue was purified by column chromatography with hexane as the eluent to afford the adduct **8** (0.130 mmol, 31 mg, 21% yield).

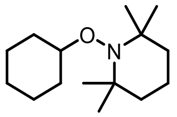

8

1-(Cyclohexyloxy)-2,2,6,6-tetramethylpiperidine (**8**):

^{1}H NMR (300 MHz, $CDCl_3$) δ 3.57 (td, *J* = 9.7, 4.5 Hz, 1H), 2.11 – 1.94 (m, 2H), 1.81 – 1.68 (m, 2H), 1.44 (d, *J* = 5.9 Hz, 6H), 1.27 – 0.99 (m, 18H). **^{13}C NMR** (76 MHz, $CDCl_3$) δ 81.8, 59.6, 40.3, 32.9, 26.0, 25.1, 17.3. The NMR spectra were in agreement with those reported in the literature.[10]

1.6.2 Mechanism study for trapping radical intermediates by methyl acrylate

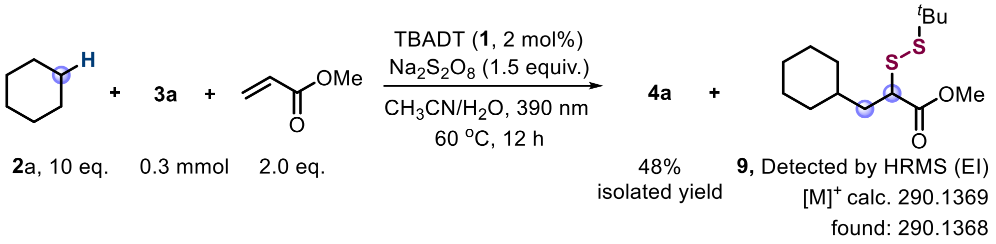

Reaction conditions: To an oven dried Schlenk tube with a magnetic stirring bar, the tetrasulfide **3a** (0.3 mmol, 1.0 equiv.), the substrate **2a** (3 mmol, 10 equiv.), $Na_2S_2O_8$ (0.45 mmol, 1.5 equiv.), methyl acrylate (0.6 mmol, 2.0 equiv.), photocatalyst TBADT (2 mol%) and 3.0 mL mixed solvent (CH_3CN/H_2O, *v*/*v*, 2/1) were added under argon atmosphere using standard Schlenk techniques at ambient temperature. After backfilling with nitrogen, the tube was placed in a photoreactor, stirred and irradiated with a Kessil 40 W 390 nm lamp for 12 h at 60 °C. The resulting mixture was analyzed by HRMS (EI), and the product **9**, generated by the radical cascade reaction could be detected. Then, the solvent was removed under reduced pressure and the crude residue was purified by column chromatography with hexane as the eluent to afford the desired product **4a** (0.288 mmol, 58.7 mg, 48% yield).

1.6.3 KIE experiments

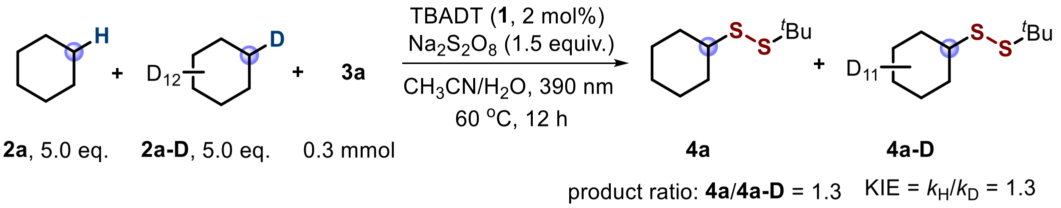

Reaction conditions: To an oven dried Schlenk tube with a magnetic stirring bar, the tetrasulfide **3a** (0.3 mmol, 1.0 equiv.), the substrate **2a** (1.5 mmol, 5.0 equiv.), fully deuterated substrate **2a-D** (1.5 mmol, 5.0 equiv.), $Na_2S_2O_8$ (0.45 mmol, 1.5 equiv.), photocatalyst TBADT (2 mol%) and 3.0 mL mixed solvent (CH_3CN/H_2O *v*/*v*, 2/1) were added under argon atmosphere using standard Schlenk techniques at ambient temperature. After backfilling with nitrogen, the tube was placed in a photoreactor, stirred and irradiated with a Kessil 40 W 390 nm lamp for 12 h at 60 °C. Then the resulting mixture was analyzed by GC. The ratio of disulfuration products **4a** and **4a-D** was 1.3, indicating that the KIE value is 1.3 for the HAT process.

1.6.4 Stern-Volmer quenching experiments

Stern-Volmer luminescence quenching analysis was conducted using a Jasco FP8300 spectrofluorometer at 25 °C. The following parameters were employed: Excitation bandwidth = 5 nm, data interval = 0.2 nm, scan speed = 500 nm/min, response time = 0.2 sec. The samples were measured in Hellma fluorescence QS quartz

cuvettes (chamber volume = 1.4 mL, H × W × D = 46 mm × 12.5 mm, 12.5 mm) fitted with a PTFE stopper. Samples were prepared in 1.4 mL quartz cuvettes equipped with PTFE stoppers inside an argon filled glove bag. The acetonitrile solution of TBADT (7.6×10^{-4} M) were excited at λ_{ex} = 324 nm and the emission was recorded at 380 nm. For each quenching experiment, 10 µl of the quenching reagent were titrated to a solution (1.0 mL) of TBADT in a screw-top 10.0 mm quartz cuvette I_0 is the luminescence intensity without the quencher, I is the intensity in the presence of the quencher. The results are listed below:

Entry	[**3a**]	I_o/I
1	0	1.00
2	0.001033	1.11
3	0.002066	1.26
4	0.003099	1.41
5	0.004133	1.55
6	0.005165	1.74

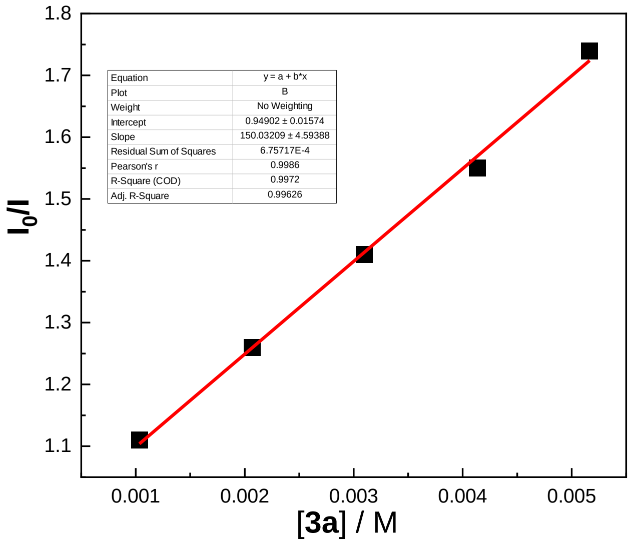

Comment: Stern-Volmer quenching experiments showed that the tetrasulfide **3a** could indeed also be oxidized with the photoexcited TBADT. As shown above, the Stern-Volmer constant is around 1.5×10^2 M^{-1}, and we could calculate the quenching rate constant k_q (~3×10^9 $M^{-1}s^{-1}$) based on the lifetime of the emissive excited state of TBADT (4.75×10^{-8} s).[11] Moreover, referring to the literature,[12] the reaction of alkanes with the photoexcited TBADT has a comparable quenching rate constant (1×10^8 $M^{-1}s^{-1}$). Although it seems reasonable that the tetrasulfide can also quench the excited photocatalyst, we are not able to draw a possible pathway how we can convert the tetrasulfide radical cation to the disulfuration product. We definitely need a CH abstraction to generate the C-radical and the following trapping with the tetrasulfide is established and fast. Moreover, for

the less activated substrates such as cyclohexane the substrate is used in excess further supporting the C-H abstraction path. For the activated substrates the H-abstraction will be faster. We therefore assume, that even if reductive quenching will happen, it is likely not a productive pathway. Moreover, we have shown the TEMPO-trapping product is formed.

1.7 Characterization data

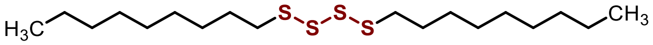

3c

1,4-Dinonyltetrasulfane (**3c**):

^{1}H NMR (400 MHz, $CDCl_3$) δ 3.01 – 2.94 (m, 4H), 1.78 (p, *J* = 7.3 Hz, 4H), 1.43 (dq, *J* = 12.7, 6.1 Hz, 4H), 1.38 – 1.22 (m, 20H), 0.97 – 0.85 (m, 6H). **^{13}C NMR** (101 MHz, $CDCl_3$) δ 39.5, 31.9, 29.5, 29.3, 29.2, 29.1, 28.5, 22.7, 14.1.

HRMS (EI) m/z: $[M]^+$ Calcd for $C_{18}H_{38}S_4$ 382.1856; found 382.1856.

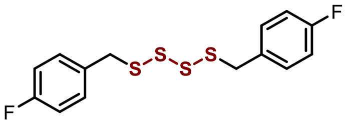

3e

1,4-Bis(4-fluorobenzyl)tetrasulfane (**3e**):

^{1}H NMR (400 MHz, $CDCl_3$) δ 7.31 (ddd, *J* = 8.9, 5.5, 2.8 Hz, 4H), 7.10 – 7.00 (m, 4H), 4.13 (s, 4H). **^{13}C NMR** (101 MHz, $CDCl_3$) δ 162.4 (d, *J* = 246.7 Hz), 132.0 (d, *J* = 3.3 Hz), 131.2 (d, *J* = 8.2 Hz), 115.6 (d, *J* = 21.6 Hz), 42.7. **^{19}F NMR** (376 MHz, $CDCl_3$) δ -114.14.

The NMR spectra were in agreement with those reported in the literature.[13]

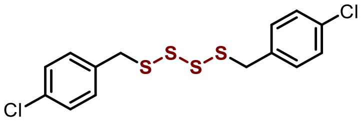

3f

1,4-Bis(4-chlorobenzyl)tetrasulfane (**3f**):

^{1}H NMR (400 MHz, $CDCl_3$) δ 7.34 – 7.29 (m, 4H), 7.28 – 7.22 (m, 4H), 4.09 (s, 4H). **^{13}C NMR** (101 MHz, $CDCl_3$) δ 134.8, 133.7, 130.8, 128.9, 42.7.

HRMS (EI) m/z: $[M]^+$ Calcd for $C_{14}H_{12}Cl_2S_4$ 377.9193; found 377.9193.

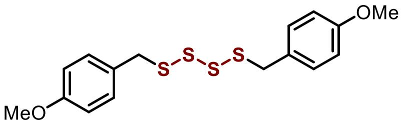

3g

1,4-Bis(4-methoxybenzyl)tetrasulfane (**3g**):

^{1}H NMR (400 MHz, $CDCl_3$) δ 7.31 – 7.25 (m, 5H), 6.93 – 6.86 (m, 5H), 4.16 (s, 4H), 3.83 (s, 6H). **^{13}C NMR** (101 MHz, $CDCl_3$) δ 159.2, 130.7, 128.2, 114.1, 55.3, 43.2.

The NMR spectra were in agreement with those reported in the literature.[14]

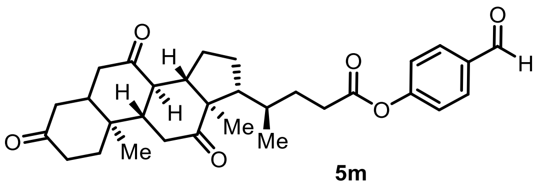

5m

4-Formylphenyl (4R)-4-((8R,9S,10S,13R,14S,17R)-10,13-dimethyl-3,7,12-trioxohexadecahydro-1H-cyclopenta[a]phenanthren-17-yl)pentanoate (**5m**):

^{1}H NMR (300 MHz, $CDCl_3$) δ 9.98 (s, 1H), 7.98 – 7.84 (m, 2H), 7.30 – 7.19 (m, 2H), 2.97 – 2.79 (m, 3H), 2.73 – 2.48 (m, 2H), 2.41 – 1.83 (m, 15H), 1.66 – 1.50 (m, 2H), 1.40 (s, 3H), 1.28 (dt, *J* = 9.9, 5.0 Hz, 1H), 1.09 (s, 3H), 0.92 (d, *J* = 6.6 Hz, 3H).

The NMR spectra were in agreement with those reported in the literature.[8]

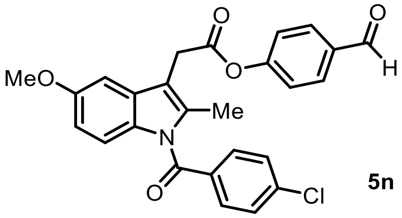

5n

4-Formylphenyl 2-(1-(4-chlorobenzoyl)-5-methoxy-2-methyl-1H-indol-3-yl)acetate (**5n**):

^{1}H NMR (300 MHz, $CDCl_3$) δ 9.97 (s, 1H), 7.90 (d, *J* = 8.6 Hz, 2H), 7.68 (d, *J* = 8.5 Hz, 2H), 7.47 (d, *J* = 8.5 Hz, 2H), 7.25 (d, *J* = 8.5 Hz, 2H), 7.04 (d, *J* = 2.5 Hz, 1H), 6.88 (d, *J* = 9.0 Hz, 1H), 6.70 (dd, *J* = 9.1, 2.5 Hz, 1H), 3.94 (s, 2H), 3.83 (s, 3H), 2.47 (s, 3H).

The NMR spectra were in agreement with those reported in the literature.[9]

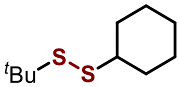

4a

1-(Tert-butyl)-2-cyclohexyldisulfane (**4a**):

Following the General Procedure A and purification via column chromatography on silica gel (pentane), **4a** was obtained as a colorless oil (105.2 mg, 86% yield).

When using alkane **2a** as the limiting reagent (**3a** 0.45 mmol, **2a** 0.3 mmol), **4a** was obtained in 29% GC yield with CH_2Br_2 as the internal standard.

^{1}H NMR (300 MHz, $CDCl_3$) δ 2.75 – 2.60 (m, 1H), 2.13 – 2.00 (m, 2H), 1.78 (p, *J* = 4.0 Hz, 2H), 1.65 – 1.55 (m, 1H), 1.31 (s, 9H), 1.29 – 1.17 (m, 5H). **^{13}C NMR** (76 MHz, $CDCl_3$) δ 50.4, 47.3, 33.0, 30.0, 26.1, 25.8.

The NMR spectra were in agreement with those reported in the literature.[1]

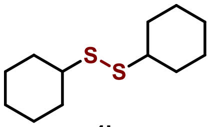

4b

1,2-Dicyclohexyldisulfane (**4b**):

Following the General Procedure A and purification via column chromatography on silica gel (pentane), **4b** was obtained as a colorless oil (122.8 mg, 89% yield).

When using alkane **2a** as the limiting reagent (**3b** 0.45 mmol, **2a** 0.3 mmol), **4b** was obtained in 25% GC yield with CH_2Br_2 as the internal standard.

^{1}H NMR (300 MHz, $CDCl_3$) δ 2.68 (tt, *J* = 10.6, 3.8 Hz, 2H), 2.04 (dt, *J* = 9.2, 4.9 Hz, 4H), 1.86 – 1.71 (m, 4H), 1.67 – 1.57 (m, 2H), 1.29 (td, *J* = 11.1, 5.7 Hz, 10H). **^{13}C NMR** (76 MHz, $CDCl_3$) δ 50.0, 32.9, 26.1, 25.7.

The NMR spectra were in agreement with those reported in the literature.[15]

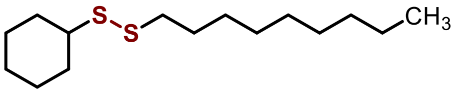

4c

1-Cyclohexyl-2-nonyldisulfane (**4c**):

Following the General Procedure A and purification via column chromatography on silica gel (pentane), **4c** was obtained as a colorless oil (133.1 mg, 81% yield).

When using alkane **2a** as the limiting reagent (**3c** 0.45 mmol, **2a** 0.3 mmol), **4c** was obtained in 28% GC yield with CH_2Br_2 as the internal standard.

^{1}H NMR (300 MHz, $CDCl_3$) δ 2.78 – 2.62 (m, 3H), 2.03 (ddt, *J* = 9.8, 3.6, 1.9 Hz, 2H), 1.78 (qt, *J* = 4.6, 2.2 Hz, 2H), 1.65 (qd, *J* = 7.9, 3.9 Hz, 3H), 1.43 – 1.12 (m, 17H), 0.94 – 0.80 (m, 3H). **^{13}C NMR** (76 MHz, $CDCl_3$) δ 49.5, 40.3, 32.9, 31.9, 29.5, 29.28, 29.25, 28.6, 26.1, 25.7, 22.7, 14.1.

HRMS (EI) m/z: $[M\ ]^+$ Calcd for $C_{15}H_{30}S_2$ 274.1783; found 274.1783.

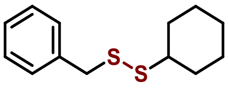

4d

1-Benzyl-2-cyclohexyldisulfane (**4d**):

Following the General Procedure A and purification via column chromatography on silica gel (pentane/EtOAc = 20/1, v/v), **4d** was obtained as a colorless oil (112.8 mg, 79% yield).

When using alkane **2a** as the limiting reagent (**3d** 0.45 mmol, **2a** 0.3 mmol), **4d** was obtained in 20% GC yield with CH_2Br_2 as the internal standard.

^{1}**H NMR** (300 MHz, $CDCl_3$) δ 7.42 – 7.26 (m, 5H), 3.89 (s, 2H), 2.44 – 2.28 (m, 1H), 1.95 (dt, *J* = 13.0, 3.6 Hz, 2H), 1.83 – 1.67 (m, 2H), 1.57 (d, *J* = 5.2 Hz, 1H), 1.37 – 1.10 (m, 5H). ^{13}**C NMR** (76 MHz, $CDCl_3$) δ 137.7, 129.3, 128.5, 127.4, 49.3, 44.7, 32.8, 26.1, 25.6.

The NMR spectra were in agreement with those reported in the literature.[1]

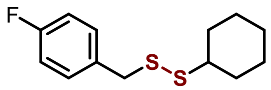

4e

1-Cyclohexyl-2-(4-fluorobenzyl)disulfane (**4e**):

Following the General Procedure A and purification via column chromatography on silica gel (pentane/EtOAc = 20/1, v/v), **4e** was obtained as a colorless oil (116.7 mg, 76% yield).

^{1}**H NMR** (300 MHz, $CDCl_3$) δ 7.28 – 7.16 (m, 2H), 6.99 – 6.87 (m, 2H), 3.78 (s, 2H), 2.33 (td, *J* = 10.6, 3.7 Hz, 1H), 1.93 – 1.81 (m, 2H), 1.74 – 1.62 (m, 2H), 1.55 – 1.49 (m, 1H), 1.28 – 1.06 (m, 5H). ^{19}**F NMR** (282 MHz, $CDCl_3$) δ -115.09. ^{13}**C NMR** (76 MHz, $CDCl_3$) δ 162.2 (d, *J* = 246.1 Hz), 133.5 (d, *J* = 3.2 Hz), 130.9 (d, *J* = 8.1 Hz), 115.3 (d, *J* = 21.5 Hz), 49.4, 43.8, 32.8, 26.1, 25.6.

HRMS (EI) m/z: $[M]^+$ Calcd for $C_{13}H_{17}FS_2$ 256.0750; found 256.0750.

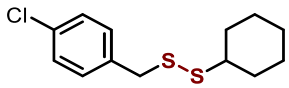

4f

1-(4-Chlorobenzyl)-2-cyclohexyldisulfane (**4f**):

Following the General Procedure A and purification via column chromatography on silica gel (pentane/EtOAc = 20/1, v/v), **4f** was obtained as a colorless oil (133.8 mg, 82% yield).

^{1}**H NMR** (300 MHz, $CDCl_3$) δ 7.35 – 7.23 (m, 4H), 3.86 (s, 2H), 2.52 – 2.37 (m, 1H), 2.03 – 1.91 (m, 2H), 1.85 – 1.73 (m, 2H), 1.68 – 1.56 (m, 1H), 1.36 – 1.18 (m, 5H). ^{13}**C NMR** (76 MHz, $CDCl_3$) δ 136.3, 133.2, 130.6, 128.6, 49.5, 43.8, 32.8, 26.1, 25.6.

HRMS (EI) m/z: $[M]^+$ Calcd for $C_{13}H_{17}ClS_2$ 272.0455; found 272.0455.

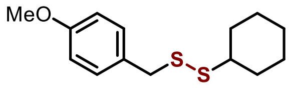

4g

1-Cyclohexyl-2-(4-methoxybenzyl)disulfane (**4g**):

Following the General Procedure A and purification via column chromatography on silica gel (pentane/EtOAc = 20/1, v/v), **4g** was obtained as a colorless oil (133.5 mg, 83% yield).

^{1}H NMR (300 MHz, $CDCl_3$) δ 7.24 (d, *J* = 9.1 Hz, 2H), 6.85 (d, *J* = 8.7 Hz, 2H), 3.86 (s, 2H), 3.80 (s, 3H), 2.43 (td, *J* = 10.5, 3.8 Hz, 1H), 1.96 (d, *J* = 11.3 Hz, 2H), 1.75 (s, 2H), 1.58 (s, 1H), 1.35 – 1.14 (m, 5H). **^{13}C NMR** (76 MHz, $CDCl_3$) δ 159.0, 130.4, 129.6, 113.9, 55.3, 49.4, 44.2, 32.8, 26.1, 25.7.
The NMR spectra were in agreement with those reported in the literature.[16]

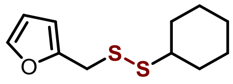

4h

2-((Cyclohexyldisulfaneyl)methyl)furan (**4h**):
Following the General Procedure A and purification via column chromatography on silica gel (pentane/EtOAc = 20/1, v/v), **4h** was obtained as a colorless oil (97.1 mg, 71% yield).
^{1}H NMR (400 MHz, $CDCl_3$) δ 7.38 (dd, *J* = 1.9, 0.9 Hz, 1H), 6.32 (dd, *J* = 3.2, 1.9 Hz, 1H), 6.28 – 6.21 (m, 1H), 3.89 (s, 2H), 2.42 (tt, *J* = 10.7, 3.9 Hz, 1H), 2.00 – 1.91 (m, 2H), 1.80 – 1.70 (m, 2H), 1.63 – 1.54 (m, 1H), 1.32 – 1.15 (m, 5H). **^{13}C NMR** (101 MHz, $CDCl_3$) δ 150.7, 142.4, 110.7, 108.7, 49.5, 36.9, 32.7, 26.1, 25.6.
The NMR spectra were in agreement with those reported in the literature.[1]

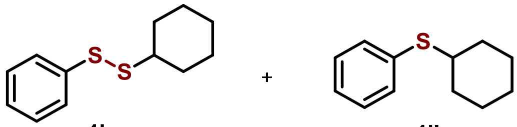

4i + **4i'**

Following the General Procedure B and purification via column chromatography on silica gel (pentane/EtOAc = 20/1, v/v), the disulfide **4i** was obtained as a colorless oil (34.9 mg, 26% yield), along with the monosulfide **4i'** (a colorless oil, 42.6 mg, 37% yield).

1-Cyclohexyl-2-phenyldisulfane (**4i**):
^{1}H NMR (400 MHz, $CDCl_3$) δ 7.56 – 7.52 (m, 2H), 7.34 – 7.28 (m, 2H), 7.23 – 7.17 (m, 1H), 2.81 (tt, *J* = 10.8, 3.7 Hz, 1H), 2.07 – 1.97 (m, 2H), 1.77 (dq, *J* = 12.1, 4.0 Hz, 2H), 1.60 (ddt, *J* = 10.5, 5.6, 1.6 Hz, 1H), 1.39 – 1.18 (m, 5H). **^{13}C NMR** (101 MHz, $CDCl_3$) δ 138.5, 128.8, 126.8, 126.3, 49.9, 32.9, 32.6, 26.0, 25.6.
The NMR spectra were in agreement with those reported in the literature.[1]

Cyclohexyl(phenyl)sulfane (**4i'**):
^{1}H NMR (300 MHz, $CDCl_3$) δ 7.46 – 7.38 (m, 2H), 7.34 – 7.27 (m, 2H), 7.26 – 7.20 (m, 1H), 3.26 – 3.00 (m, 1H), 2.08 – 1.92 (m, 2H), 1.85 – 1.74 (m, 2H), 1.69 – 1.59 (m, 1H), 1.48 – 1.22 (m, 5H). **^{13}C NMR** (101 MHz, $CDCl_3$) δ 135.2, 131.9, 128.8, 126.6, 46.6, 33.4, 26.1, 25.8.
The NMR spectra were in agreement with those reported in the literature.[17]

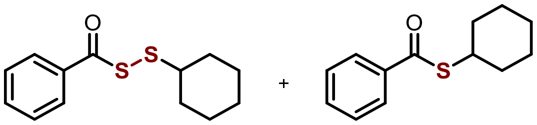

4j + **4j´**

Following the General Procedure B and purification via column chromatography on silica gel (pentane/EtOAc = 10/1, v/v), **4j** was obtained as a colorless oil (62.0 mg, 41% yield), along with the monosulfide **4j'** (colorless oil, 48.8 mg, 37% yield).

SS-cyclohexyl benzo(dithioperoxoate) (**4j**):

^{1}H NMR (300 MHz, $CDCl_3$) δ 8.06 – 7.97 (m, 2H), 7.66 – 7.57 (m, 1H), 7.51 – 7.43 (m, 2H), 2.87 (tt, *J* = 10.8, 3.7 Hz, 1H), 2.10 – 1.99 (m, 2H), 1.85 – 1.74 (m, 2H), 1.65 – 1.58 (m, 1H), 1.46 – 1.22 (m, 5H). **^{13}C NMR** (76 MHz, $CDCl_3$) δ 190.8, 135.9, 133.9, 128.9, 127.8, 49.7, 32.6, 26.0, 25.5.

The NMR spectra were in agreement with those reported in the literature.[18]

S-cyclohexyl benzothioate (**4j'**):

^{1}H NMR (300 MHz, $CDCl_3$) δ 8.00 – 7.90 (m, 2H), 7.60 – 7.49 (m, 1H), 7.43 (ddd, *J* = 8.4, 6.6, 1.4 Hz, 2H), 3.82 – 3.65 (m, 1H), 2.03 (t, *J* = 4.8 Hz, 2H), 1.81 – 1.70 (m, 2H), 1.62 (t, *J* = 10.0 Hz, 1H), 1.56 – 1.42 (m, 4H), 1.38 – 1.28 (m, 1H).

The NMR spectra were in agreement with those reported in the literature.[19]

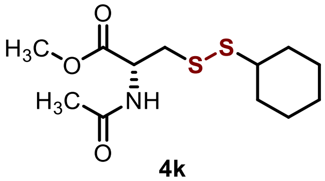

4k

Methyl N-acetyl-S-(cyclohexylthio)-L-cysteinate (**4k**):

Following the General Procedure A and purification via column chromatography on silica gel (pentane/EtOAc = 100/1, v/v), **4k** was obtained as a colorless oil (96.0 mg, 51% yield).

^{1}H NMR (400 MHz, $CDCl_3$) δ 6.34 (d, *J* = 7.6 Hz, 1H), 4.88 (dt, *J* = 7.7, 4.9 Hz, 1H), 3.77 (s, 3H), 3.17 (t, *J* = 5.0 Hz, 2H), 2.76 – 2.68 (m, 1H), 2.05 (s, 3H), 2.03 – 1.95 (m, 2H), 1.82 – 1.73 (m, 2H), 1.62 (d, *J* = 5.3 Hz, 1H), 1.36 – 1.23 (m, 5H). **^{13}C NMR** (101 MHz, $CDCl_3$) δ 171.0, 169.8, 52.7, 51.9, 49.6, 41.5, 32.6, 26.0, 25.6, 23.2.

HRMS (ESI) m/z: $[M + Na]^+$ Calcd for $C_{12}H_{21}NO_3S_2Na$ 314.0855; found 314.0855.

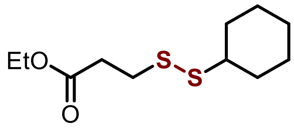

4k'

Ethyl 3-(cyclohexyldisulfaneyl)propanoate (**4k'**):

Following the General Procedure A and purification via column chromatography on silica gel (pentane/EtOAc = 100/1, v/v), **4k'** was obtained as a colorless oil (107.0 mg, 72% yield).

^{1}H NMR (300 MHz, $CDCl_3$) δ 4.19 – 3.99 (m, 2H), 2.88 – 2.75 (m, 2H), 2.73 – 2.57 (m, 3H), 2.02 – 1.86 (m, 2H), 1.72 (dd, J = 8.5, 3.8 Hz, 2H), 1.59 – 1.49 (m, 1H), 1.31 – 1.15 (m, 8H). **^{13}C NMR** (76 MHz, $CDCl_3$) δ 171.9, 60.7, 49.5, 34.3, 33.6, 32.8, 26.1, 25.6, 14.2.

HRMS (ESI) m/z: $[M + Na]^+$ Calcd for $C_{11}H_{20}O_2S_2Na$ 271.0797; found 271.0797.

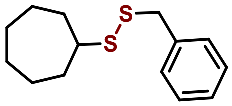

4l

1-Benzyl-2-cycloheptyldisulfane (**4l**):

Following the General Procedure A and purification via column chromatography on silica gel (pentane/EtOAc = 20/1, v/v), **4l** was obtained as a colorless oil (111.9 mg, 74% yield).

^{1}H NMR (300 MHz, $CDCl_3$) δ 7.38 – 7.29 (m, 5H), 3.89 (s, 2H), 2.43 (dt, J = 9.9, 5.4 Hz, 1H), 2.02 – 1.87 (m, 2H), 1.74 – 1.61 (m, 3H), 1.52 – 1.37 (m, 7H). **^{13}C NMR** (76 MHz, $CDCl_3$) δ 137.8, 129.3, 128.5, 127.3, 50.6, 44.2, 34.1, 28.1, 26.1.

HRMS (EI) m/z: $[M]^+$ Calcd for $C_{14}H_{20}S_2$ 252.1001; found 252.1001.

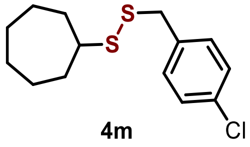

4m

1-(4-Chlorobenzyl)-2-cycloheptyldisulfane (**4m**):

Following the General Procedure A and purification via column chromatography on silica gel (pentane/EtOAc = 20/1, v/v), **4m** was obtained as a colorless oil (130.4 mg, 76% yield).

^{1}H NMR (300 MHz, $CDCl_3$) δ 7.32 – 7.27 (m, 2H), 7.27 – 7.22 (m, 2H), 3.84 (s, 2H), 2.44 (td, J = 9.4, 4.8 Hz, 1H), 1.97 (ddt, J = 13.7, 6.7, 3.2 Hz, 2H), 1.74 – 1.62 (m, 2H), 1.52 – 1.26 (m, 8H). **^{13}C NMR** (76 MHz, $CDCl_3$) δ 136.4, 133.2, 130.6, 128.6, 50.8, 43.3, 34.1, 28.1, 26.1.

HRMS (EI) m/z: $[M]^+$ Calcd for $C_{14}H_{19}ClS_2$ 286.0611; found 286.0611.

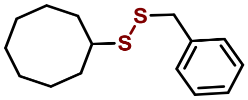

4n

1-Benzyl-2-cyclooctyldisulfane (**4n**):

Following the General Procedure A and purification via column chromatography on silica gel (pentane/EtOAc = 20/1, v/v), **4n** was obtained as a colorless oil (119.7 mg, 75% yield).

^{1}H NMR (300 MHz, $CDCl_3$) δ 7.34 – 7.21 (m, 5H), 3.81 (s, 2H), 2.40 (tt, *J* = 9.3, 3.7 Hz, 1H), 1.81 (ddt, *J* = 14.6, 8.1, 3.2 Hz, 2H), 1.63 – 1.25 (m, 14H). **^{13}C NMR** (76 MHz, $CDCl_3$) δ 137.9, 129.3, 128.5, 127.4, 49.8, 44.2, 31.8, 26.8, 25.9, 25.4.

HRMS (EI) m/z: $[M\ ]^+$ Calcd for $C_{15}H_{22}S_2$ 266.1157; found 266.1157.

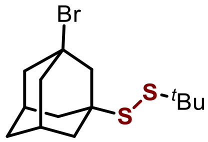

4o

3-Bromoadamantan-1-yl)-2-(tert-butyl)disulfane (**4o**):

Following the General Procedure C and purification via column chromatography on silica gel (pentane/EtOAc = 20/1, v/v), **4o** was obtained as a colorless oil (51.3 mg, 51% yield).

^{1}H NMR (400 MHz, $CDCl_3$) δ 2.45 – 2.41 (m, 2H), 2.32 – 2.24 (m, 6H), 1.89 – 1.78 (m, 4H), 1.68 (d, *J* = 2.7 Hz, 2H), 1.33 (s, 9H). **^{13}C NMR** (76 MHz, $CDCl_3$) δ 64.5, 54.1, 49.8, 47.7, 46.4, 41.1, 34.2, 33.4, 30.5.

HRMS (EI) m/z: $[M]^+$ Calcd for $C_{14}H_{23}BrS_2$ 334.0419; found 334.0419.

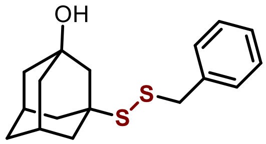

4p

3-(Benzyldisulfaneyl)adamantan-1-ol (**4p**):

Following the General Procedure C and purification via column chromatography on silica gel (pentane/EtOAc = 5/1, v/v), **4p** was obtained as a colorless oil (61.5 mg, 67% yield).

^{1}H NMR (300 MHz, $CDCl_3$) δ 7.32 (d, *J* = 2.7 Hz, 5H), 3.90 (s, 2H), 2.34 – 2.21 (m, 2H), 1.79 (s, 2H), 1.77 – 1.72 (m, 3H), 1.68 (d, *J* = 3.0 Hz, 3H), 1.60 (s, 2H), 1.56 (d, *J* = 3.5 Hz, 2H). **^{13}C NMR** (76 MHz, $CDCl_3$) δ 137.4, 129.3, 128.6, 127.5, 69.6, 50.9, 50.2, 46.1, 43.9, 41.3, 34.7, 31.5.

HRMS (ESI) m/z: $[M + Na]^+$ Calcd for $C_{17}H_{22}OS_2Na$ 329.1004; found 329.1004.

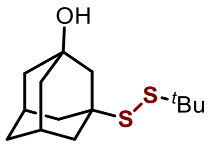

4q

3-(Tert-butyldisulfaneyl)adamantan-1-ol (**4q**):

Following the General Procedure C and purification via column chromatography on silica gel (pentane/EtOAc = 5/1, v/v) and preparative RP-MPLC (acetonitrile/water, gradient), **4q** was obtained as a colorless oil (53.8 mg, 66% yield).

^{1}H NMR (300 MHz, $CDCl_3$) δ 2.33 – 2.23 (m, 2H), 1.79 (s, 2H), 1.72 (d, J = 3.0 Hz, 4H), 1.66 (d, J = 3.0 Hz, 4H), 1.54 (q, J = 2.7 Hz, 2H), 1.30 (s, 9H). **^{13}C NMR** (76 MHz, $CDCl_3$) δ 69.7, 50.7, 49.1, 46.1, 43.9, 41.8, 34.7, 31.6, 30.6.

HRMS (ESI) m/z: $[M + Na]^+$ Calcd for $C_{14}H_{24}OS_2Na$ 295.1161; found 295.1160.

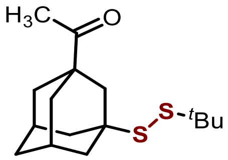

4r

1-(3-(Tert-butyldisulfaneyl)adamantan-1-yl)ethan-1-one (**4r**):

Following the General Procedure C and purification via column chromatography on silica gel (pentane/EtOAc = 20/1, v/v) and preparative RP-MPLC (acetonitrile/water, gradient), **4r** was obtained as a colorless oil (60.8 mg, 68% yield). The regioselectivity was determined by ^{1}H NMR spectroscopy.

^{1}H NMR (400 MHz, $CDCl_3$) δ 2.15 (p, J = 3.1 Hz, 2H), 2.03 (s, 3H), 1.80 (s, 2H), 1.73 (d, J = 3.1 Hz, 4H), 1.66 (d, J = 3.1 Hz, 4H), 1.57 (dt, J = 5.5, 2.8 Hz, 2H), 1.23 (s, 9H). **^{13}C NMR** (101 MHz, $CDCl_3$) δ 212.6, 48.7, 47.4, 46.1, 43.8, 42.1, 37.0, 35.1, 30.5, 29.6, 24.5.

HRMS (ESI) m/z: $[M + Na]^+$ Calcd for $C_{16}H_{26}OS_2Na$ 321.1317; found 321.1317.

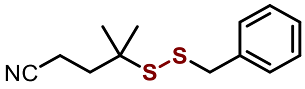

4s

4-(Benzyldisulfaneyl)-4-methylpentanenitrile (**4s**):

Following the General Procedure D and purification via column chromatography on silica gel (pentane/EtOAc = 10/1, v/v), **4s** was obtained as a colorless oil (69.3 mg, 46% yield). The regioselectivity was determined by ^{1}H NMR spectroscopy.

^{1}H NMR (400 MHz, $CDCl_3$) δ 7.35 – 7.28 (m, 5H), 3.93 (s, 2H), 2.45 – 2.37 (m, 2H), 2.00 – 1.91 (m, 2H), 1.31 (s, 6H). **^{13}C NMR** (101 MHz, $CDCl_3$) δ 135.8, 128.2, 127.6, 126.7, 118.8, 48.9, 44.3, 35.7, 26.4, 12.1.

HRMS (EI) m/z: $[M]^+$ Calcd for $C_{13}H_{17}NS_2$ 251.0797; found 251.0797.

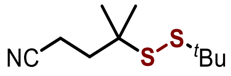

4t

4-(Tert-butyldisulfaneyl)-4-methylpentanenitrile (**4t**):

Following the General Procedure D and purification via column chromatography on silica gel (pentane/EtOAc = 10/1, v/v), **4t** was obtained as a colorless oil (70.3 mg, 54% yield). The regioselectivity was determined by ^1H NMR spectroscopy.

^{1}H NMR (400 MHz, $CDCl_3$) δ 2.49 – 2.42 (m, 2H), 1.96 – 1.88 (m, 2H), 1.32 (s, 9H), 1.31 (s, 6H). **^{13}C NMR** (101 MHz, $CDCl_3$) δ 119.9, 48.2, 46.8, 37.5, 30.6, 27.8, 13.1.

HRMS (EI) m/z: $[M]^+$ Calcd for $C_{10}H_{19}NS_2$ 217.0953; found 217.0953.

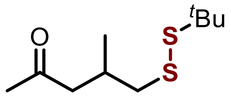

4u

5-(Tert-butyldisulfaneyl)-4-methylpentan-2-one (**4u**):

Following the General Procedure D and purification via column chromatography on silica gel (pentane/EtOAc = 5/1, v/v), **4u** was obtained as a colorless oil (66.0 mg, 50% yield). The regioselectivity was determined by ^1H NMR spectroscopy.

^{1}H NMR (400 MHz, $CDCl_3$) δ 2.73 – 2.64 (m, 3H), 2.39 – 2.26 (m, 2H), 2.15 (s, 3H), 1.33 (s, 9H), 1.02 (d, *J* = 6.5 Hz, 3H). **^{13}C NMR** (101 MHz, $CDCl_3$) δ 208.0, 49.1, 48.0, 47.9, 30.4, 29.9, 29.4, 19.4.

HRMS (ESI) m/z: $[M + Na]^+$ Calcd for $C_{10}H_{20}OS_2Na$ 243.0848; found 243.0848.

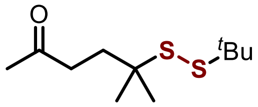

4u´

5-(Tert-butyldisulfaneyl)-5-methylhexan-2-one (**4u'**):

Following the General Procedure D and purification via column chromatography on silica gel (pentane/EtOAc = 5/1, v/v), **4u'** was obtained as a colorless oil (59.0 mg, 42% yield). The regioselectivity was determined by ^1H NMR spectroscopy.

^{1}H NMR (400 MHz, $CDCl_3$) δ 2.59 – 2.52 (m, 2H), 2.16 (s, 3H), 1.85 – 1.79 (m, 2H), 1.30 (s, 9H), 1.26 (s, 6H). **^{13}C NMR** (101 MHz, $CDCl_3$) δ 208.4, 48.8, 46.4, 39.4, 35.5, 30.6, 29.9, 28.3.

HRMS (ESI) m/z: $[M + Na]^+$ Calcd for $C_{11}H_{22}OS_2Na$ 257.1004; found 257.1004.

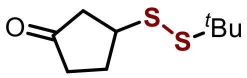

4v

3-(Tert-butyldisulfaneyl)cyclopentan-1-one (**4v**):

Following the General Procedure D and purification via column chromatography on silica gel (pentane/EtOAc = 5/1, v/v), **4v** was obtained as a colorless oil (88.1 mg, 72% yield).

When using alkane **2v** as the limiting reagent (**3a** 0.45 mmol, **2v** 0.3 mmol), **4v** was obtained in 21% GC yield with CH_2Br_2 as the internal standard.

^{1}H NMR (400 MHz, $CDCl_3$) δ 3.57 (dq, *J* = 7.9, 5.6 Hz, 1H), 2.61 – 2.52 (m, 1H), 2.46 – 2.36 (m, 2H), 2.36 – 2.28 (m, 1H), 2.24 – 2.08 (m, 2H), 1.33 (s, 9H). **^{13}C NMR** (101 MHz, $CDCl_3$) δ 216.5, 48.0, 46.4, 44.6, 36.6, 30.1, 30.1, 28.9.

HRMS (ESI) m/z: $[M + Na]^+$ Calcd for $C_9H_{16}OS_2Na$ 227.0535; found 227.0535.

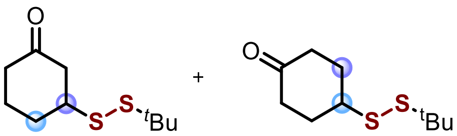

4w (major) **4w (minor)**

Following the General Procedure D and purification via column chromatography on silica gel (pentane/EtOAc = 5/1, v/v), **4w** was obtained (colorless oil, 107.2 mg, 82% yield) as the mixture of regioisomers. The ratio of the mixture was determined by ^{1}H NMR spectroscopy.

When using alkane **2w** as the limiting reagent (**3a** 0.45 mmol, **2w** 0.3 mmol), **4w** was obtained in 33% GC yield with CH_2Br_2 as the internal standard.

3-(Tert-butyldisulfaneyl)cyclohexan-1-one (**4w**, major):

^{1}H NMR (599 MHz, $CDCl_3$) δ 3.09 – 2.99 (m, 1H), 2.78 (ddt, *J* = 14.3, 4.6, 1.8 Hz, 1H), 2.39 – 2.33 (m, 2H), 2.33 – 2.27 (m, 2H), 2.16 – 2.09 (m, 1H), 1.74 – 1.65 (m, 2H), 1.31 (s, 9H). **^{13}C NMR** (151 MHz, $CDCl_3$) δ 208.6, 49.0, 47.7, 47.3, 40.9, 31.1, 30.0, 24.1.

4-(Tert-butyldisulfaneyl)cyclohexan-1-one (**4w**, minor):

^{1}H NMR (599 MHz, $CDCl_3$) δ 3.17 (tt, *J* = 8.0, 3.7 Hz, 1H), 2.58 – 2.49 (m, 2H), 2.27 – 2.22 (m, 2H), 2.16 – 2.10 (m, 2H), 2.01 – 1.91 (m, 2H), 1.35 (s, 9H). **^{13}C NMR** (151 MHz, $CDCl_3$) δ 210.1, 47.9, 46.7, 39.1, 31.4, 30.0.

HRMS (ESI) m/z: $[M + Na]^+$ Calcd for $C_{10}H_{18}OS_2Na$ 241.0691; found 241.0691.

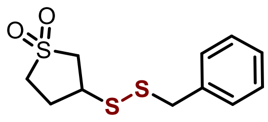

4x

3-(Benzyldisulfaneyl)tetrahydrothiophene 1,1-dioxide (**4x**):

Following the General Procedure A and purification via column chromatography on silica gel (pentane/EtOAc = 5/1, v/v), **4x** was obtained as a colorless oil (11.8 mg, 68% yield).

^{1}H NMR (300 MHz, $CDCl_3$) δ 7.35 – 7.20 (m, 5H), 3.84 (s, 2H), 3.13 (ddd, J = 13.2, 8.1, 5.1 Hz, 1H), 3.06 – 2.95 (m, 2H), 2.93 – 2.72 (m, 2H), 2.24 (dt, J = 12.5, 6.5 Hz, 1H), 2.08 – 1.91 (m, 1H). **^{13}C NMR** (76 MHz, $CDCl_3$) δ 136.9, 129.4, 128.8, 128.0, 55.8, 51.6, 44.0, 43.8, 28.8.

HRMS (ESI) m/z: $[M + Na]^+$ Calcd for $C_{11}H_{14}O_2S_3Na$ 297.0048; found 297.0048.

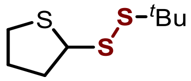

4y

2-(Tert-butyldisulfaneyl)tetrahydrothiophene (**4y**):

Following the General Procedure A and purification via column chromatography on silica gel (pentane/EtOAc = 10/1, v/v), **4y** was obtained as a colorless oil (77.4 mg, 62% yield).

^{1}H NMR (400 MHz, $CDCl_3$) δ 4.48 (dd, J = 5.3, 1.9 Hz, 1H), 3.08 – 2.98 (m, 1H), 2.82 (td, J = 10.0, 6.7 Hz, 1H), 2.56 – 2.47 (m, 1H), 2.16 – 1.99 (m, 3H), 1.35 (s, 9H). **^{13}C NMR** (101 MHz, $CDCl_3$) δ 58.7, 48.2, 37.8, 33.4, 30.2, 27.1.

HRMS (EI) m/z: $[M]^+$ Calcd for $C_8H_{16}S_3$ 208.0409; found 208.0409.

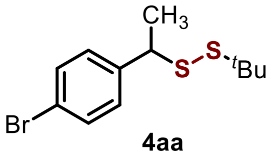

4aa

1-(1-(4-Bromophenyl)ethyl)-2-(tert-butyl)disulfane (**4aa**):

Following the General Procedure E (reaction temperature: room temperature) and purification via column chromatography on silica gel (pentane/EtOAc = 10/1, v/v), **4aa** was obtained as a colorless oil (74.8 mg, 82% yield).

^{1}H NMR (400 MHz, $CDCl_3$) δ 7.49 – 7.45 (m, 2H), 7.24 – 7.19 (m, 2H), 3.98 (q, J = 7.0 Hz, 1H), 1.66 (d, J = 7.0 Hz, 3H), 1.33 (s, 9H). **^{13}C NMR** (101 MHz, $CDCl_3$) δ 141.6, 131.6, 129.3, 121.3, 50.5, 48.0, 30.1, 21.1.

HRMS (EI) m/z: $[M]^+$ Calcd for $C_{12}H_{17}BrS_2$ 303.9955; found 303.9955.

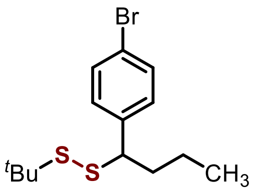

4bb

1-(1-(4-Bromophenyl)butyl)-2-(tert-butyl)disulfane (**4bb**):

Following the General Procedure E (reaction temperature: room temperature) and purification via column chromatography on silica gel (pentane/EtOAc = 10/1, v/v), **4bb** was obtained as a colorless oil (85.6 mg, 86% yield).

^{1}H NMR (300 MHz, $CDCl_3$) δ 7.44 (d, *J* = 8.4 Hz, 2H), 7.14 (d, *J* = 8.4 Hz, 2H), 3.75 (dd, *J* = 9.7, 5.6 Hz, 1H), 2.12 – 1.97 (m, 1H), 1.88 – 1.74 (m, 1H), 1.41 (dd, *J* = 4.0, 2.6 Hz, 1H), 1.36 (d, *J* = 4.2 Hz, 1H), 1.29 (s, 9H), 0.89 (t, *J* = 7.3 Hz, 3H). **^{13}C NMR** (76 MHz, $CDCl_3$) δ 140.8, 131.6, 130.1, 129.8, 121.1, 56.2, 47.9, 37.7, 30.1, 20.9, 13.7.

HRMS (EI) m/z: $[M]^+$ Calcd for $C_{14}H_{21}S_2$ 332.0268; found 332.0268.

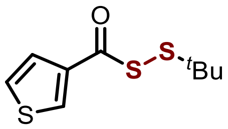

6a

SS-(tert-butyl) thiophene-3-carbo(dithioperoxoate) (**6a**):

Following the General Procedure E and purification via column chromatography on silica gel (pentane/EtOAc = 10/1, v/v), **6a** was obtained as a yellow oil (61.9 mg, 89% yield).

^{1}H NMR (300 MHz, $CDCl_3$) δ 8.25 (dd, *J* = 2.9, 1.3 Hz, 1H), 7.60 (dd, *J* = 5.1, 1.3 Hz, 1H), 7.39 (dd, *J* = 5.1, 2.9 Hz, 1H), 1.36 (s, 9H). **^{13}C NMR** (101 MHz, $CDCl_3$) δ 183.8, 139.1, 131.8, 126.8, 126.3, 49.1, 29.8.

HRMS (ESI) m/z: $[M + Na]^+$ Calcd for $C_9H_{12}OS_3Na$ 254.9942; found 254.9942.

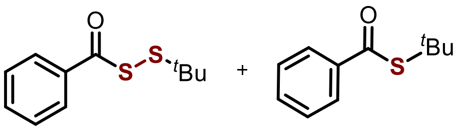

6b **6b'**

Following the General Procedure E and purification via column chromatography on silica gel (pentane/EtOAc = 10/1, v/v), **6b** was obtained as a yellow oil (20.3 mg, 30% yield), along with the monosulfide **6b'** (yellow oil, 29.1 mg, 51% yield).

When we shorten reaction time to 4 hours, the disulfide 6b was obtained as the single product (55.6 mg, 82% yield).

SS-(tert-butyl) benzo(dithioperoxoate) (**6b**):

^{1}H NMR (300 MHz, $CDCl_3$) δ 8.08 – 8.00 (m, 2H), 7.63 (t, J = 7.4 Hz, 1H), 7.49 (t, J = 7.8 Hz, 2H), 1.37 (s, 9H). The NMR spectra were in agreement with those reported in the literature.[1]

S-(tert-butyl) benzothioate (**6b'**):

^{1}H NMR (300 MHz, $CDCl_3$) δ 7.96 – 7.88 (m, 2H), 7.57 – 7.50 (m, 1H), 7.46 – 7.37 (m, 2H), 1.58 (s, 9H). The NMR spectra were in agreement with those reported in the literature.[19]

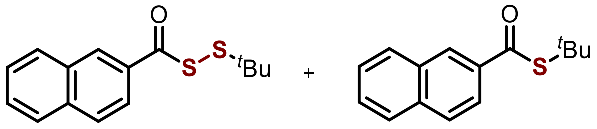

6c **6c'**

Following the General Procedure E and purification via column chromatography on silica gel (pentane/EtOAc = 5/1, v/v), **6c** was obtained as a colorless oil (30.1 mg, 46% yield), along with the monosulfide **6c'** (colorless oil, 22.7 mg, 31% yield).

When we shorten reaction time to 4 hours, the disulfide 6c was obtained as the single product (62.9 mg, 76% yield).

SS-(tert-butyl) naphthalene-2-carbo(dithioperoxoate) (**6c**):

^{1}H NMR (400 MHz, $CDCl_3$) δ 8.66 – 8.61 (m, 1H), 8.06 – 7.97 (m, 2H), 7.96 – 7.87 (m, 2H), 7.68 – 7.55 (m, 2H), 1.40 (s, 9H). **^{13}C NMR** (101 MHz, $CDCl_3$) δ 190.3, 136.0, 133.2, 132.4, 129.6, 129.5, 128.9, 128.8, 127.9, 127.1, 123.4, 49.2, 29.9.

HRMS (ESI) m/z: $[M + Na]^+$ Calcd for $C_{15}H_{16}OS_2Na$ 299.0535; found 299.0535.

S-(tert-butyl) naphthalene-2-carbothioate (**6c'**):

^{1}H NMR (400 MHz, $CDCl_3$) δ 8.49 (d, J = 1.8 Hz, 1H), 7.95 (dt, J = 8.5, 2.1 Hz, 2H), 7.86 (d, J = 8.8 Hz, 2H), 7.56 (dddd, J = 17.5, 8.0, 6.7, 1.4 Hz, 2H), 1.63 (s, 9H). **^{13}C NMR** (101 MHz, $CDCl_3$) δ 192.8, 135.6, 135.6, 132.5, 129.5, 128.3, 128.2, 128.2, 127.8, 126.8, 123.1, 48.3, 30.1.

The NMR spectra were in agreement with those reported in the literature.[20]

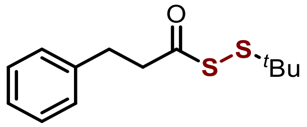

6d

SS-(tert-butyl) 3-phenylpropane(dithioperoxoate) (**6d**):

Following the General Procedure E and purification via column chromatography on silica gel (pentane/EtOAc = 5/1, v/v), **6d** was obtained as a colorless oil (62.5 mg, 82% yield).

^{1}H NMR (400 MHz, $CDCl_3$) δ 7.31 – 7.27 (m, 2H), 7.21 (td, *J* = 6.8, 1.7 Hz, 3H), 3.01 (s, 4H), 1.27 (s, 9H). **^{13}C NMR** (101 MHz, $CDCl_3$) δ 197.7, 139.7, 128.6, 128.4, 126.5, 48.8, 43.9, 31.4, 29.7.

HRMS (ESI) m/z: $[M + Na]^+$ Calcd for $C_{13}H_{18}OS_2Na$ 277.0691; found 277.0691.

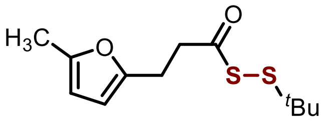

6e

SS-(tert-butyl) 3-(5-methylfuran-2-yl)propane(dithioperoxoate) (**6e**):

Following the General Procedure E and purification via column chromatography on silica gel (pentane/EtOAc = 5/1, v/v), **6e** was obtained as a colorless oil (38.7 mg, 50% yield).

^{1}H NMR (300 MHz, $CDCl_3$) δ 5.90 (d, *J* = 3.0 Hz, 1H), 5.83 (dd, *J* = 2.9, 1.2 Hz, 1H), 3.06 – 2.92 (m, 4H), 2.27 – 2.15 (m, 3H), 1.29 (s, 9H). **^{13}C NMR** (101 MHz, $CDCl_3$) δ 197.4, 151.3, 151.0, 106.5, 106.0, 48.8, 40.9, 29.7, 24.0, 13.5.

HRMS (ESI) m/z: $[M + Na]^+$ Calcd for $C_{12}H_{18}O_2S_2Na$ 281.0640; found 281.0640.

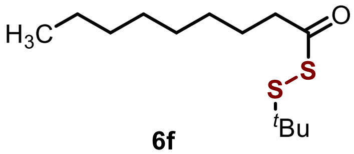

6f

SS-(Tert-butyl) nonane(dithioperoxoate) (**6f**):

Following the General Procedure E and purification via column chromatography on silica gel (pentane/EtOAc = 10/1, v/v), **6f** was obtained as a colorless oil (54.2 mg, 69% yield).

^{1}H NMR (400 MHz, $CDCl_3$) δ 2.68 (td, *J* = 7.5, 4.6 Hz, 2H), 1.69 (p, *J* = 7.5 Hz, 2H), 1.33 – 1.26 (s, 19H), 0.90 – 0.83 (m, 3H). **^{13}C NMR** (101 MHz, $CDCl_3$) δ 198.4, 48.7, 42.5, 31.8, 29.7, 29.2, 29.1, 29.0, 25.7, 22.6, 14.1.

HRMS (ESI) m/z: $[M + Na]^+$ Calcd for $C_{13}H_{26}OS_2Na$ 285.1317; found 285.1318.

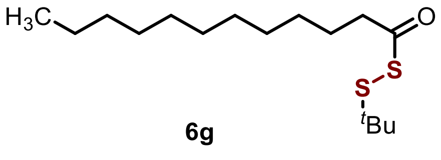

6g

SS-(tert-butyl) dodecane(dithioperoxoate) (**6g**):

Following the General Procedure E and purification via column chromatography on silica gel (pentane/EtOAc = 10/1, v/v), **6g** was obtained as a colorless oil (83.0 mg, 91% yield).

¹H NMR (400 MHz, $CDCl_3$) δ 2.73 – 2.65 (m, 2H), 1.69 (p, *J* = 7.3 Hz, 2H), 1.32 – 1.24 (m, 25H), 0.90 – 0.84 (m, 3H). **¹³C NMR** (101 MHz, $CDCl_3$) δ 198.4, 48.7, 42.5, 31.9, 29.7, 29.59, 29.56, 29.4, 29.3, 29.2, 29.0, 25.7, 22.7, 14.1.

HRMS (ESI) m/z: $[M + Na]^+$ Calcd for $C_{16}H_{32}OS_2Na$ 327.1787; found 327.1787.

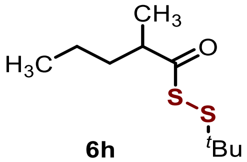

6h

SS-(tert-butyl) 2-methylpentane(dithioperoxoate) (**6h**):

Following the General Procedure E and purification via column chromatography on silica gel (pentane/EtOAc = 10/1, v/v), **6h** was obtained as a colorless oil (34.3 mg, 52% yield).

¹H NMR (400 MHz, $CDCl_3$) δ 2.86 (h, *J* = 6.9 Hz, 1H), 1.82 – 1.67 (m, 1H), 1.48 – 1.42 (m, 1H), 1.39 – 1.34 (m, 2H), 1.31 (s, 9H), 1.21 (d, *J* = 6.9 Hz, 3H), 0.92 (t, *J* = 7.2 Hz, 3H). **¹³C NMR** (101 MHz, $CDCl_3$) δ 202.1, 48.6, 47.4, 36.2, 29.7, 20.4, 17.8, 14.0.

HRMS (ESI) m/z: $[M + Na]^+$ Calcd for $C_{10}H_{20}OS_2Na$ 243.0848; found 243.0848.

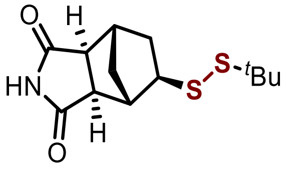

6j

(3aS,4S,5R,7S,7aS)-5-(Tert-butyldisulfaneyl)hexahydro-1H-4,7-methanoisoindole-1,3(2H)-dione (**6j**):

Following the General Procedure C and purification via column chromatography on silica gel (pentane/EtOAc = 2/1, v/v), **6j** was obtained as a colorless oil (61.6 mg, 72% yield).

¹H NMR (599 MHz, $CDCl_3$) δ 8.25 (s, 1H), 2.95 (s, 1H), 2.92 (ddd, *J* = 8.3, 4.5, 1.8 Hz, 1H), 2.76 (d, *J* = 4.4 Hz, 1H), 2.70 – 2.63 (m, 2H), 1.79 (ddd, *J* = 13.8, 8.4, 2.5 Hz, 1H), 1.73 (dp, *J* = 11.7, 1.5 Hz, 1H), 1.51 (dt, *J* = 13.8, 4.5 Hz, 1H), 1.34 (s, 9H), 1.27 (dt, *J* = 11.9, 2.4 Hz, 1H). **¹³C NMR** (151 MHz, $CDCl_3$) δ 178.2, 177.7, 51.9, 49.6, 49.4, 48.1, 45.2, 40.0, 36.2, 30.01, 30.00.

HRMS (ESI) m/z: $[M + Na]^+$ Calcd for $C_{13}H_{19}NO_2S_2Na$ 308.0749; found 308.0749.

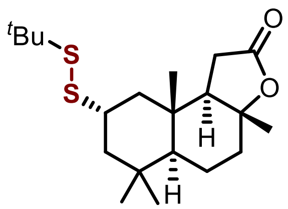

6k

(3aR,5aS,8S,9aS,9bR)-8-(Tert-butyldisulfaneyl)-3a,6,6,9a-tetramethyldecahydronaphtho[2,1-b]furan-2(1H)-one (**6k**):

Following the General Procedure C and purification via column chromatography on silica gel (pentane/EtOAc = 5/1, v/v), **6k** was obtained as a colorless oil (57.7 mg, 52% yield).

^{1}H NMR (500 MHz, $CDCl_3$) δ 3.00 (tt, *J* = 12.7, 3.8 Hz, 1H), 2.45 (dd, *J* = 16.2, 14.7 Hz, 1H), 2.29 (dd, *J* = 16.2, 6.5 Hz, 1H), 2.10 (dt, *J* = 11.6, 3.3 Hz, 1H), 2.01 (dd, *J* = 14.7, 6.5 Hz, 1H), 1.91 (dddd, *J* = 11.2, 5.6, 3.9, 2.1 Hz, 3H), 1.71 (td, *J* = 12.6, 4.4 Hz, 1H), 1.36 – 1.35 (m, 1H), 1.34 – 1.33 (m, 12H), 1.21 – 1.15 (m, 1H), 1.09 (dd, *J* = 12.6, 2.7 Hz, 1H), 1.06 – 1.00 (m, 1H), 0.96 (s, 3H), 0.93 (s, 3H), 0.87 (s, 3H). **^{13}C NMR** (126 MHz, $CDCl_3$) δ 176.3, 86.0, 58.8, 56.2, 48.5, 47.4, 46.1, 43.3, 38.5, 37.4, 34.9, 32.9, 30.1, 30.0, 28.7, 21.6, 21.3, 20.3, 15.7.

HRMS (ESI) m/z: $[M + Na]^+$ Calcd for $C_{20}H_{34}O_2S_2Na$ 393.1892; found 393.1892.

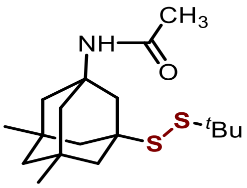

6l

N-(3-(tert-butyldisulfaneyl)-5,7-dimethyladamantan-1-yl)acetamide (**6l**):

Following the General Procedure C and purification via column chromatography on silica gel (pentane/EtOAc = 3/1, v/v) and preparative RP-MPLC (acetonitrile/water, gradient), **6l** was obtained as a colorless oil (72.6 mg, 71% yield). The regioselectivity was determined by ^{1}H NMR spectroscopy.

^{1}H NMR (400 MHz, $CDCl_3$) δ 5.12 (s, 1H), 1.86 (s, 2H), 1.84 (s, 3H), 1.55 (s, 4H), 1.43 (ddt, *J* = 12.4, 2.4, 1.4 Hz, 2H), 1.34 – 1.29 (m, 2H), 1.23 (s, 9H), 1.12 – 0.99 (m, 2H), 0.84 (s, 6H). **^{13}C NMR** (101 MHz, $CDCl_3$) δ 169.4, 54.7, 49.4, 48.8, 48.2, 46.4, 46.2, 45.6, 34.1, 30.6, 29.4, 24.6.

HRMS (ESI) m/z: $[M + Na]^+$ Calcd for $C_{18}H_{31}NOS_2Na$ 364.1739; found 364.1739.

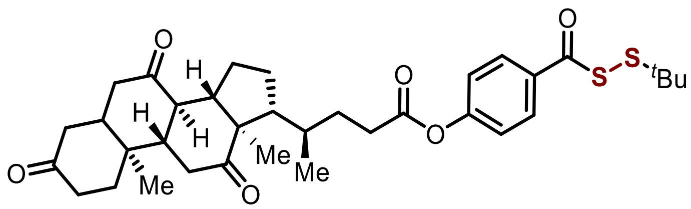

6m

4-(Tert-butyldisulfannecarbonyl)phenyl (4R)-4-((8R,9S,10S,13R,14S,17R)-10,13-dimethyl-3,7,12-trioxohexadecahydro-1H-cyclopenta[a]phenanthren-17-yl)pentanoate (**6m**):

Following the General Procedure E (**5m**, 0.2 mmol, 1.0 equiv., 5 h) and purification via column chromatography on silica gel (pentane/EtOAc = 2/1, v/v), **6m** was obtained as a white solid (100.1 mg, 80% yield).

^{1}H NMR (400 MHz, $CDCl_3$) δ 8.09 (d, *J* = 8.8 Hz, 2H), 7.23 (d, *J* = 8.7 Hz, 2H), 2.99 – 2.83 (m, 3H), 2.75 – 2.63 (m, 1H), 2.61 – 2.51 (m, 1H), 2.43 – 1.83 (m, 15H), 1.69 – 1.48 (m, 3H), 1.42 (s, 3H), 1.37 (s, 10H), 1.11 (s, 3H), 0.94 (d, *J* = 6.6 Hz, 3H). **^{13}C NMR** (101 MHz, $CDCl_3$) δ 211.9, 209.0, 208.7, 189.3, 171.9, 155.1, 133.3, 129.4, 122.1, 56.9, 51.8, 49.2, 49.0, 46.8, 45.60, 45.55, 45.0, 42.8, 38.7, 36.5, 36.0, 35.5, 35.3, 31.6, 30.3, 29.8, 27.7, 25.1, 21.9, 18.7, 11.9.

HRMS (ESI) m/z: $[M + Na]^+$ Calcd for $C_{35}H_{46}O_6S_2Na$ 649.2628; found 649.2628.

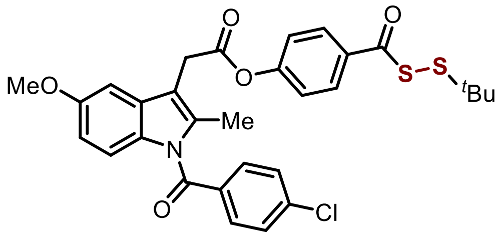

6n

4-(Tert-butyldisulfannecarbonyl)phenyl 2-(1-(4-chlorobenzoyl)-5-methoxy-2-methyl-1H-indol-3-yl)acetate (**6n**):

Following the General Procedure E (**5n**, 0.1 mmol, 1.0 equiv., room temperature, 12 h) and purification via column chromatography on silica gel (pentane/EtOAc = 8/1, v/v), **6n** was obtained as a yellow solid (24.4 mg, 42% yield).

^{1}H NMR (300 MHz, $CDCl_3$) δ 8.06 (d, *J* = 8.8 Hz, 2H), 7.68 (d, *J* = 8.5 Hz, 2H), 7.48 (d, *J* = 8.5 Hz, 2H), 7.20 (d, *J* = 8.7 Hz, 2H), 7.04 (d, *J* = 2.5 Hz, 1H), 6.88 (d, *J* = 9.0 Hz, 1H), 6.70 (dd, *J* = 9.1, 2.5 Hz, 1H), 3.94 (s, 2H), 3.84 (s, 3H), 2.47 (s, 3H), 1.35 (s, 9H). **^{13}C NMR** (101 MHz, $CDCl_3$) δ 189.3, 168.6, 168.3, 156.2, 155.0, 139.5, 136.4, 133.7, 133.5, 131.2, 130.9, 130.4, 129.4, 129.2, 122.0, 115.1, 111.8, 111.5, 101.2, 55.8, 49.2, 30.6, 29.8, 13.5.

HRMS (ESI) m/z: $[M + H]^+$ Calcd for $C_{30}H_{29}ClNO_5S_2$ 582.1170; found 582.1170.

2. Supplementary Figures

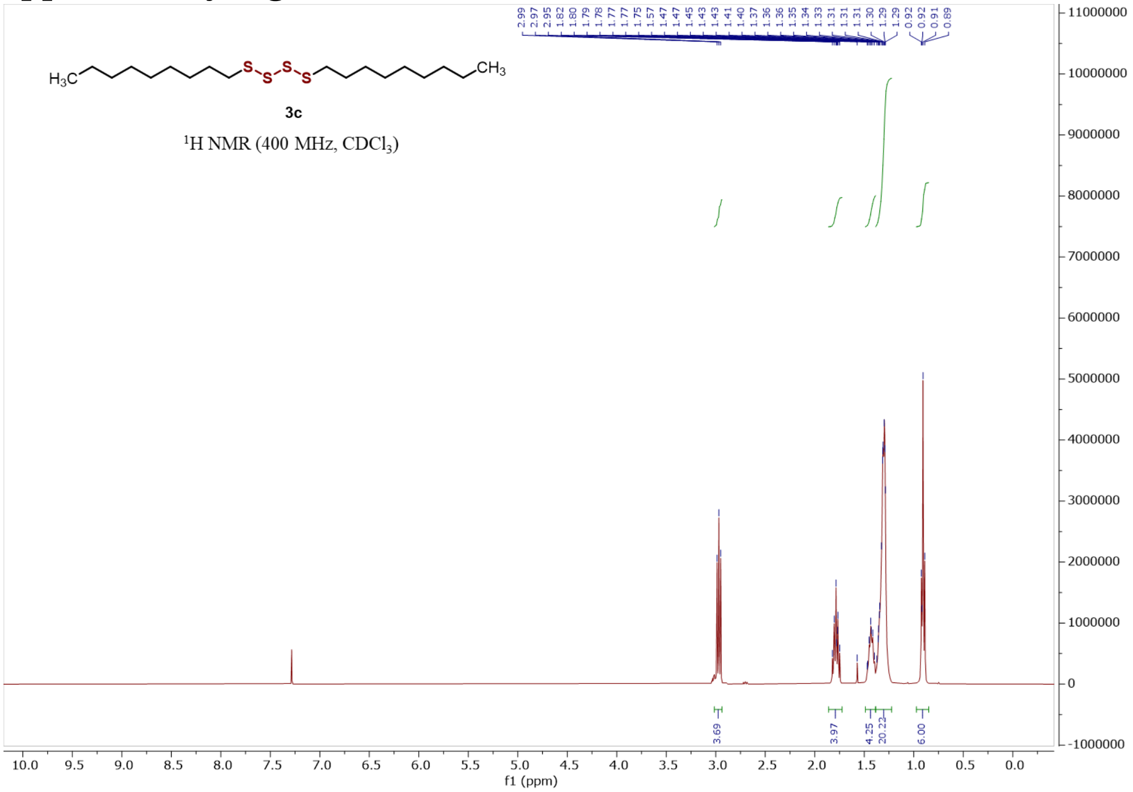

Supplementary Figure 2: ^{1}H NMR (400 MHz, CDCl$_3$) spectrum of compound **3c**.

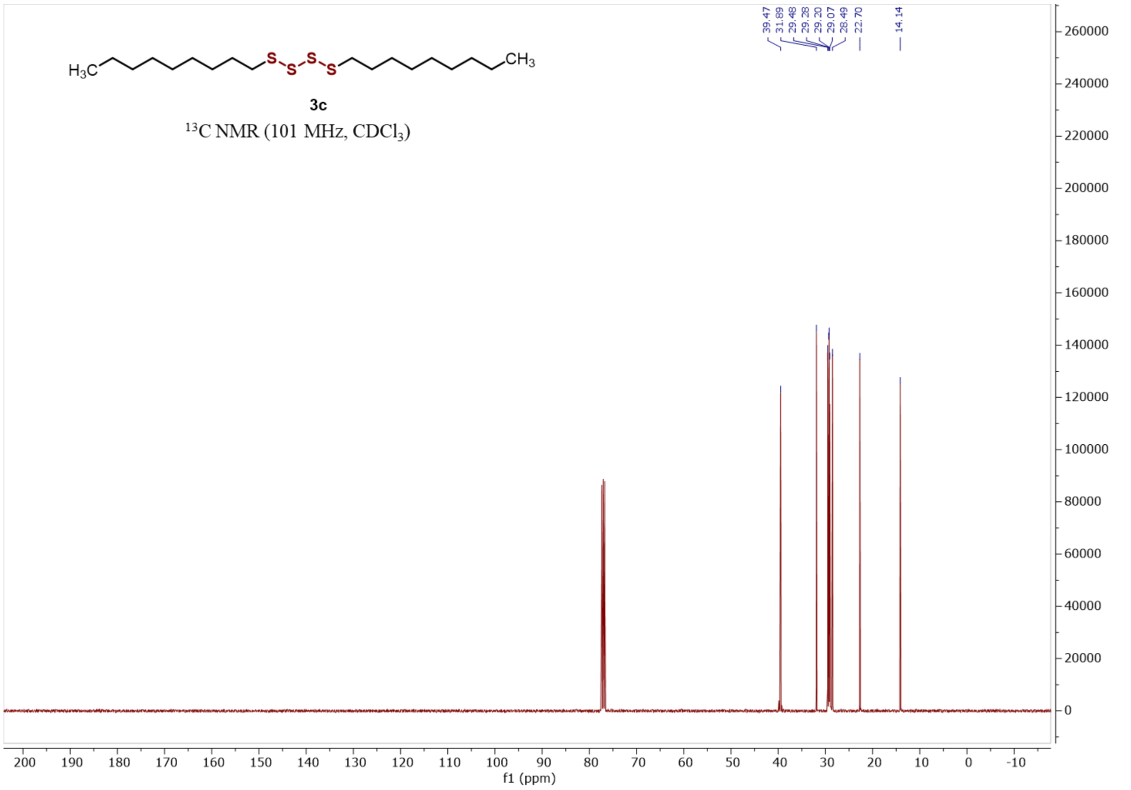

Supplementary Figure 3: ^{13}C NMR (101 MHz, CDCl$_3$) spectrum of compound **3c**.

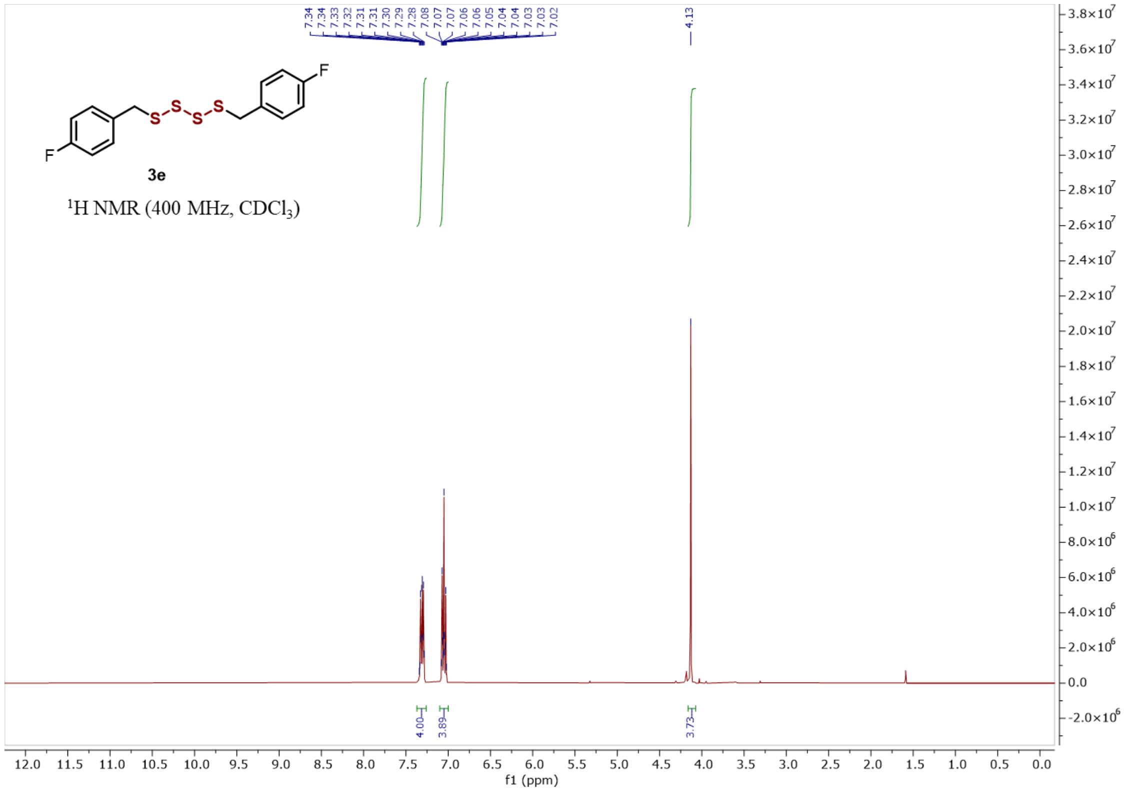

Supplementary Figure 4: ^{1}H NMR (400 MHz, $CDCl_3$) spectrum of compound **3e**.

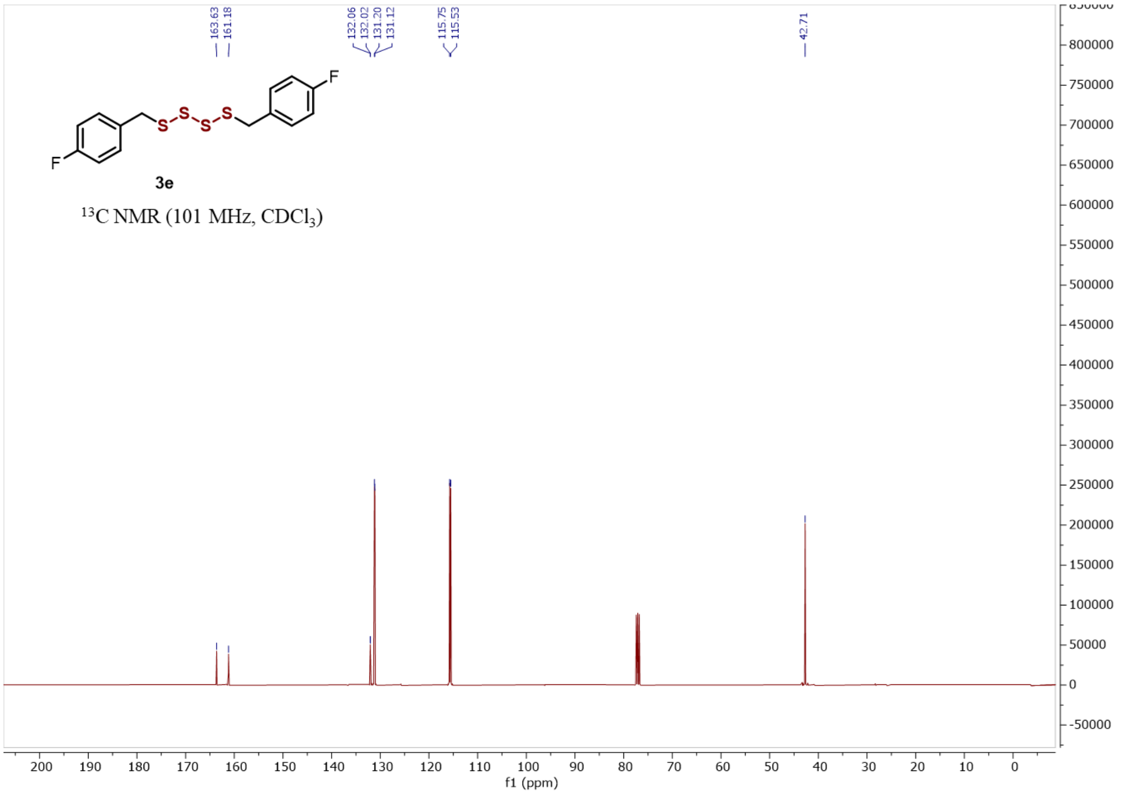

Supplementary Figure 5: ^{13}C NMR (101 MHz, $CDCl_3$) spectrum of compound **3e**.

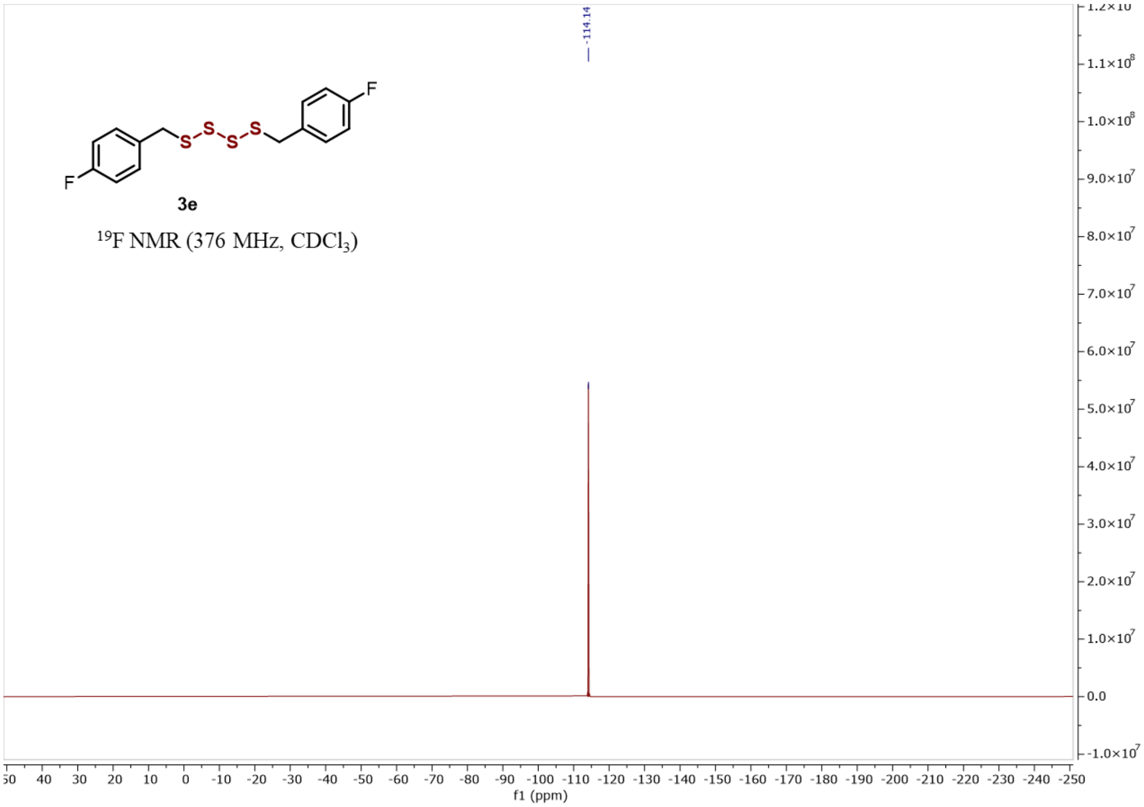

Supplementary Figure 6: ^{19}F NMR (376 MHz, $CDCl_3$) spectrum of compound **3e**.

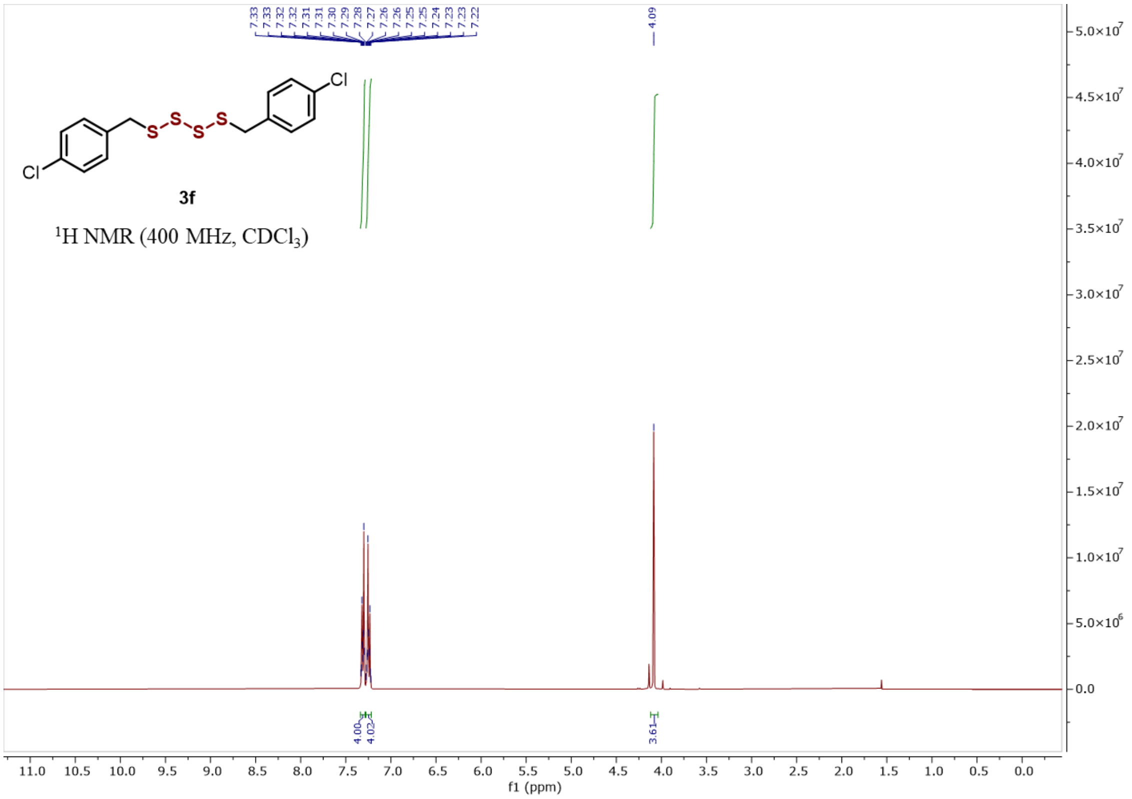

Supplementary Figure 7: ^{1}H NMR (400 MHz, $CDCl_3$) spectrum of compound **3f**.

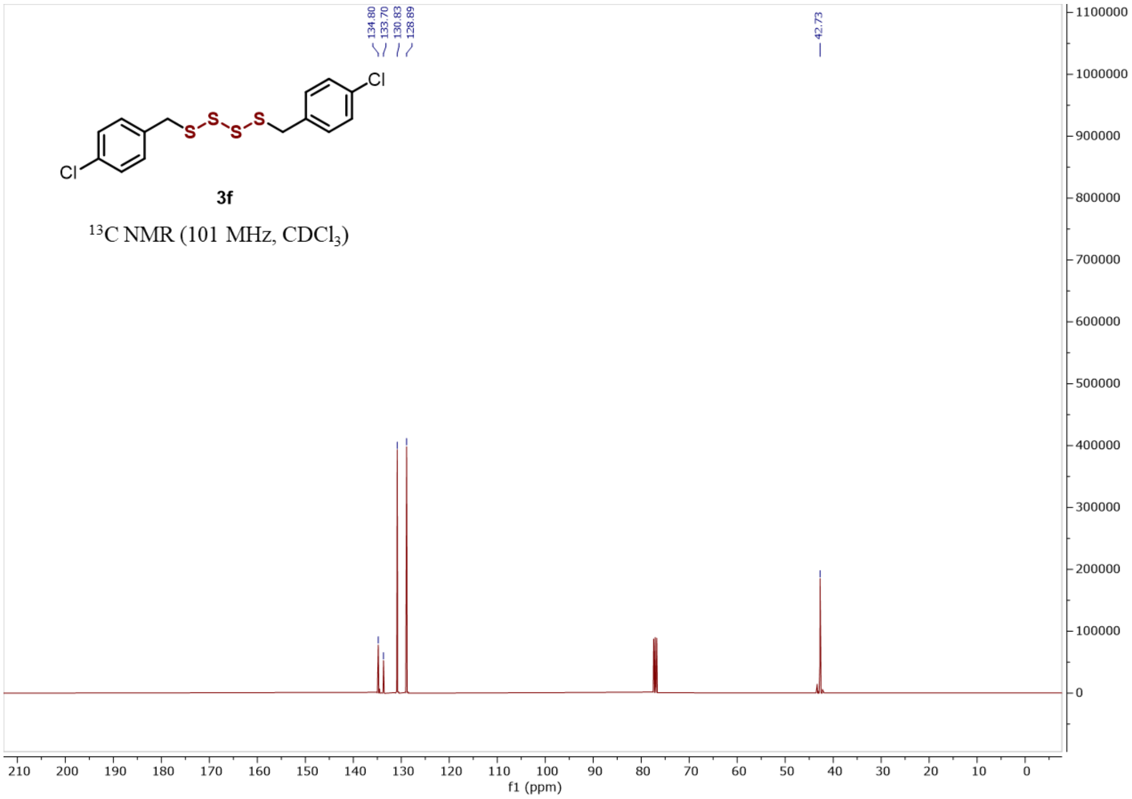

Supplementary Figure 8: ^{13}C NMR (101 MHz, $CDCl_3$) spectrum of compound **3f**.

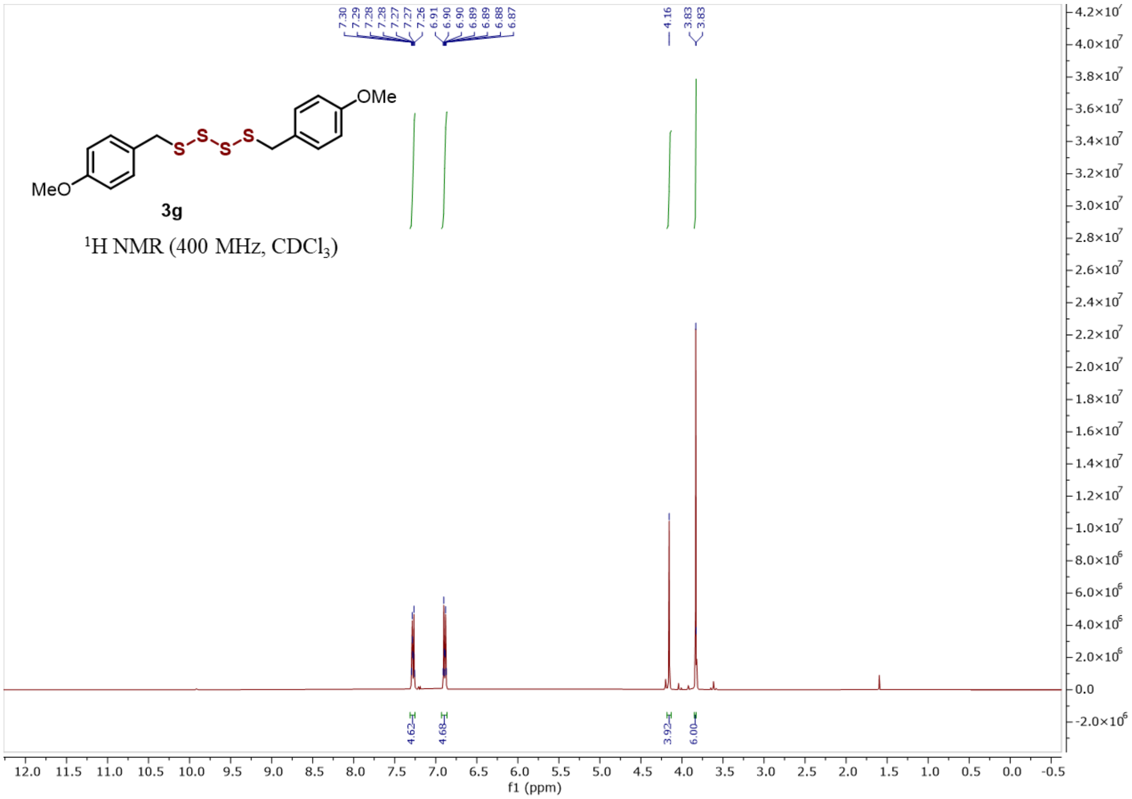

Supplementary Figure 9: ^{1}H NMR (400 MHz, $CDCl_3$) spectrum of compound **3g**.

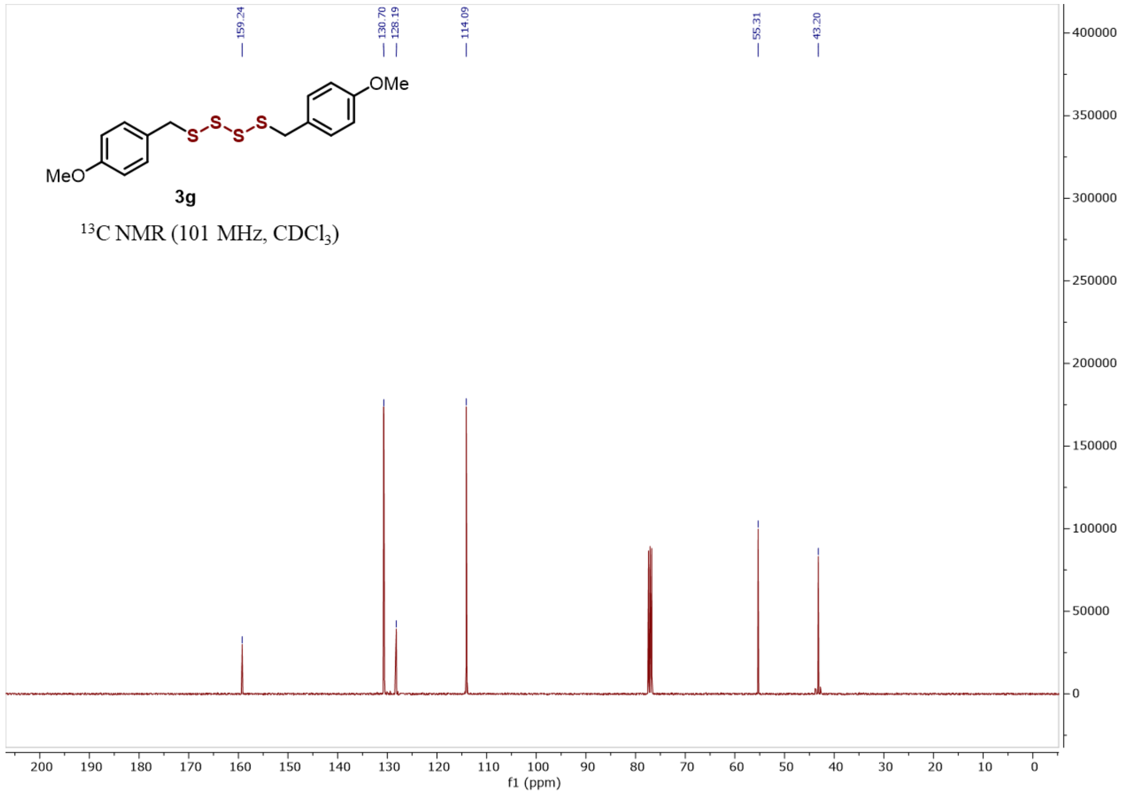

Supplementary Figure 10: ^{13}C NMR (101 MHz, $CDCl_3$) spectrum of compound **3g**.

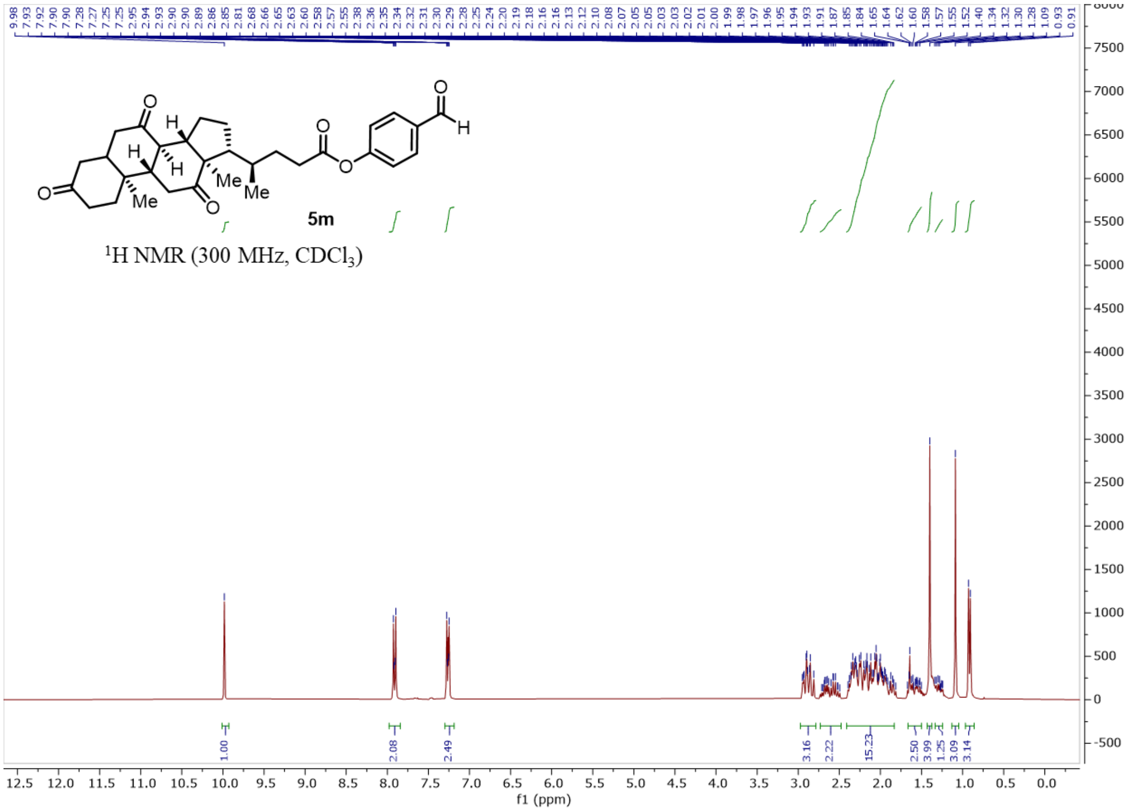

Supplementary Figure 11: ^{1}H NMR (300 MHz, $CDCl_3$) spectrum of compound **5m**.

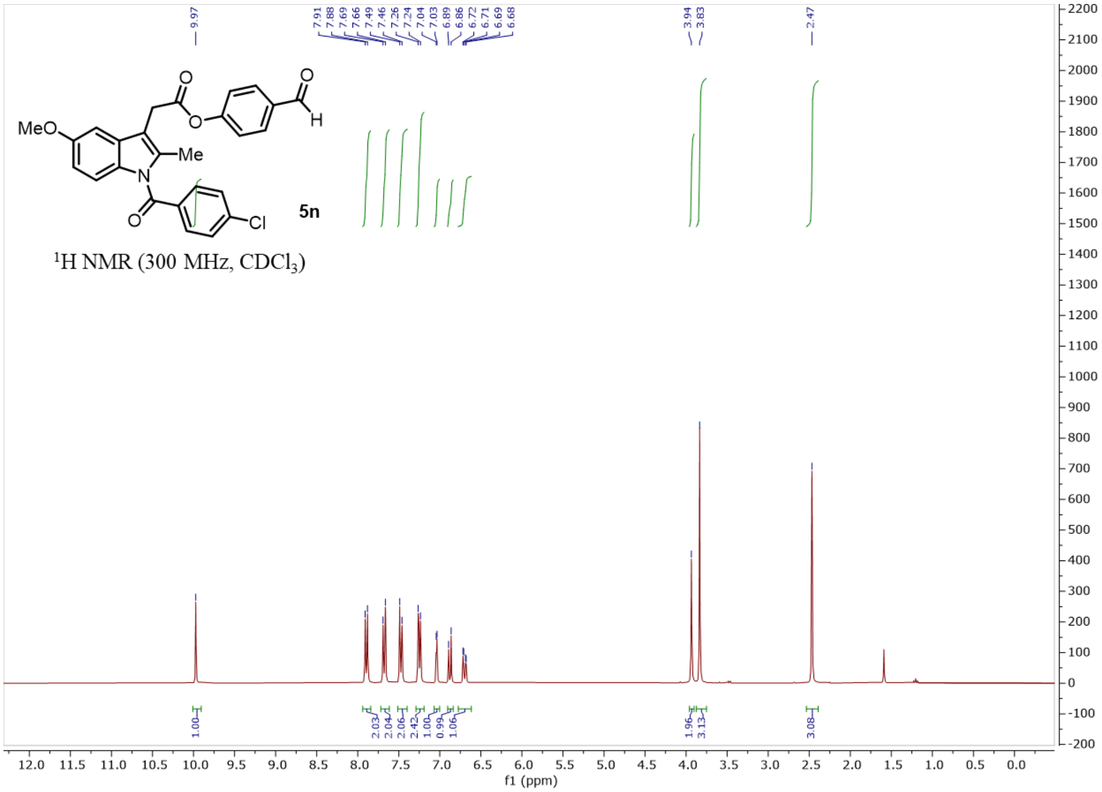

Supplementary Figure 12: ^{1}H NMR (300 MHz, $CDCl_3$) spectrum of compound **5n**.

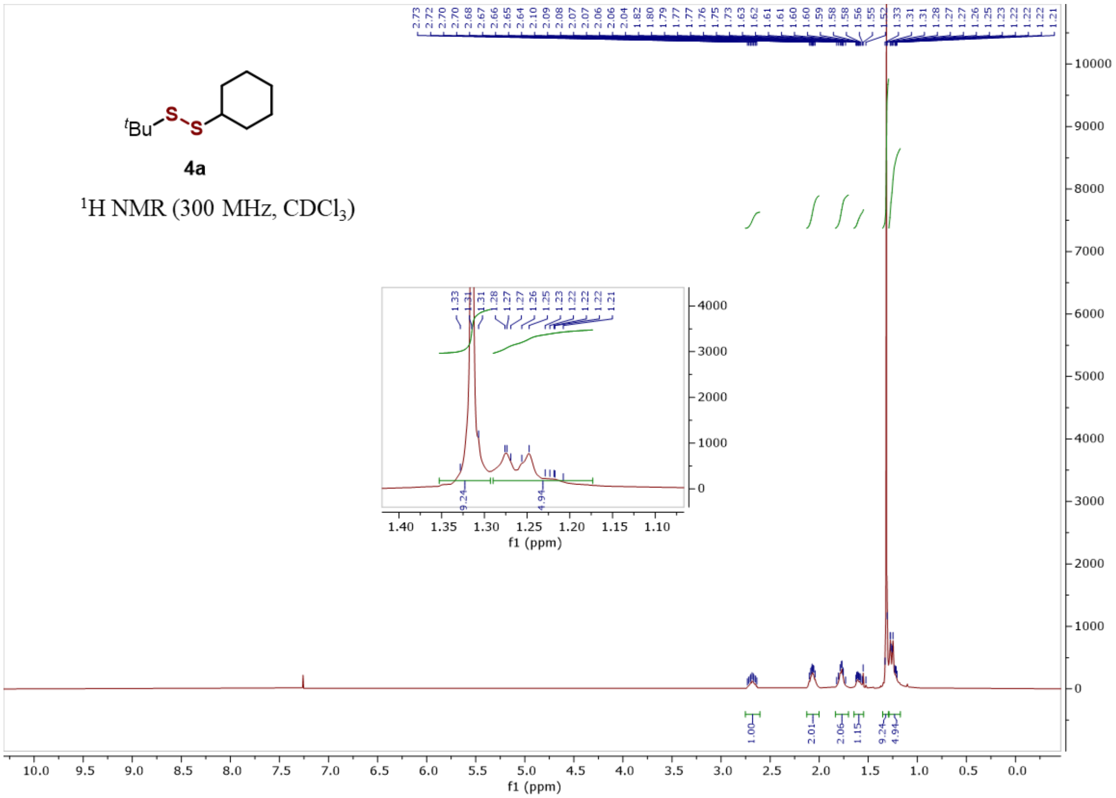

Supplementary Figure 13: ^{1}H NMR (300 MHz, CDCl$_3$) spectrum of compound **4a**.

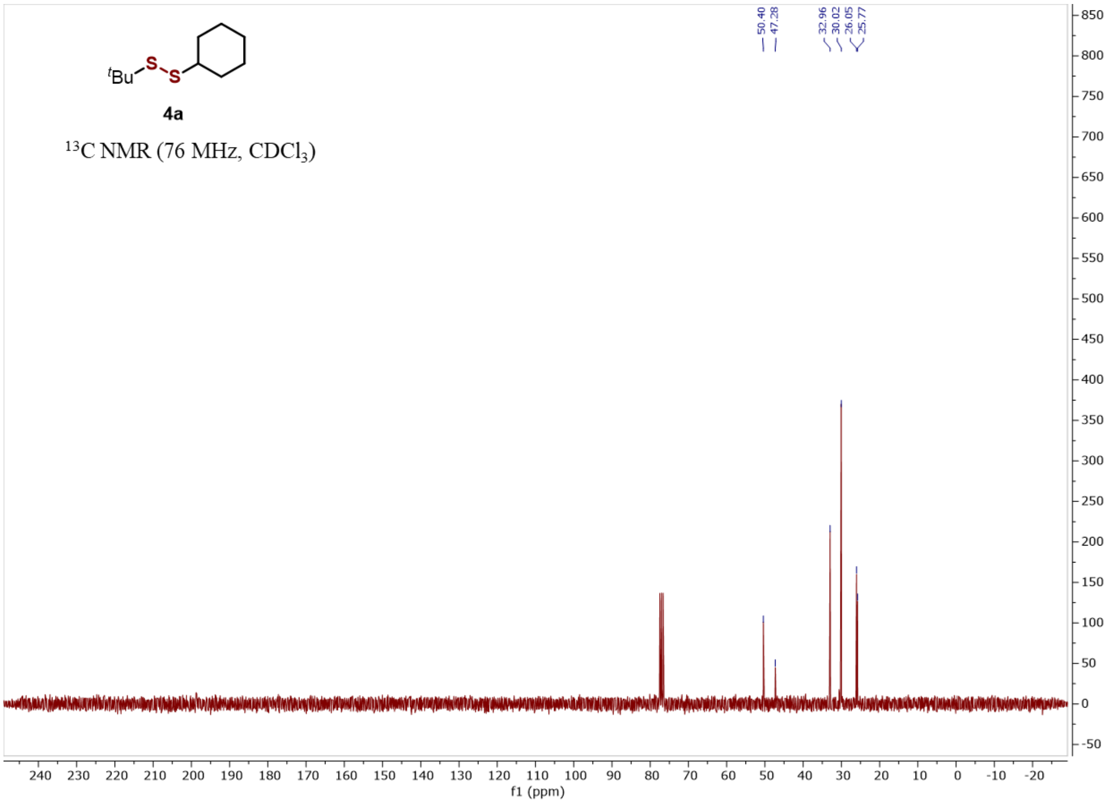

Supplementary Figure 14: ^{13}C NMR (76 MHz, CDCl$_3$) spectrum of compound **4a**.

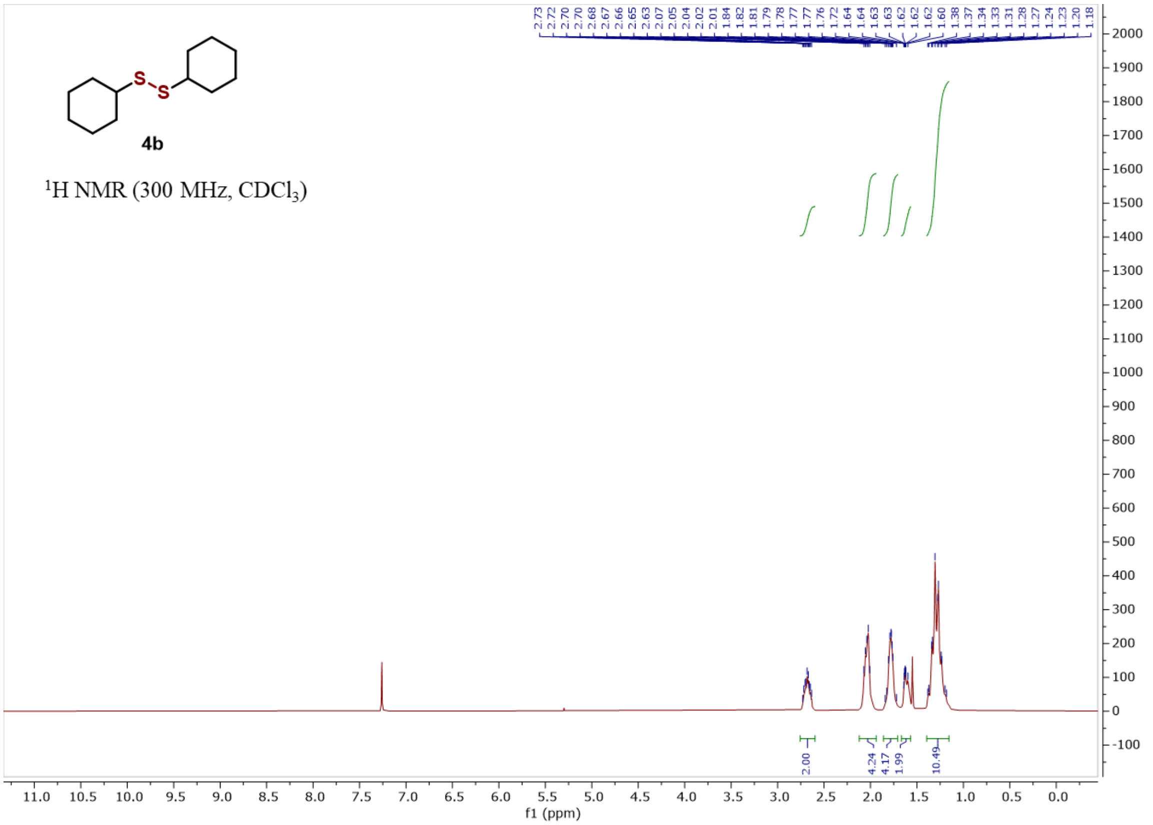

Supplementary Figure 15: ^{1}H NMR (300 MHz, $CDCl_3$) spectrum of compound **4b**.

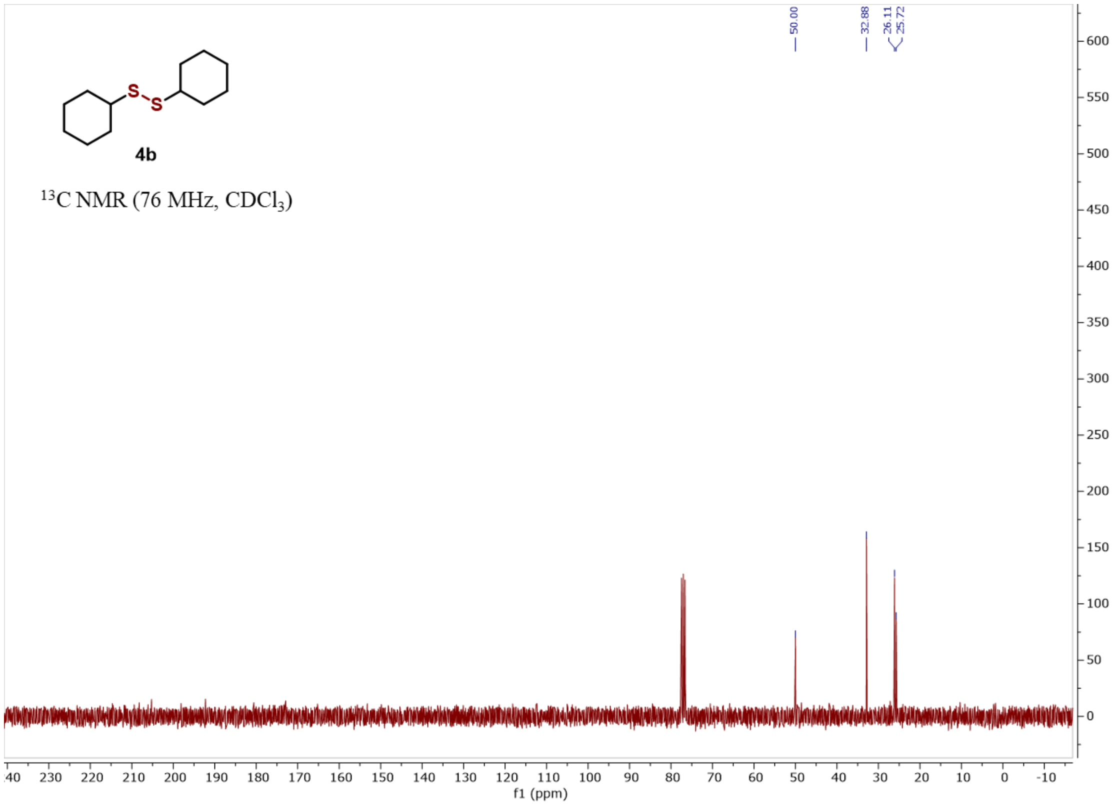

Supplementary Figure 16: ^{13}C NMR (76 MHz, $CDCl_3$) spectrum of compound **4b**.

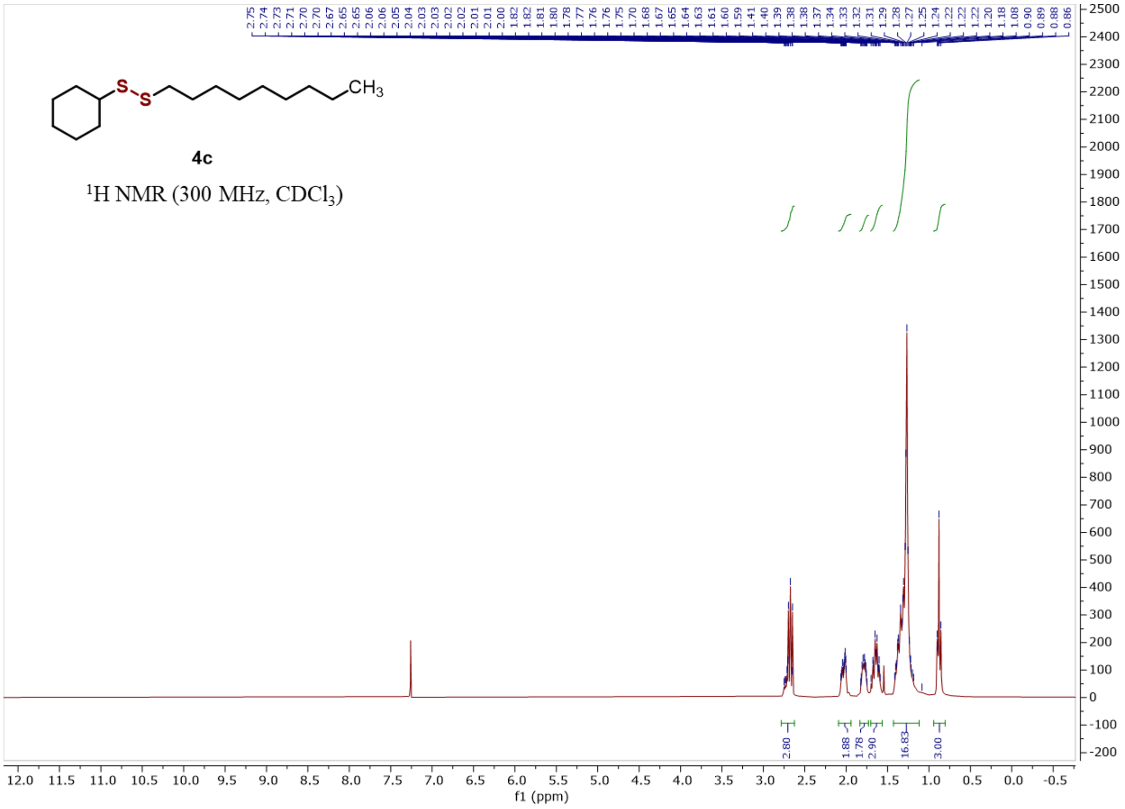

Supplementary Figure 17: ^{1}H NMR (300 MHz, $CDCl_3$) spectrum of compound **4c**.

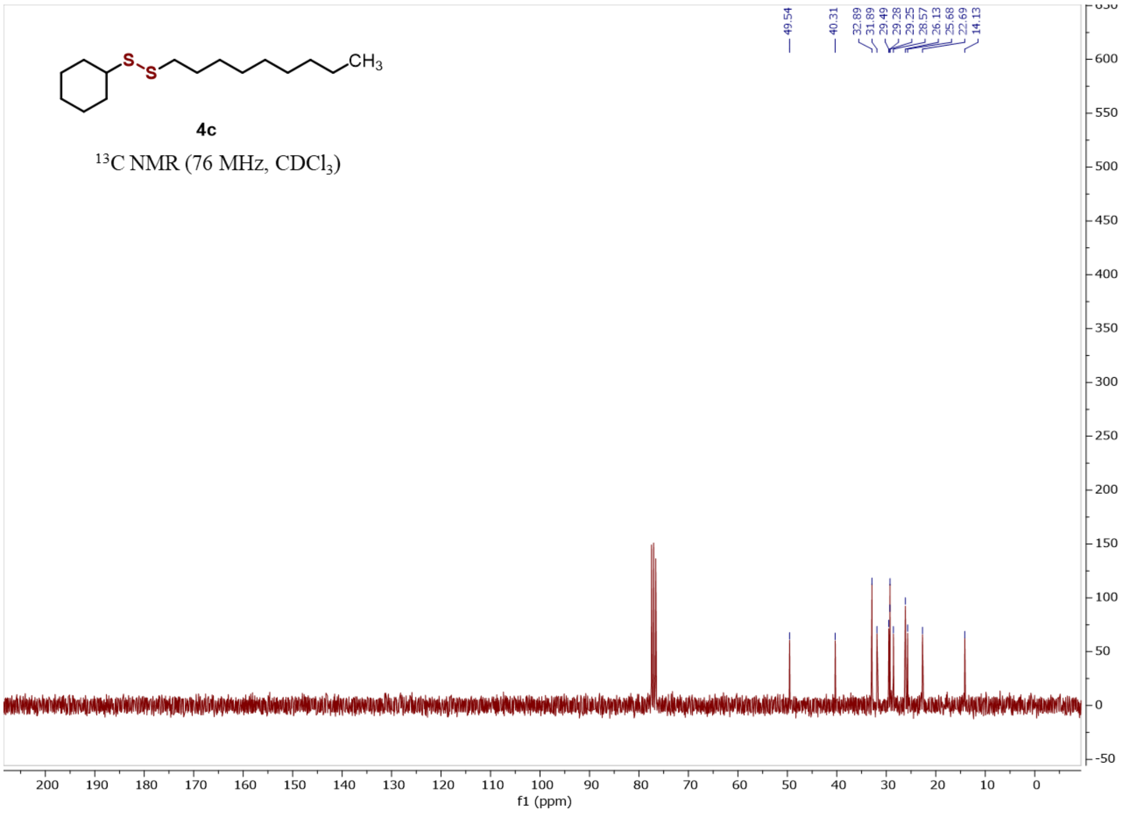

Supplementary Figure 18: ^{13}C NMR (76 MHz, $CDCl_3$) spectrum of compound **4c**.

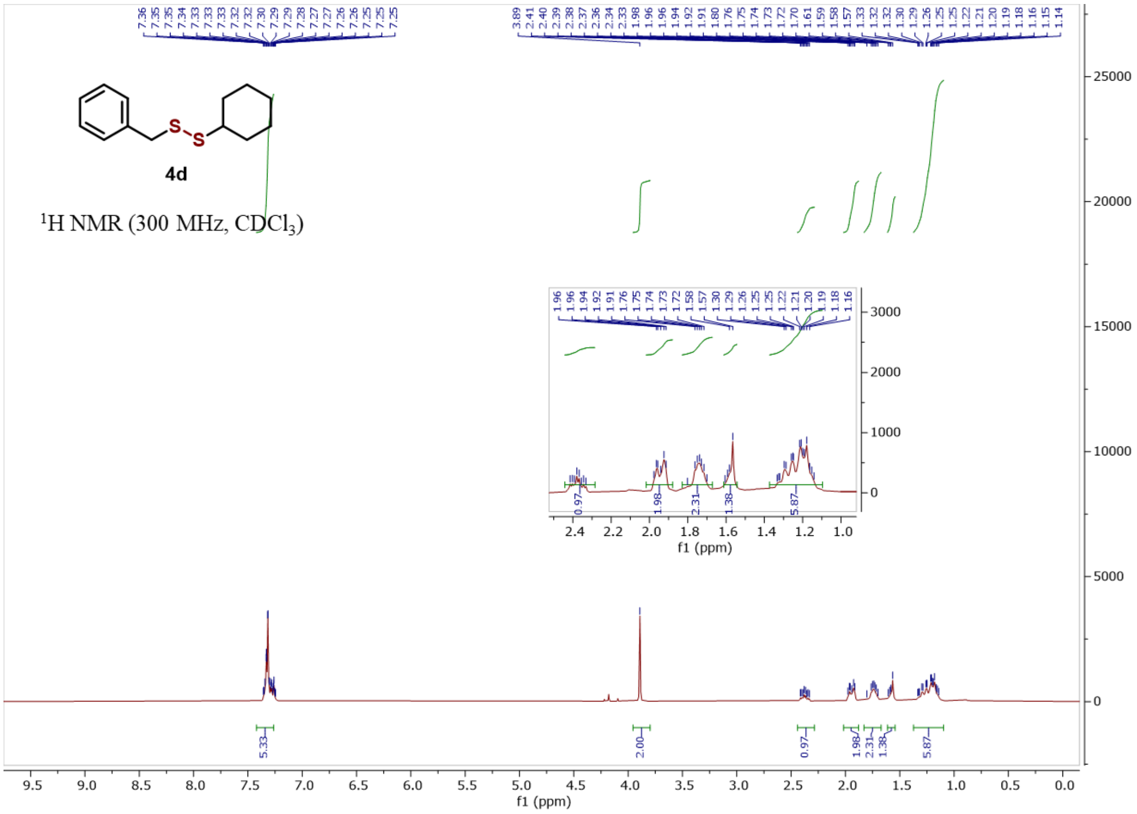

Supplementary Figure 19: ^{1}H NMR (300 MHz, $CDCl_3$) spectrum of compound **4d**.

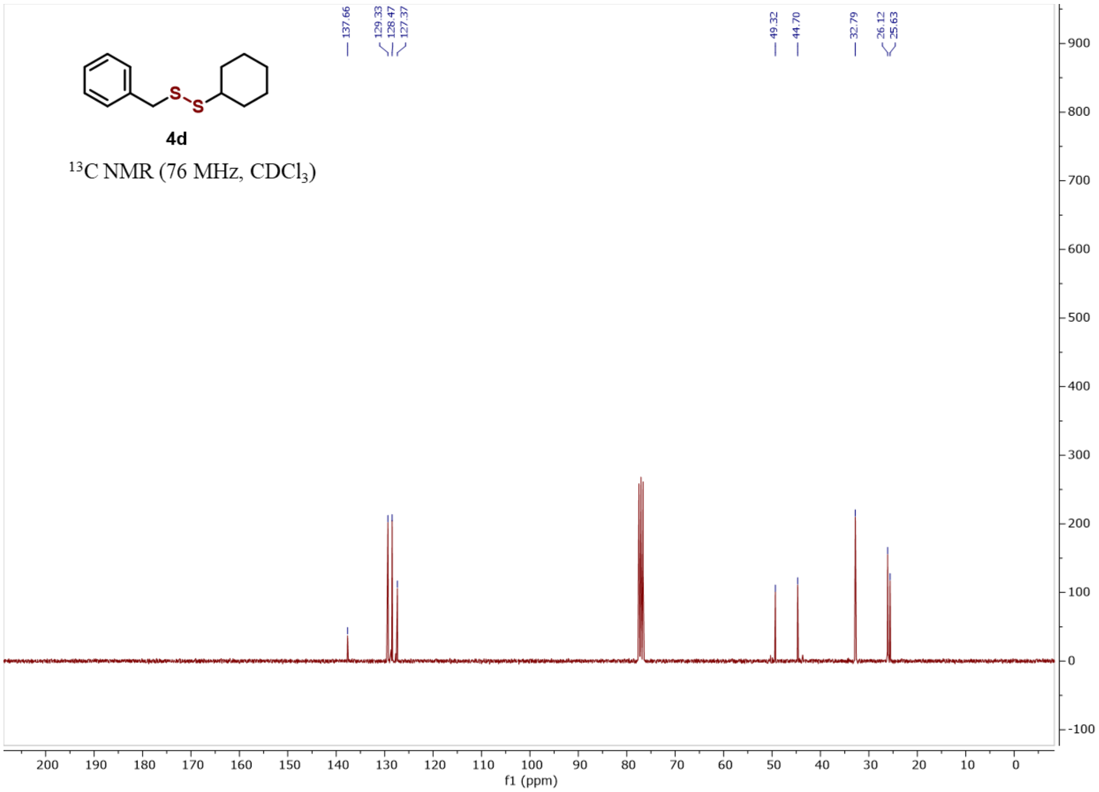

Supplementary Figure 20: ^{13}C NMR (76 MHz, $CDCl_3$) spectrum of compound **4d**.

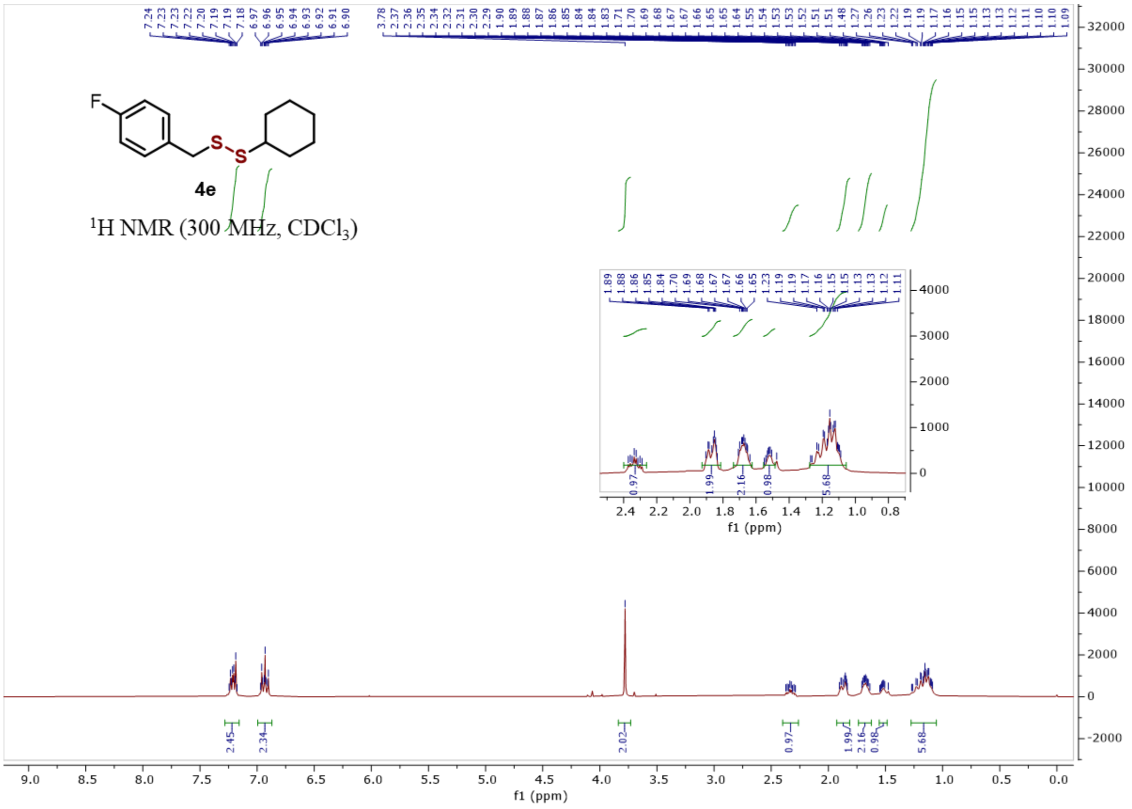

Supplementary Figure 21: ^{1}H NMR (300 MHz, $CDCl_3$) spectrum of compound **4e**.

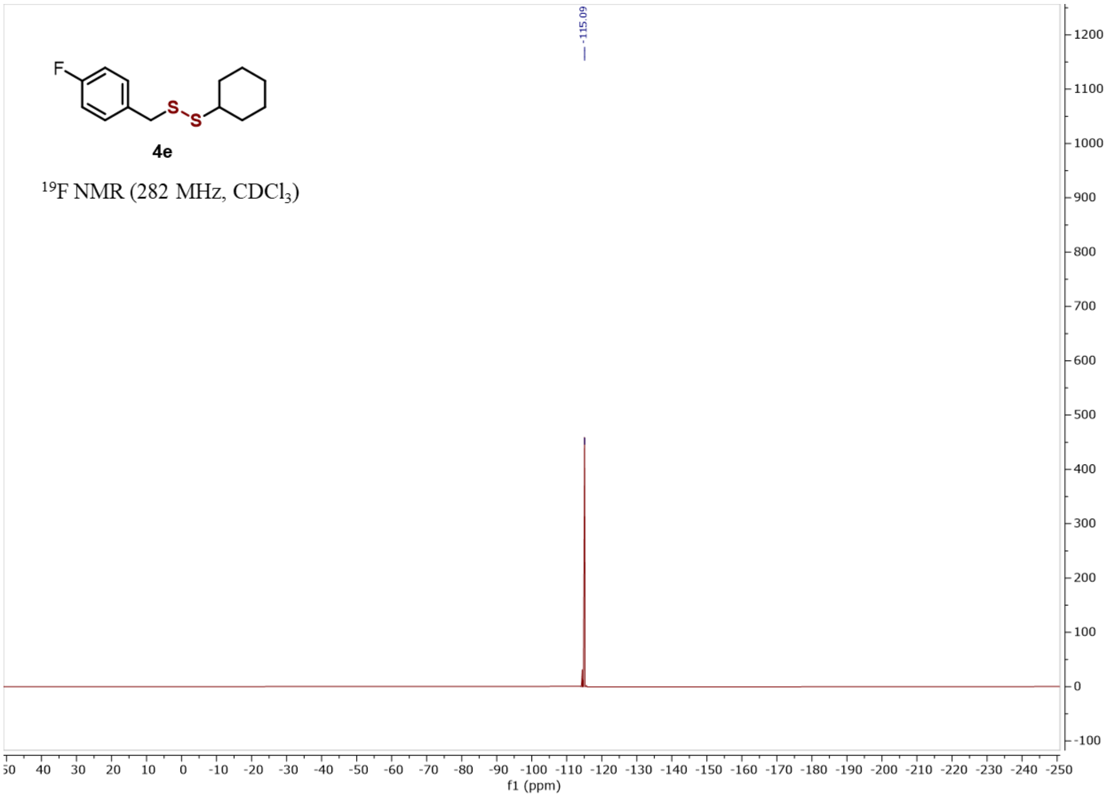

Supplementary Figure 22: ^{19}F NMR (282 MHz, $CDCl_3$) spectrum of compound **4e**.

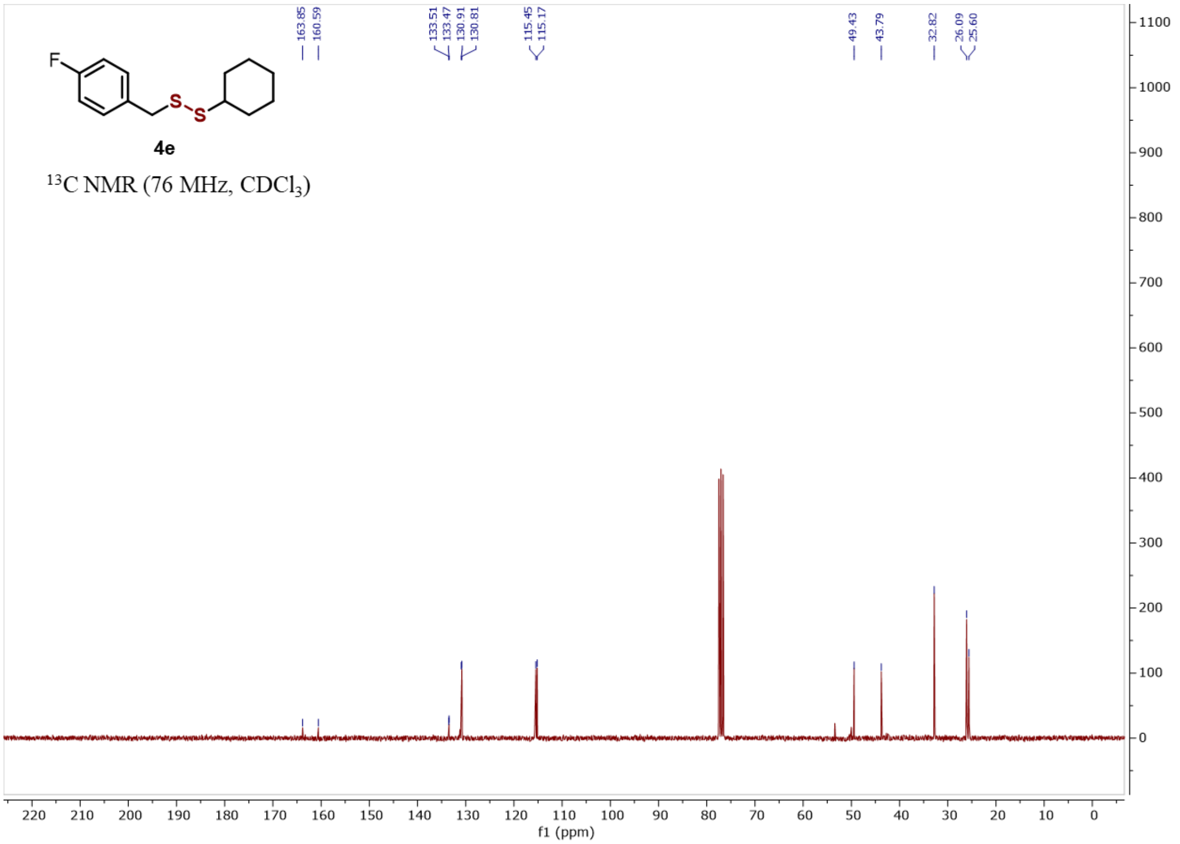

Supplementary Figure 23: ^{13}C NMR (76 MHz, $CDCl_3$) spectrum of compound **4e**.

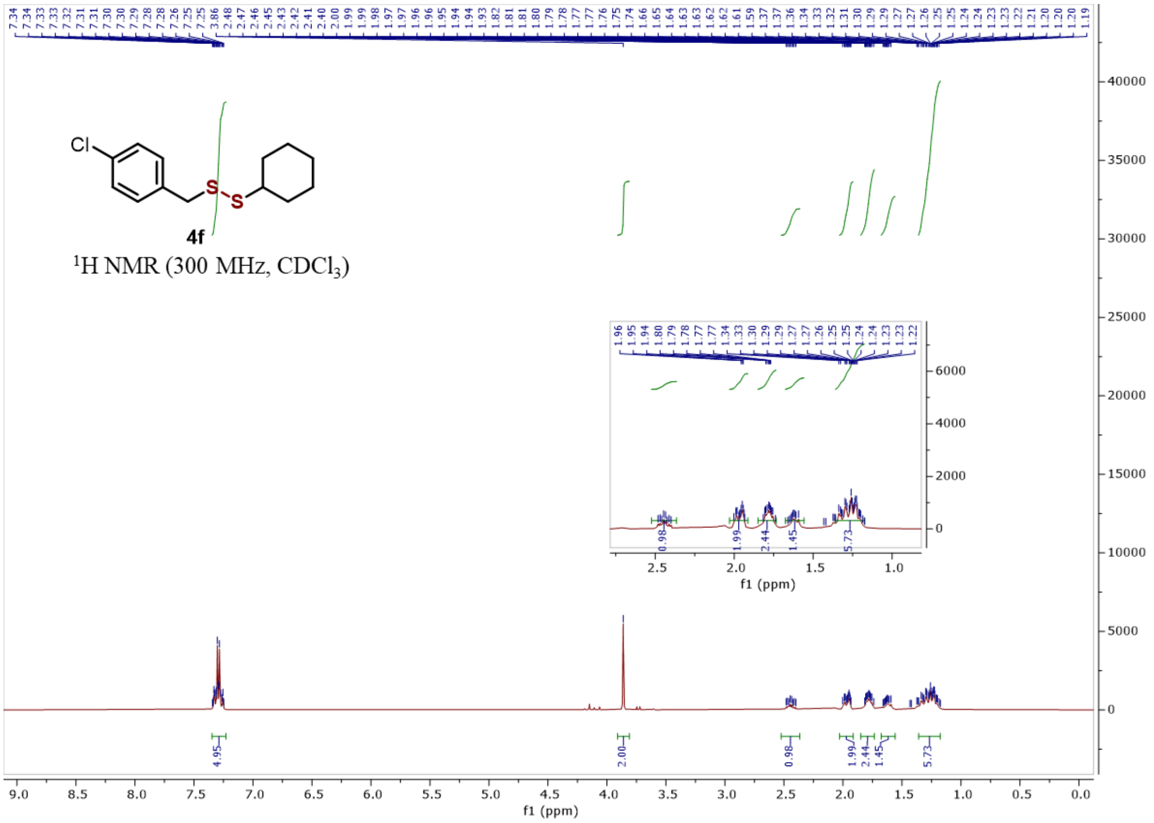

Supplementary Figure 24: ^{1}H NMR (300 MHz, $CDCl_3$) spectrum of compound **4f**.

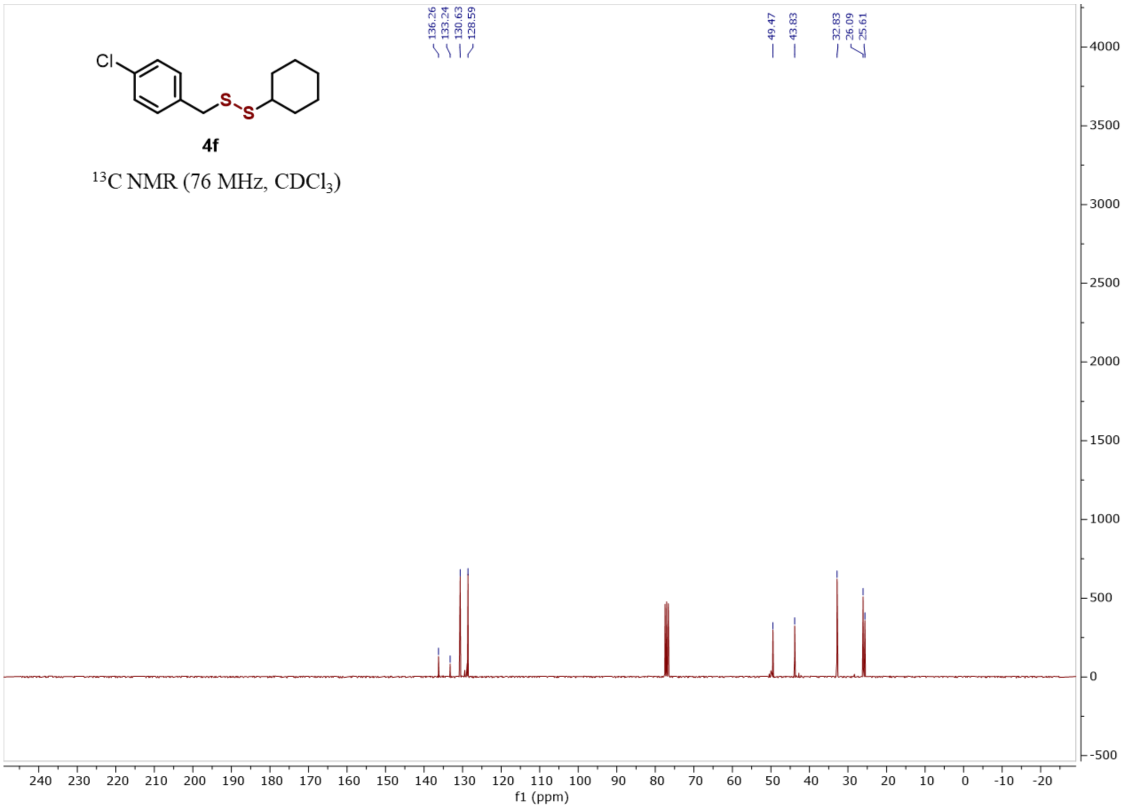

Supplementary Figure 25: ^{13}C NMR (76 MHz, $CDCl_3$) spectrum of compound **4f**.

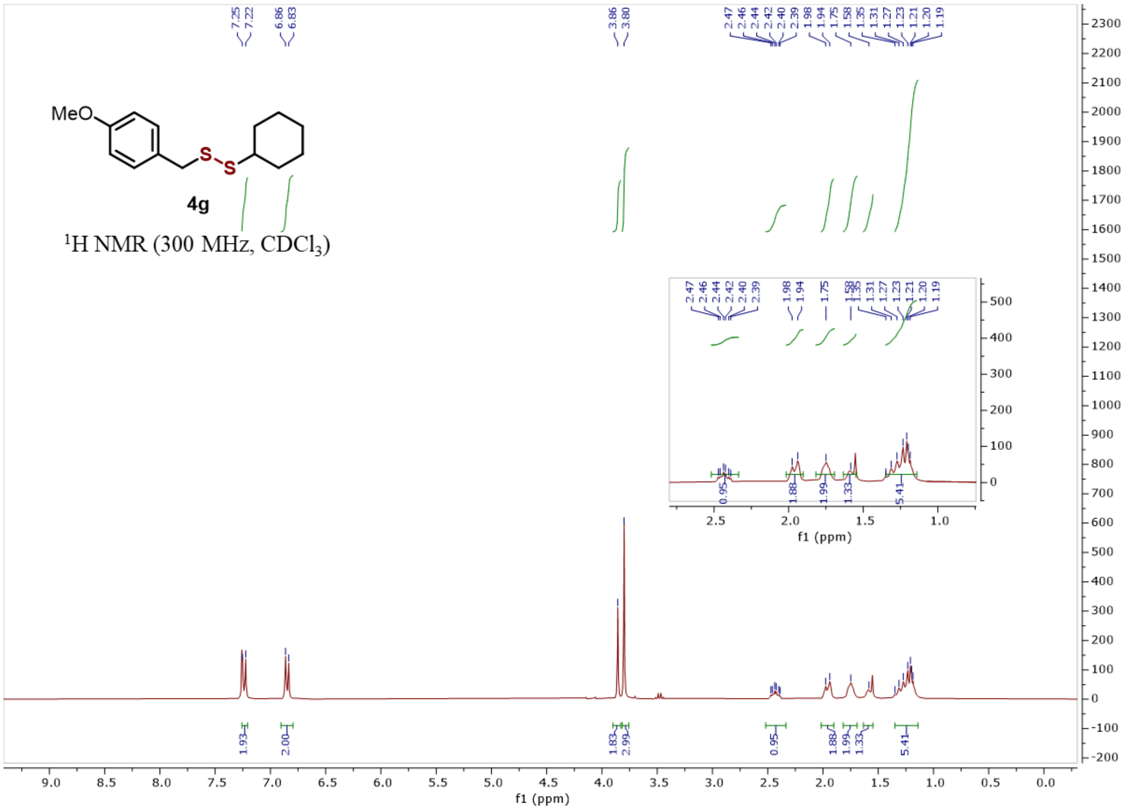

Supplementary Figure 26: ^{1}H NMR (300 MHz, $CDCl_3$) spectrum of compound **4g**.

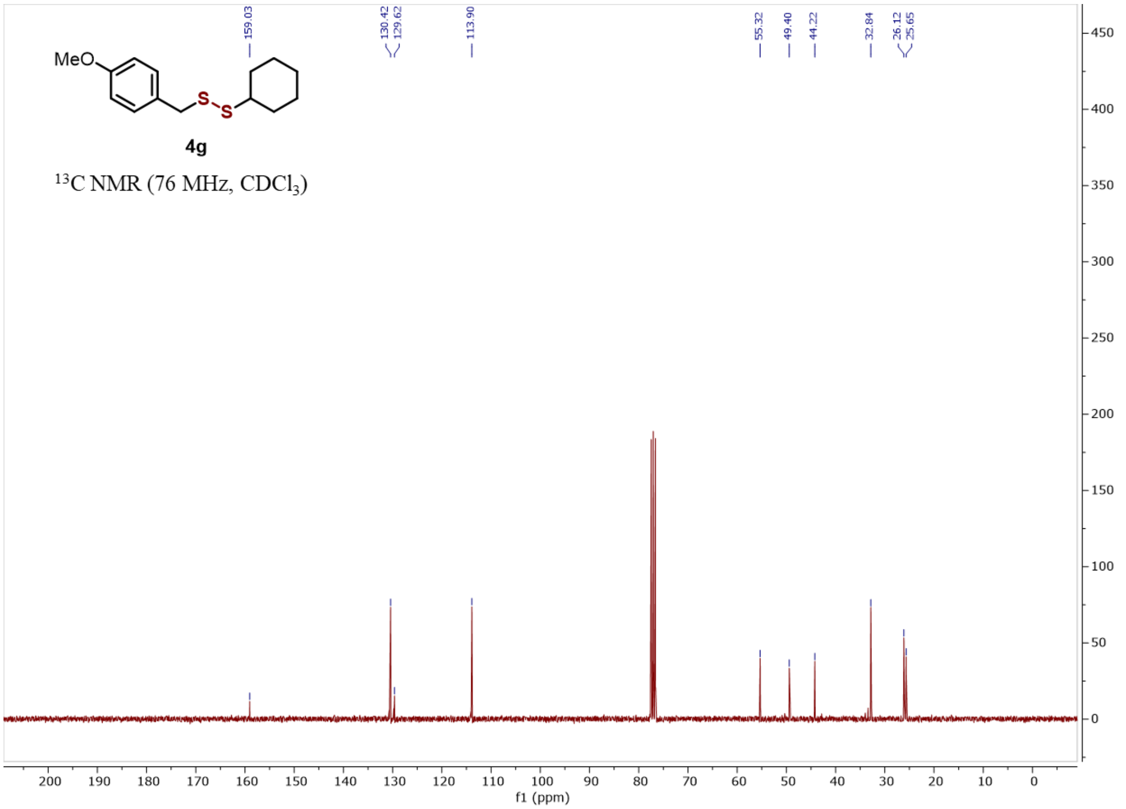

Supplementary Figure 27: ^{13}C NMR (76 MHz, $CDCl_3$) spectrum of compound **4g**.

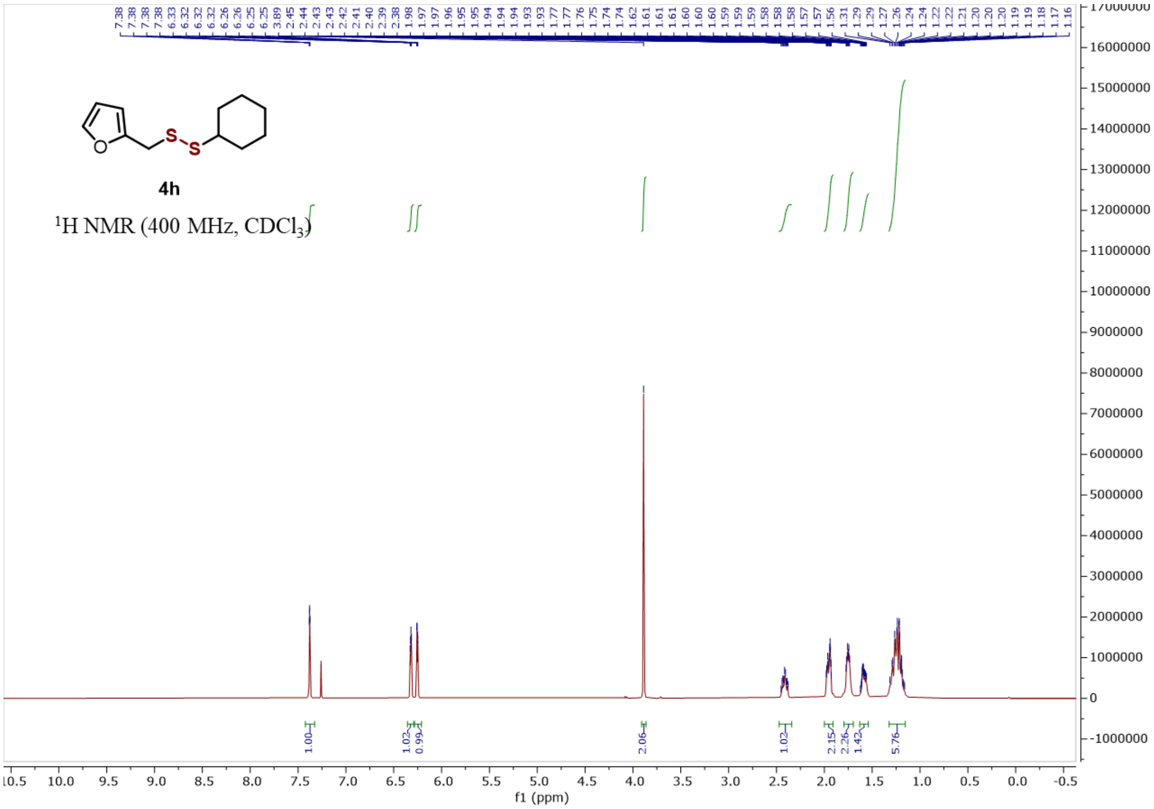

Supplementary Figure 28: ^{1}H NMR (400 MHz, CDCl$_3$) spectrum of compound **4h**.

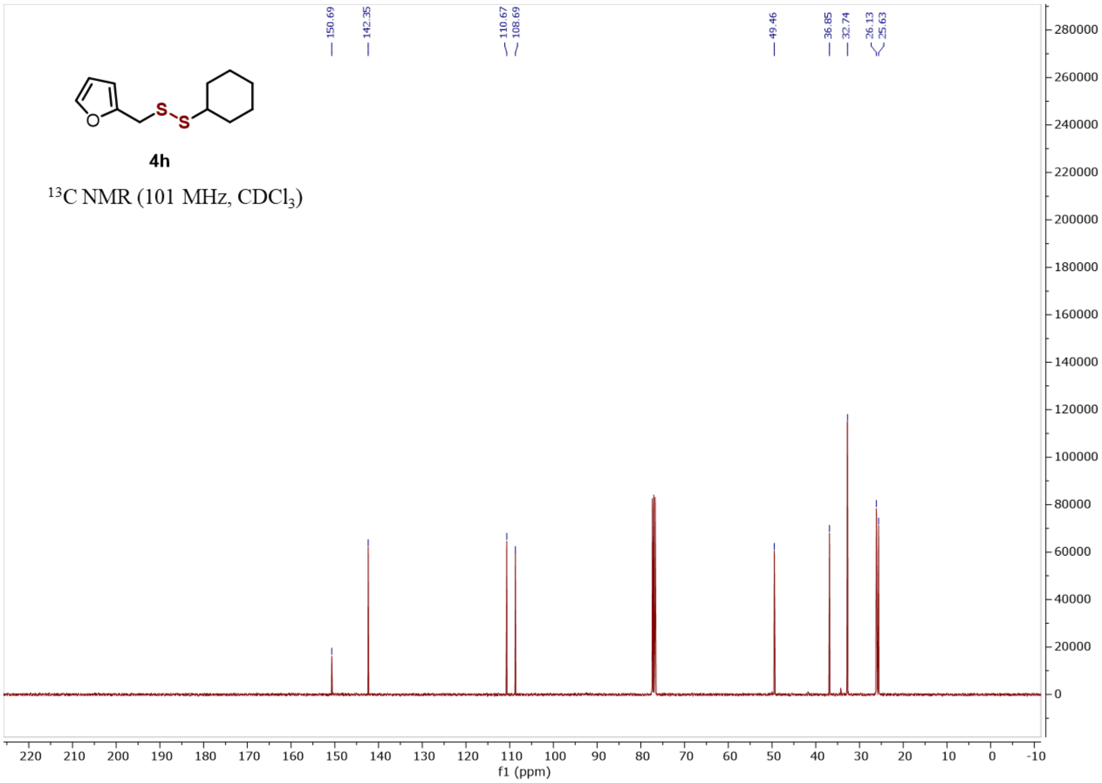

Supplementary Figure 29: ^{13}C NMR (101 MHz, CDCl$_3$) spectrum of compound **4h**.

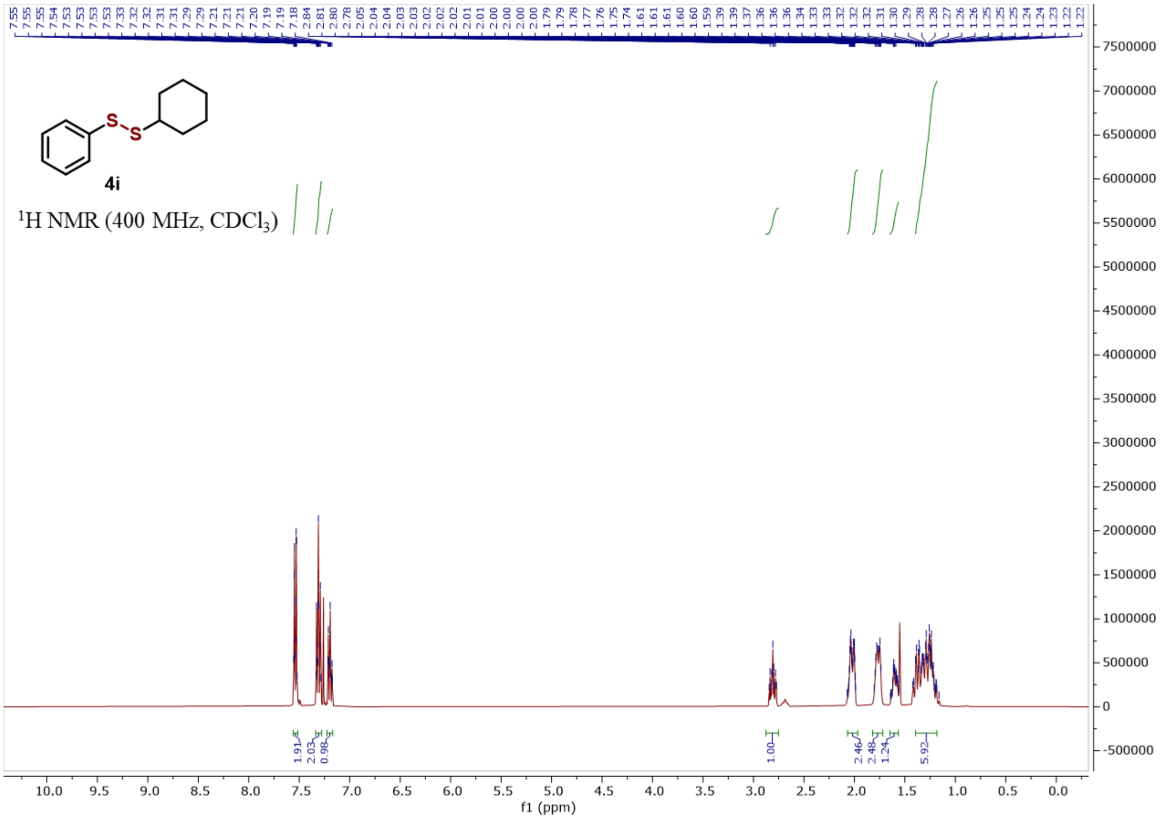

Supplementary Figure 30: ¹H NMR (400 MHz, CDCl₃) spectrum of compound **4i**.

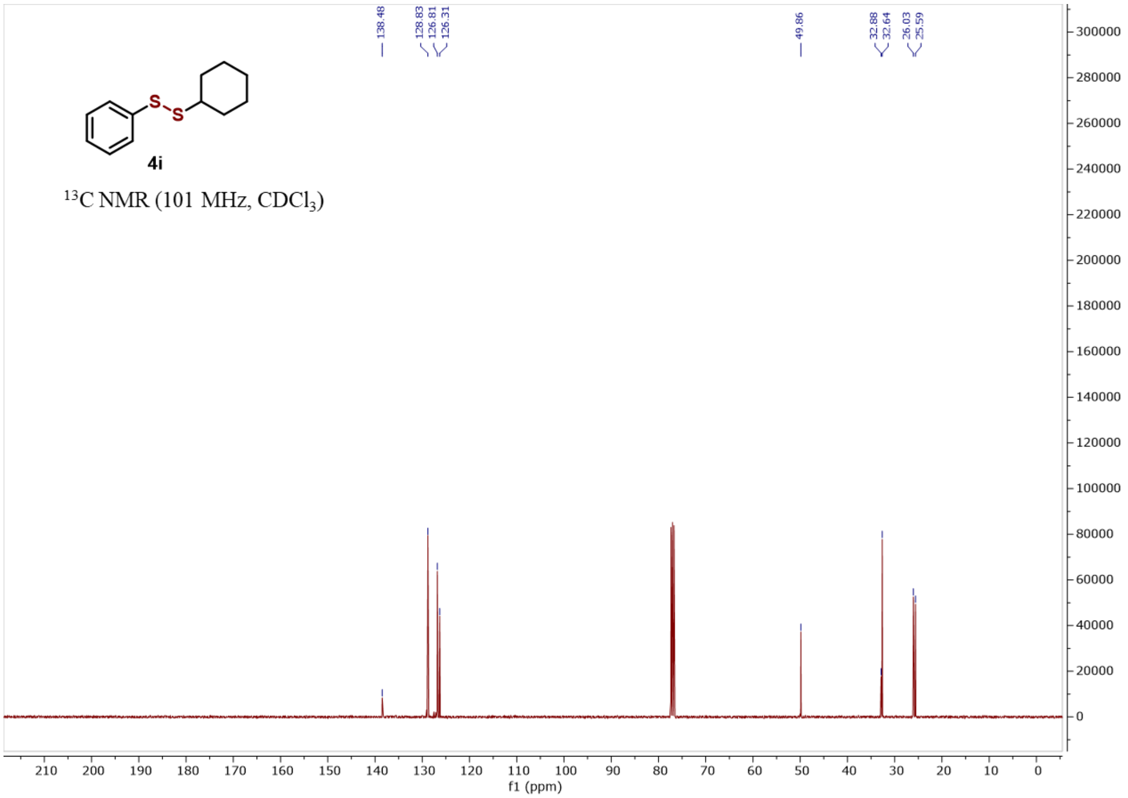

Supplementary Figure 31: ¹³C NMR (101 MHz, CDCl₃) spectrum of compound **4i**.

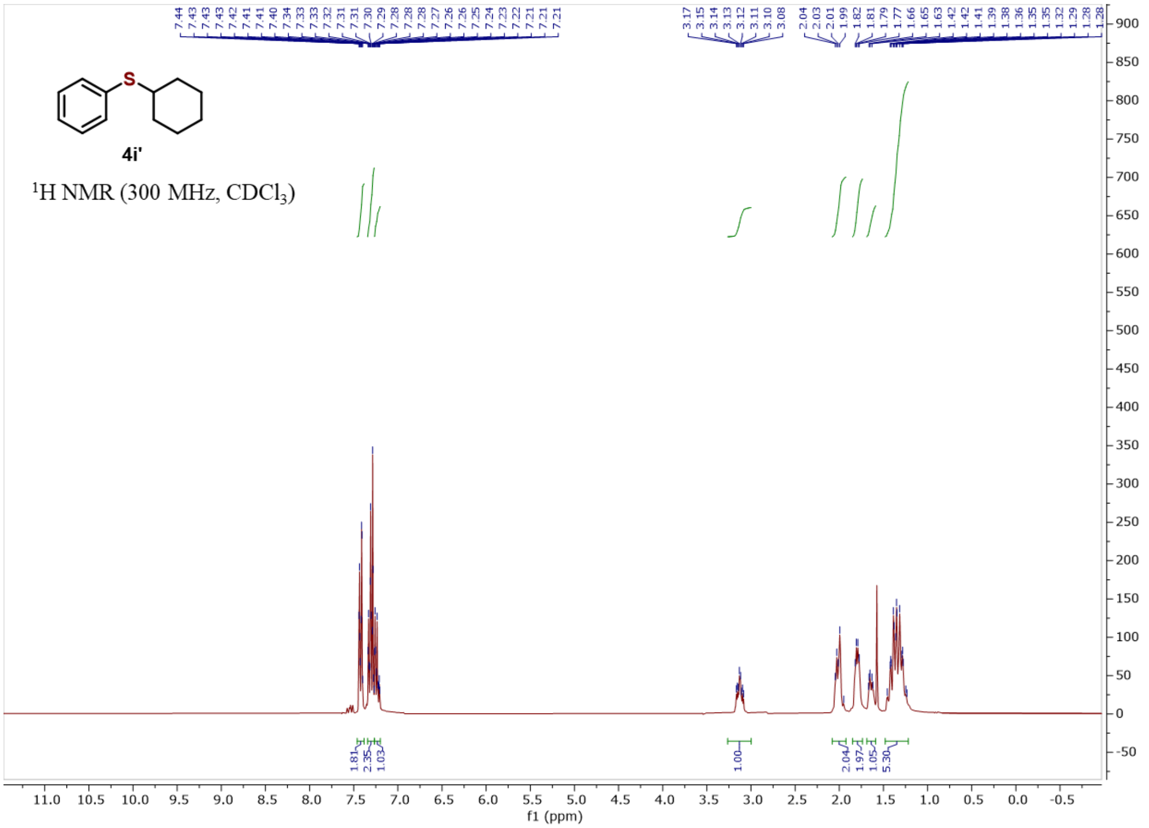

Supplementary Figure 32: ^{1}H NMR (300 MHz, $CDCl_3$) spectrum of compound **4i'**.

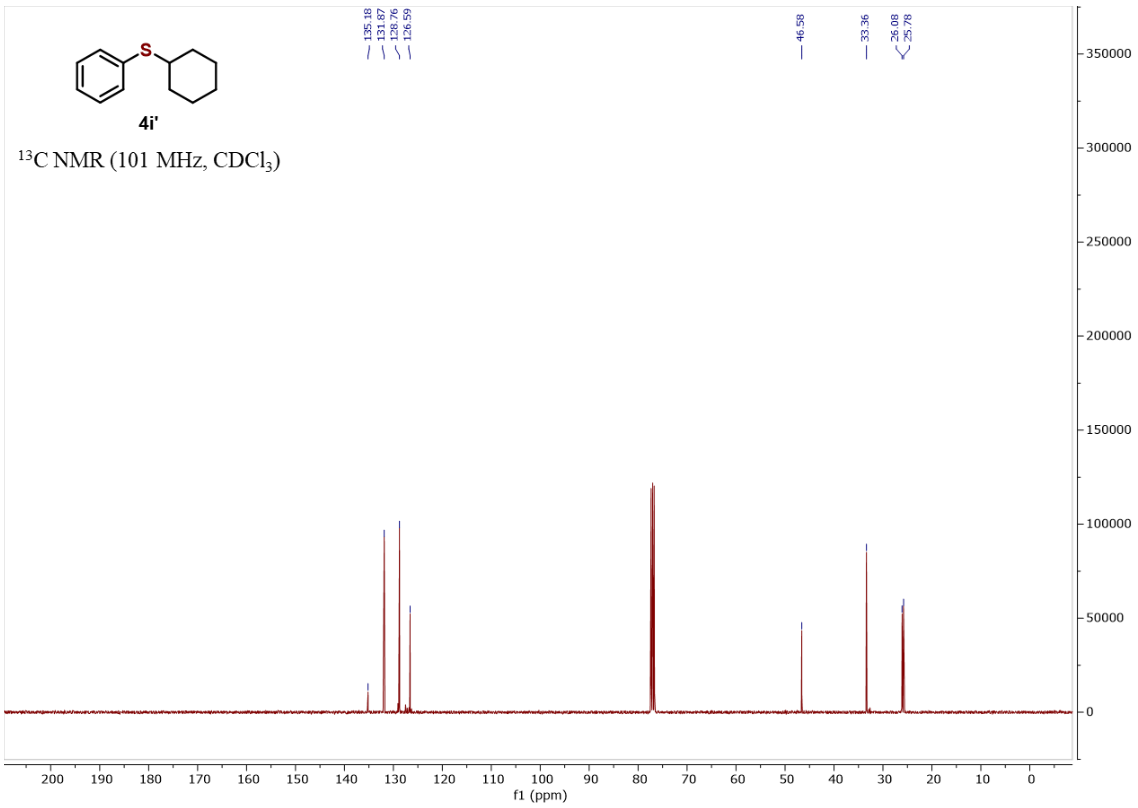

Supplementary Figure 33: ^{13}C NMR (76 MHz, $CDCl_3$) spectrum of compound **4i'**.

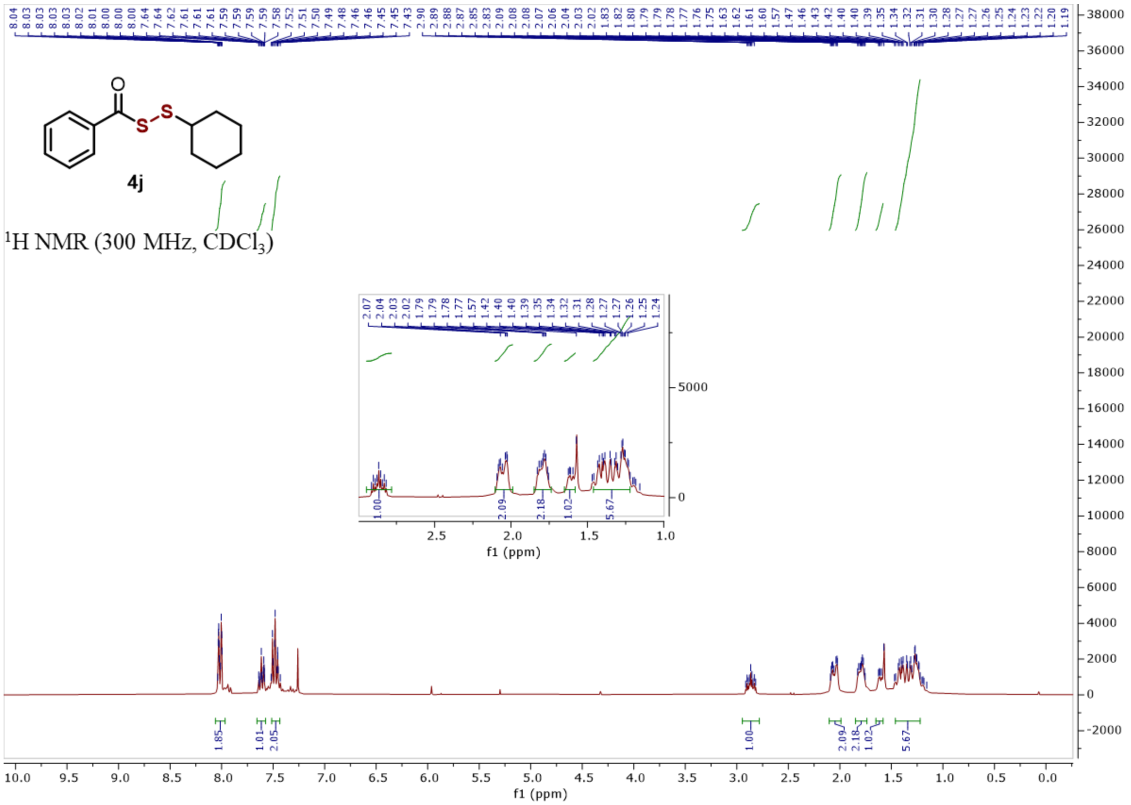

Supplementary Figure 34: ^{1}H NMR (300 MHz, CDCl$_3$) spectrum of compound **4j**.

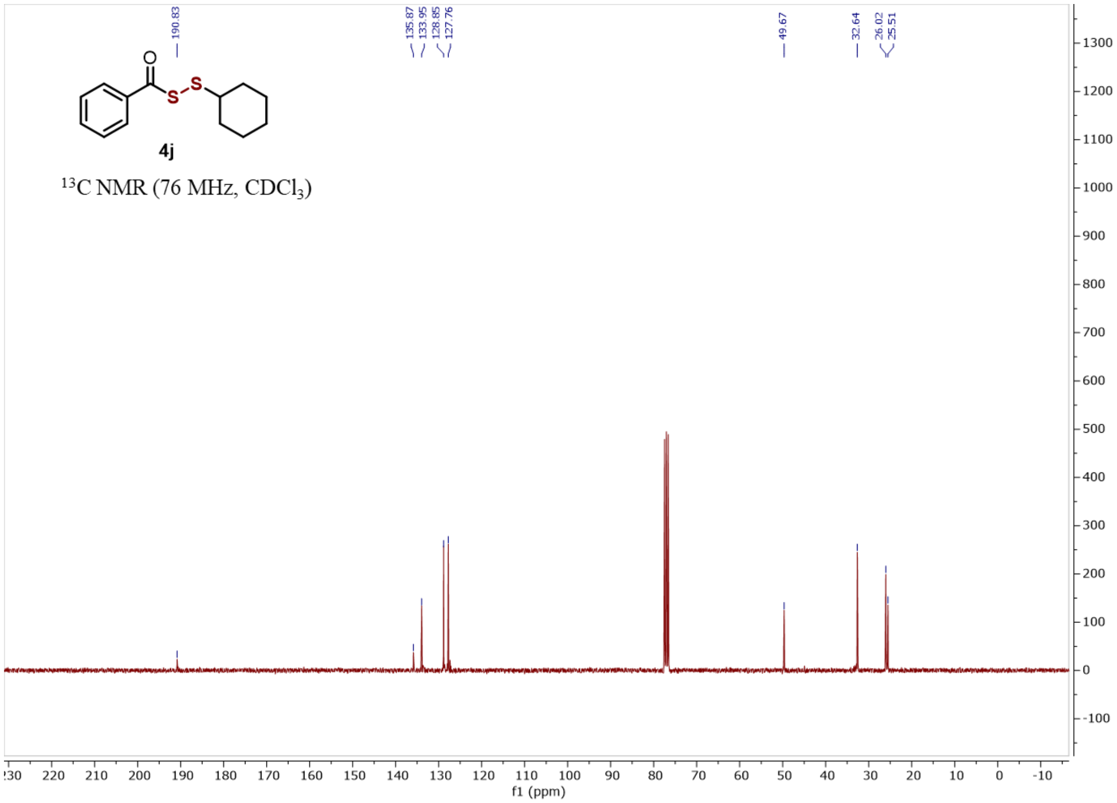

Supplementary Figure 35: ^{13}C NMR (76 MHz, CDCl$_3$) spectrum of compound **4j**.

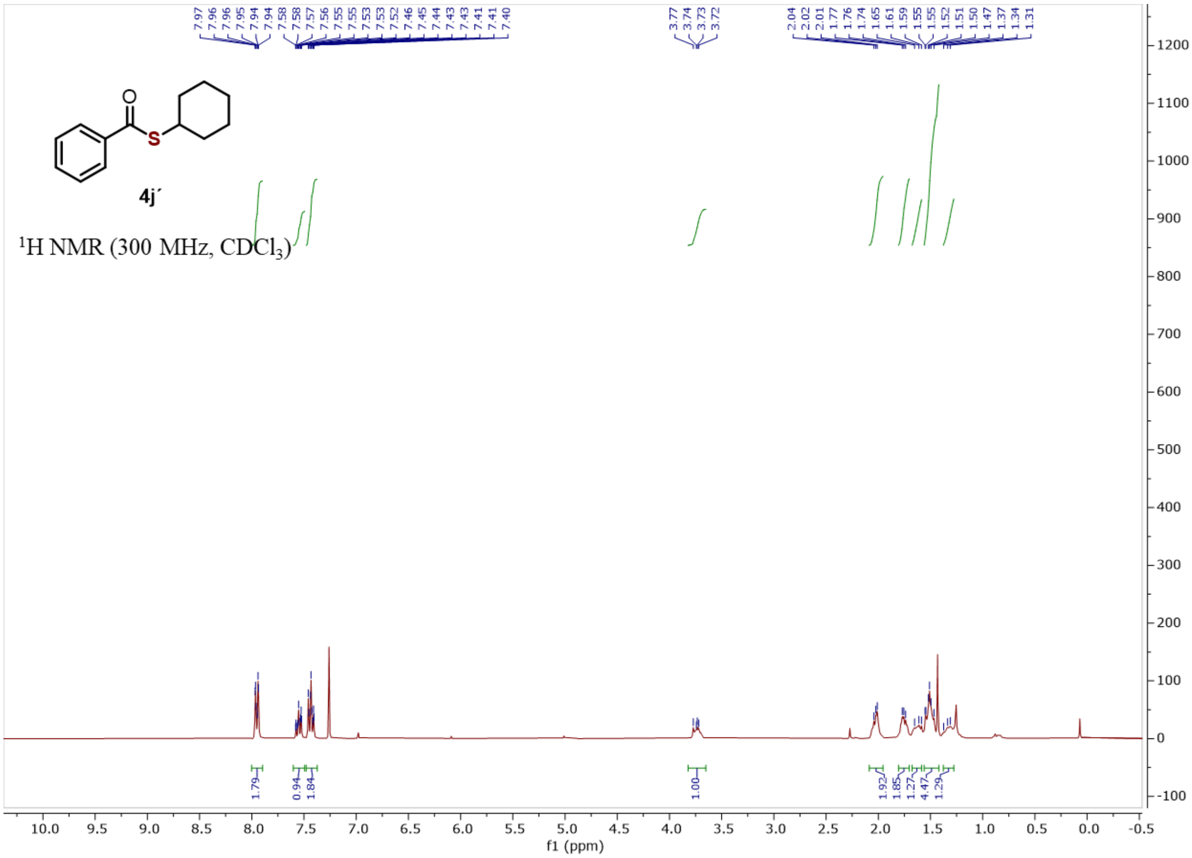

Supplementary Figure 36: ¹H NMR (300 MHz, $CDCl_3$) spectrum of compound **4j'**.

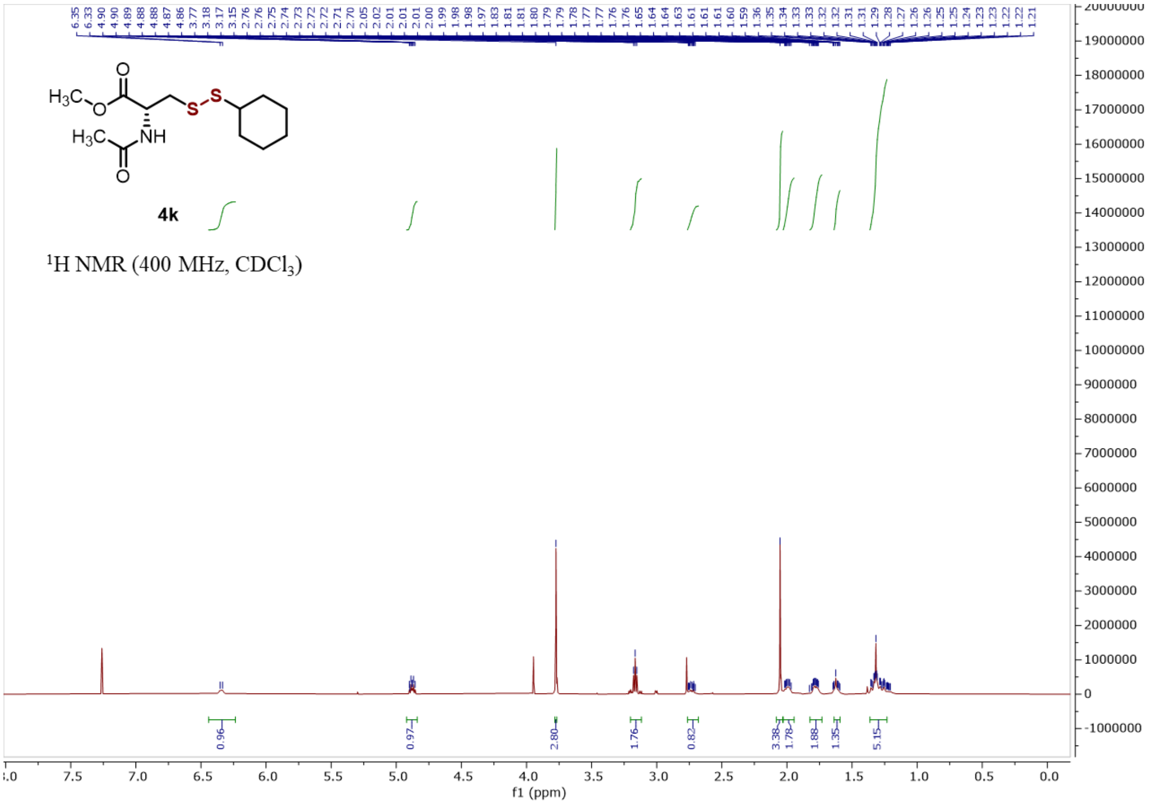

Supplementary Figure 37: ^{1}H NMR (400 MHz, $CDCl_3$) spectrum of compound **4k**.

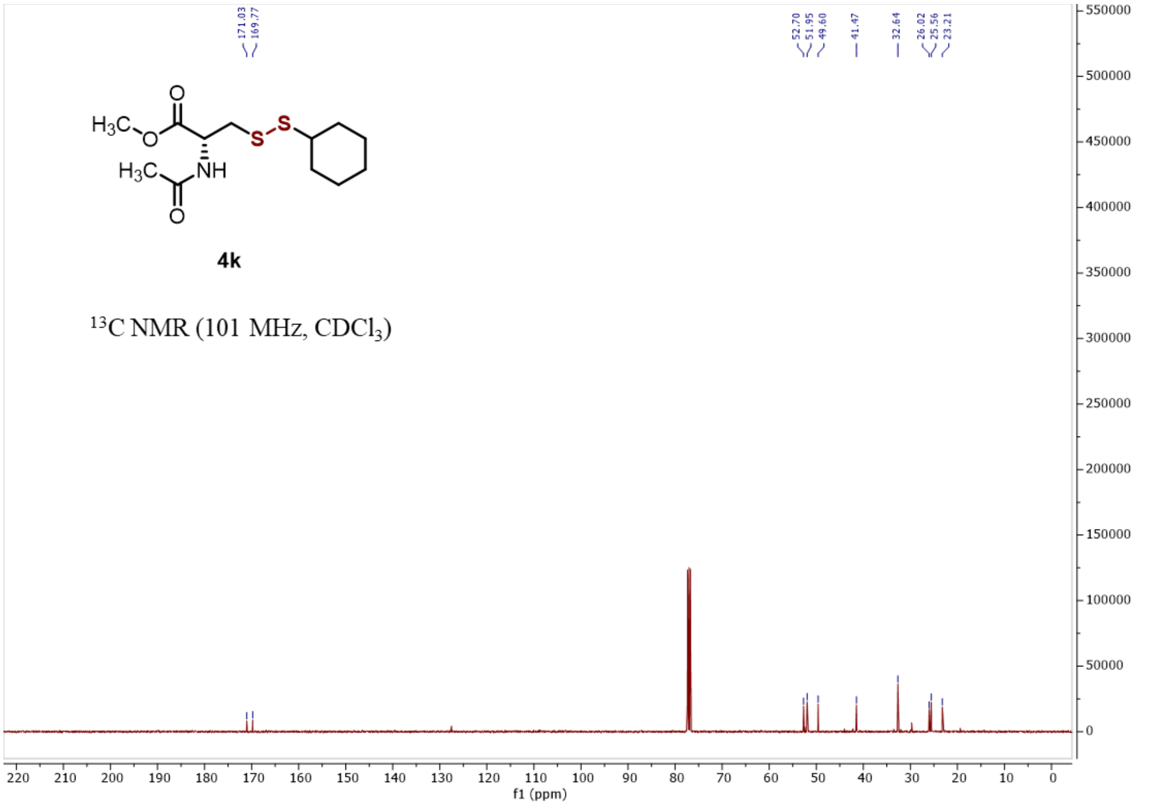

Supplementary Figure 38: ^{13}C NMR (101 MHz, $CDCl_3$) spectrum of compound **4k**.

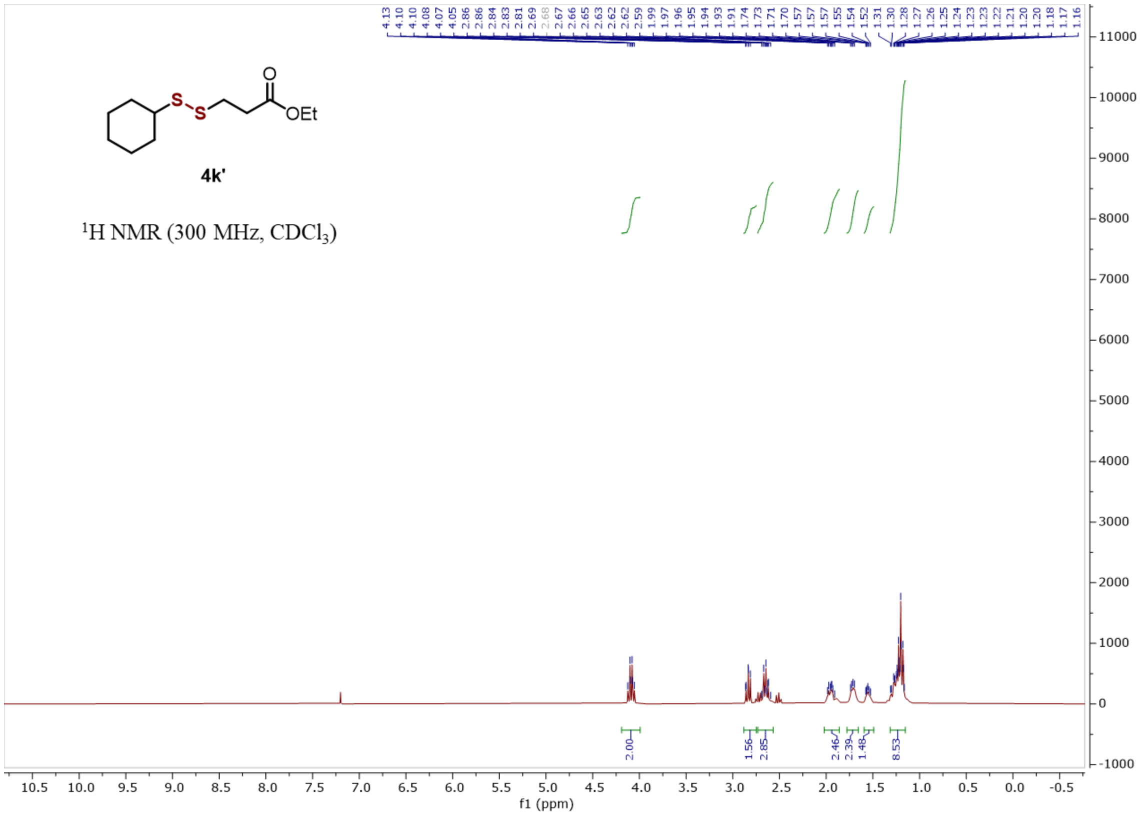

Supplementary Figure 39: ^{1}H NMR (300 MHz, $CDCl_3$) spectrum of compound **4k'**.

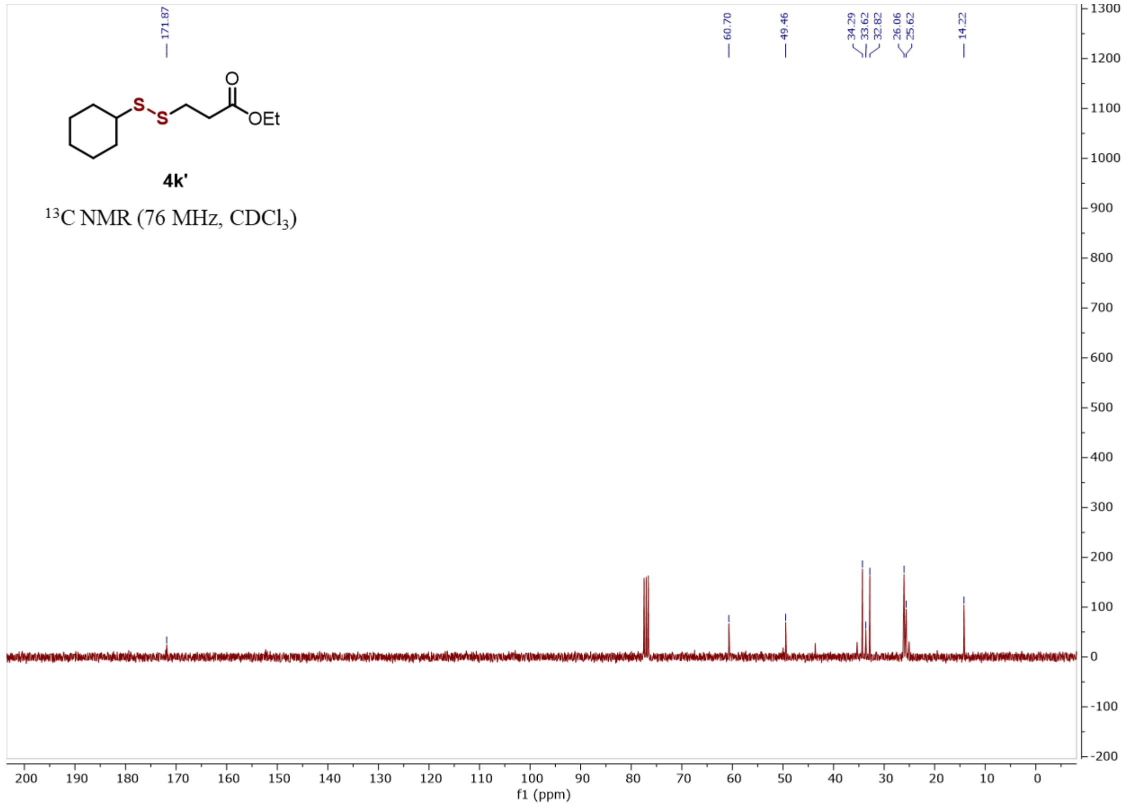

Supplementary Figure 40: ^{13}C NMR (76 MHz, $CDCl_3$) spectrum of compound **4k'**.

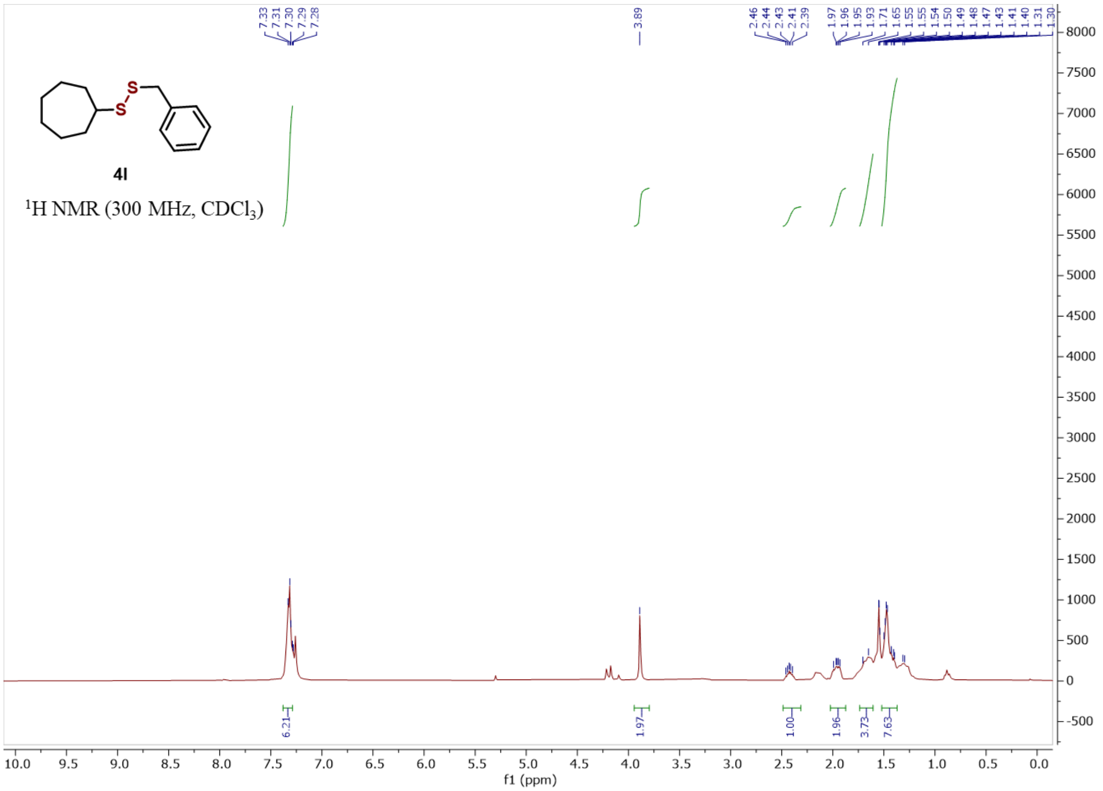

Supplementary Figure 41: ^{1}H NMR (300 MHz, $CDCl_3$) spectrum of compound **4l**.

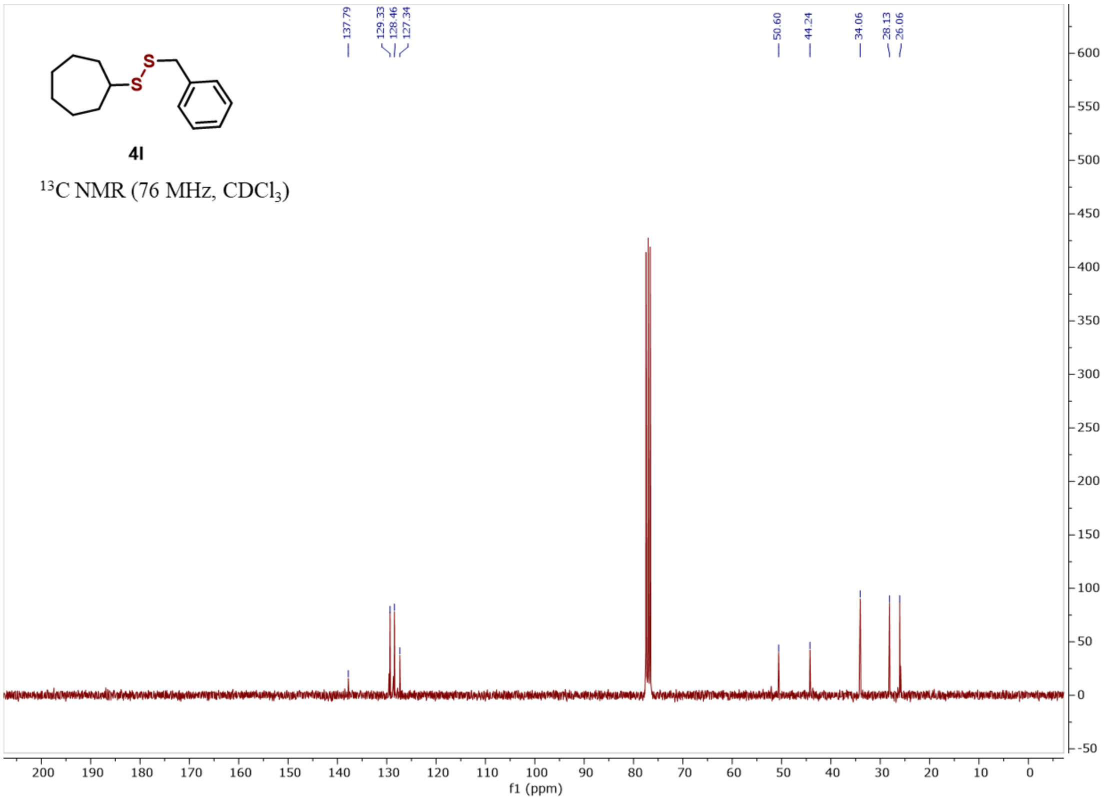

Supplementary Figure 42: ^{13}C NMR (76 MHz, $CDCl_3$) spectrum of compound **4l**.

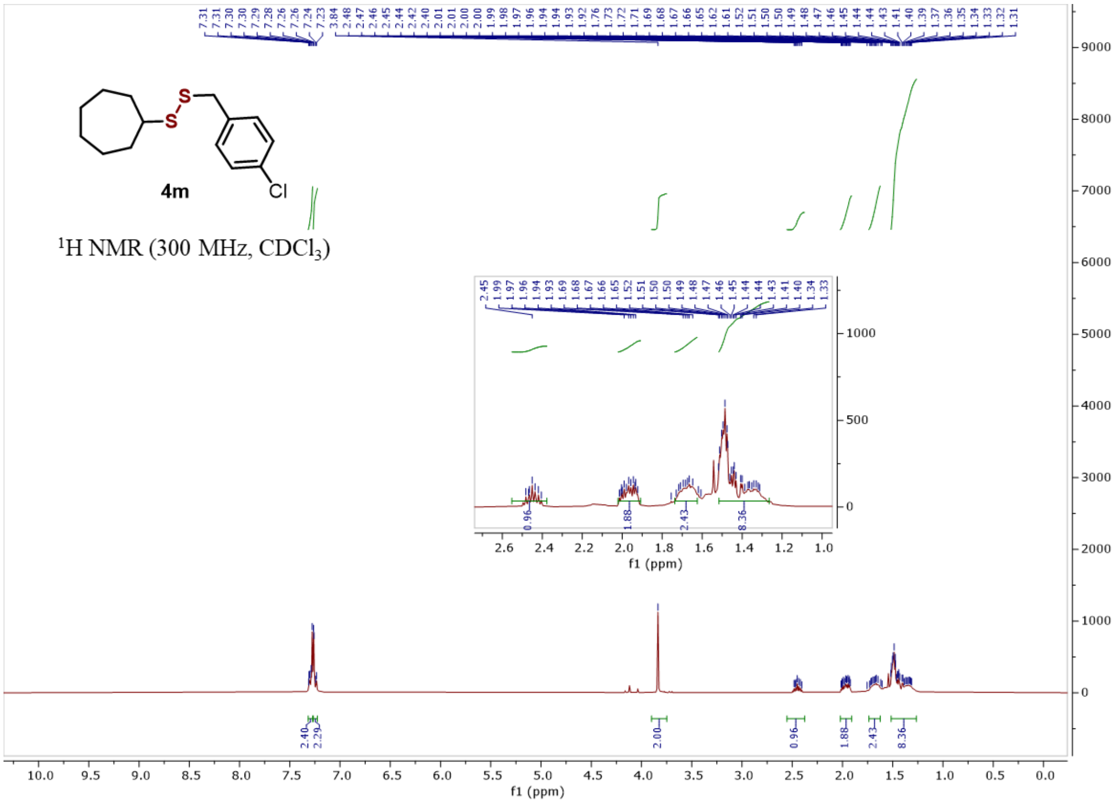

Supplementary Figure 43: ^{1}H NMR (300 MHz, $CDCl_3$) spectrum of compound **4m**.

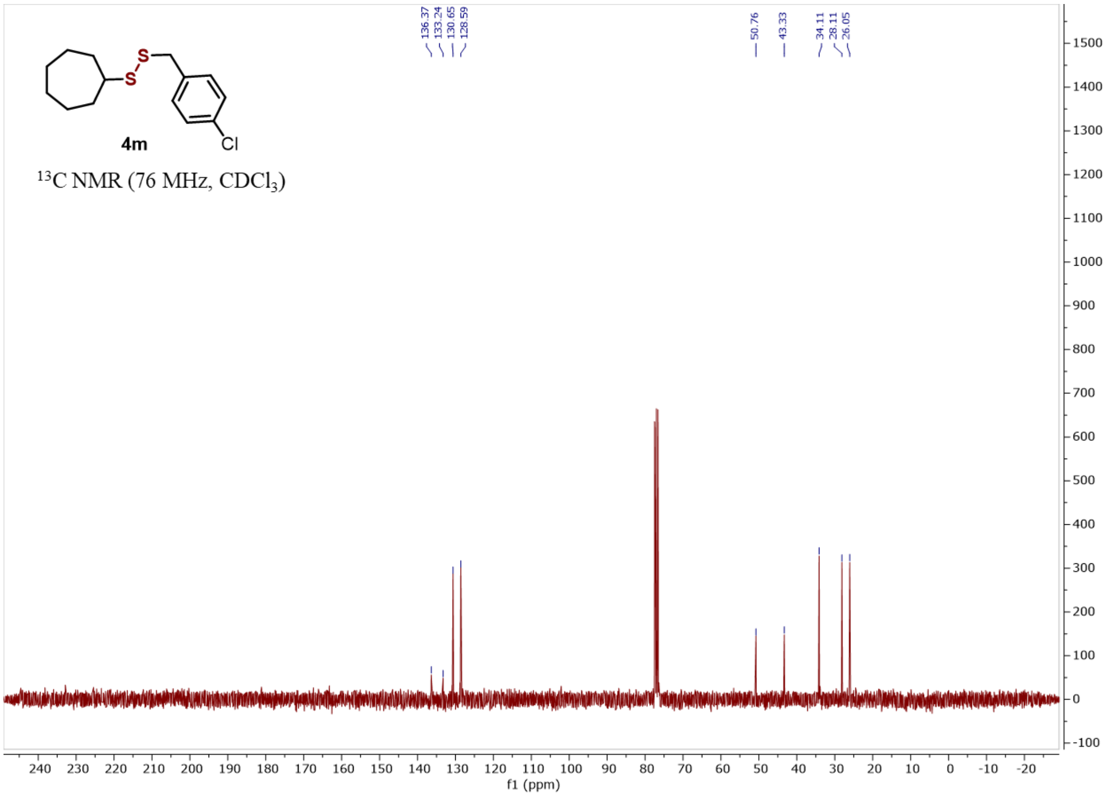

Supplementary Figure 44: ^{13}C NMR (76 MHz, $CDCl_3$) spectrum of compound **4m**.

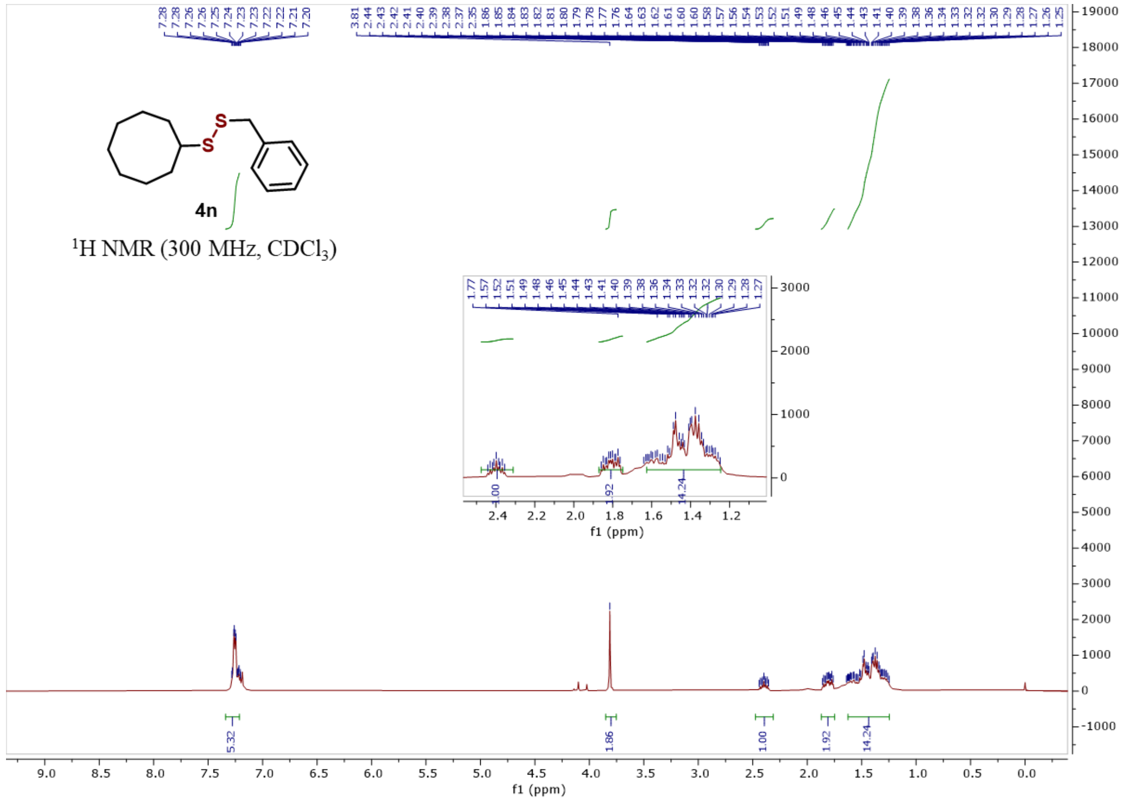

Supplementary Figure 45: ^{1}H NMR (300 MHz, CDCl$_3$) spectrum of compound **4n**.

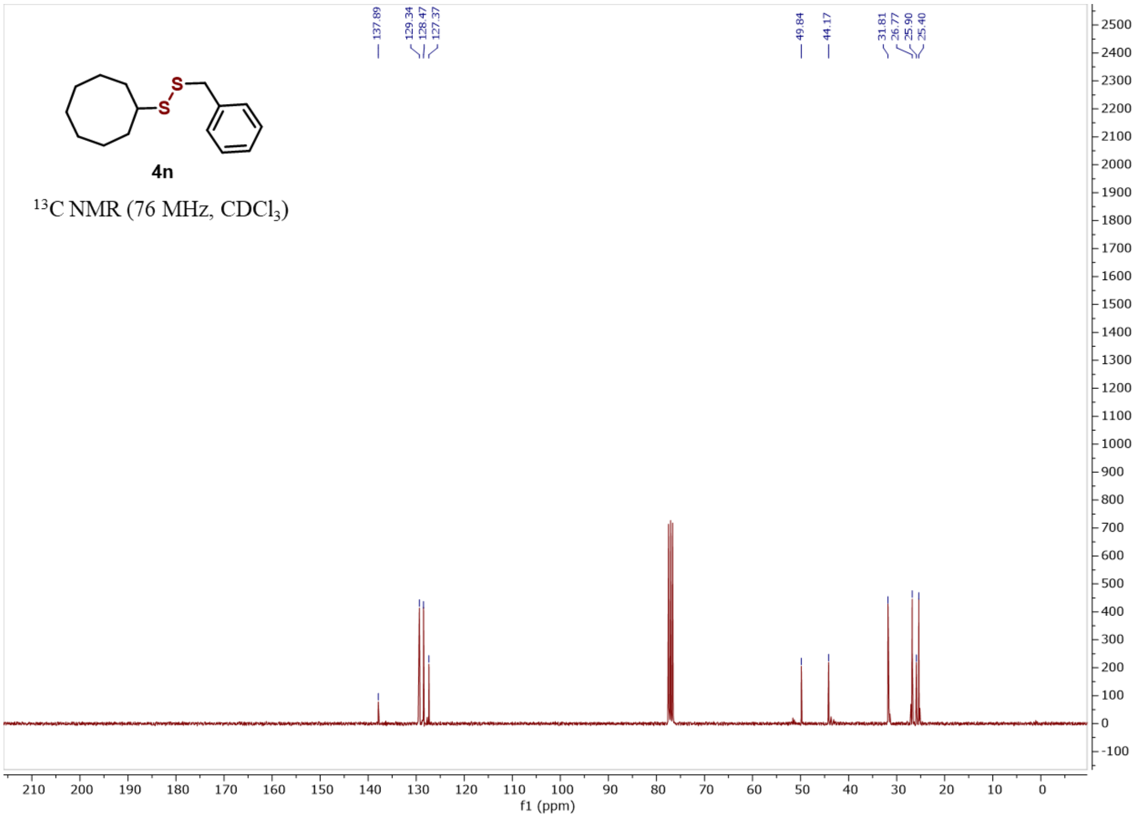

Supplementary Figure 46: ^{13}C NMR (76MHz, CDCl$_3$) spectrum of compound **4n**.

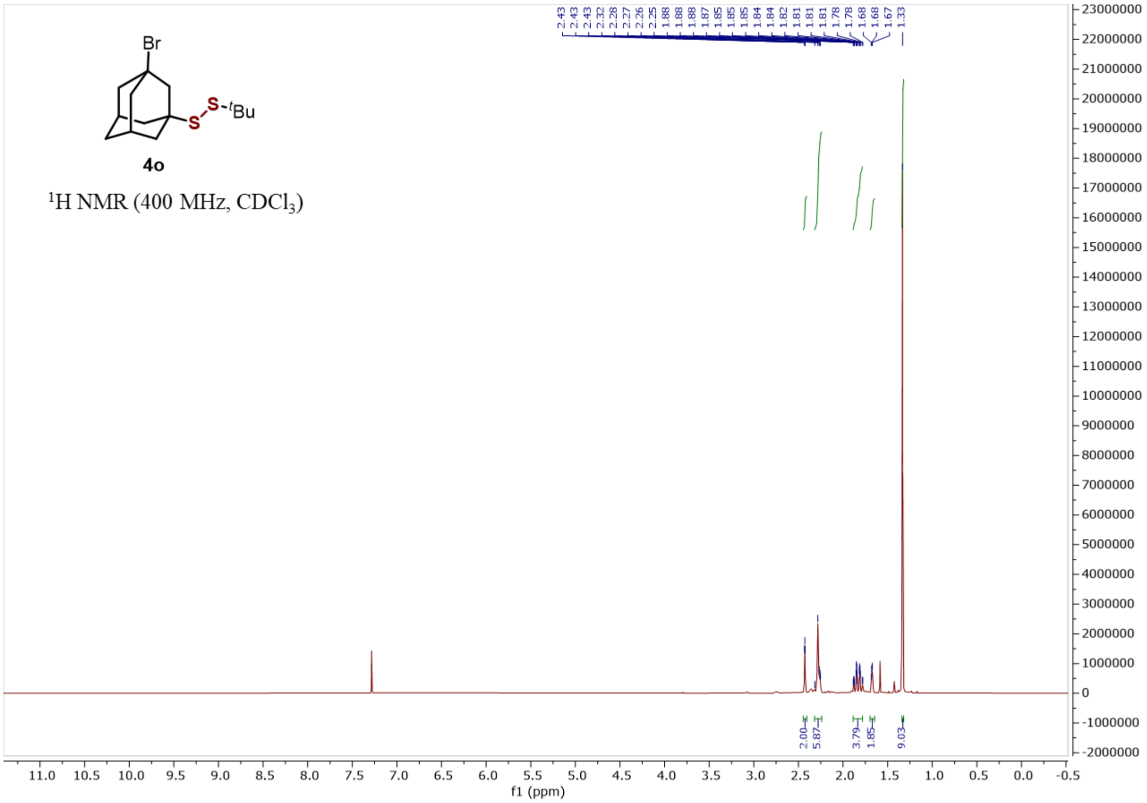

Supplementary Figure 47: ^{1}H NMR (400 MHz, CDCl$_3$) spectrum of compound **4o**.

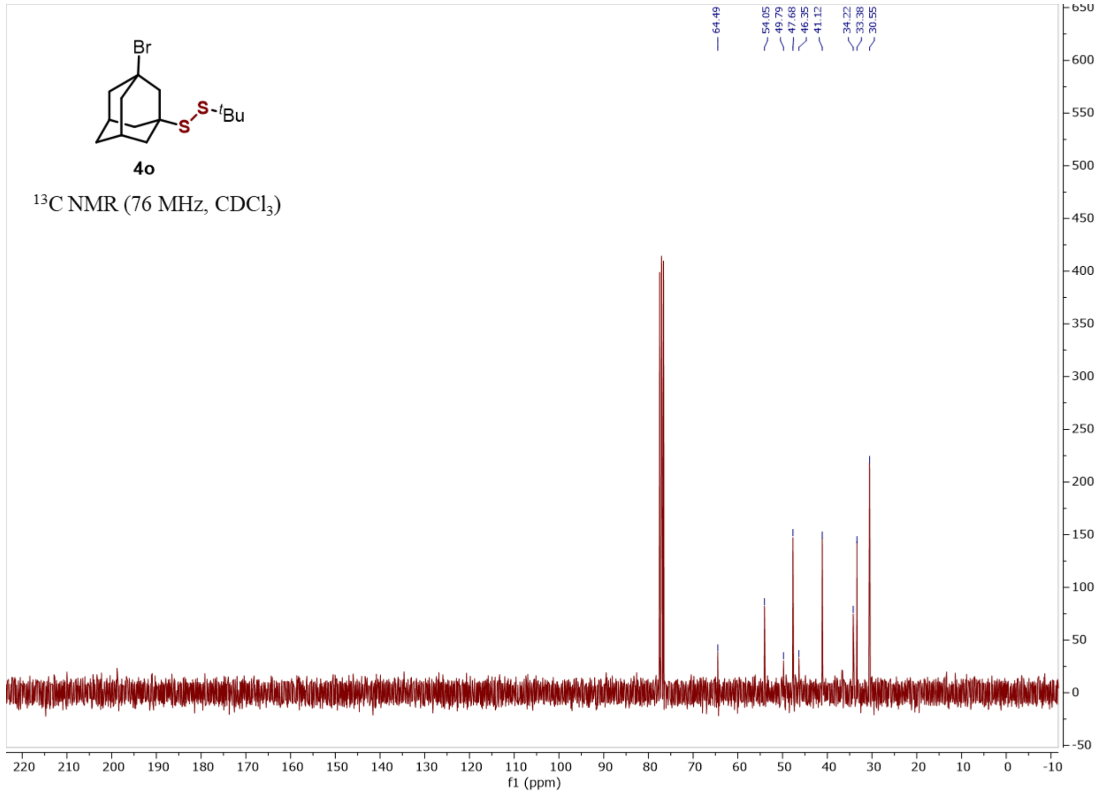

Supplementary Figure 48: ^{13}C NMR (76 MHz, CDCl$_3$) spectrum of compound **4o**.

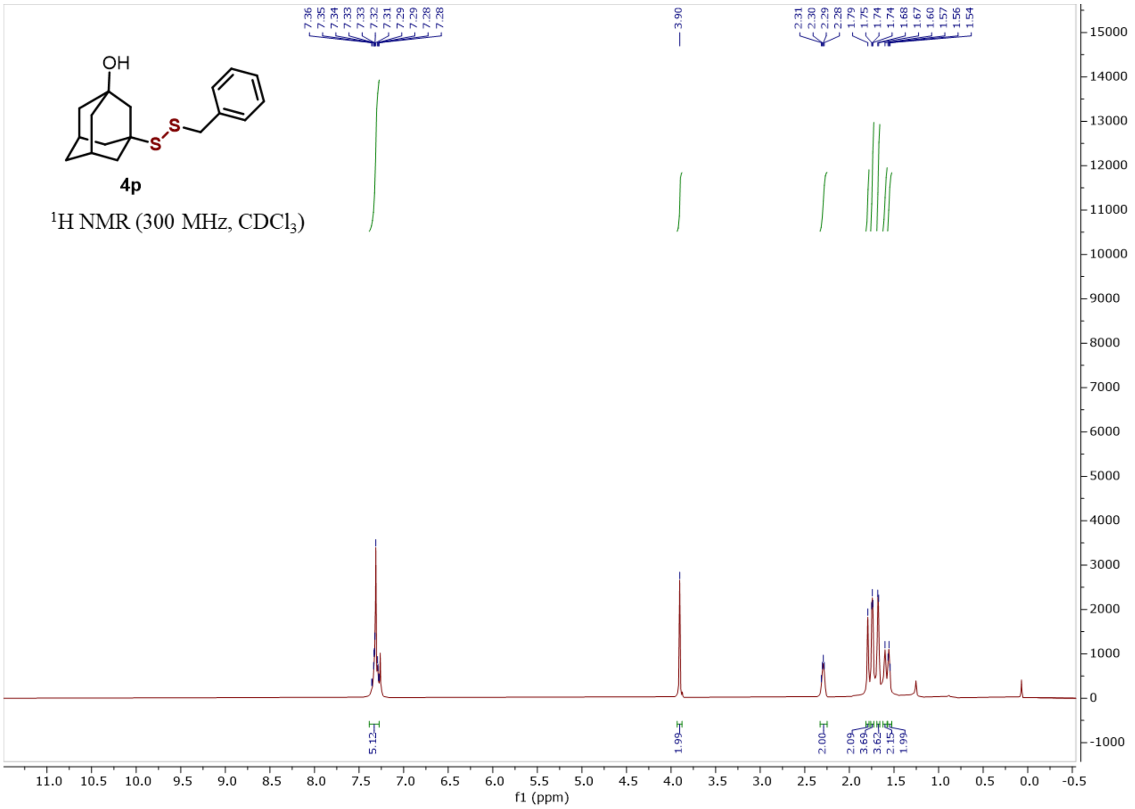

Supplementary Figure 49: ^{1}H NMR (300 MHz, $CDCl_3$) spectrum of compound **4p**.

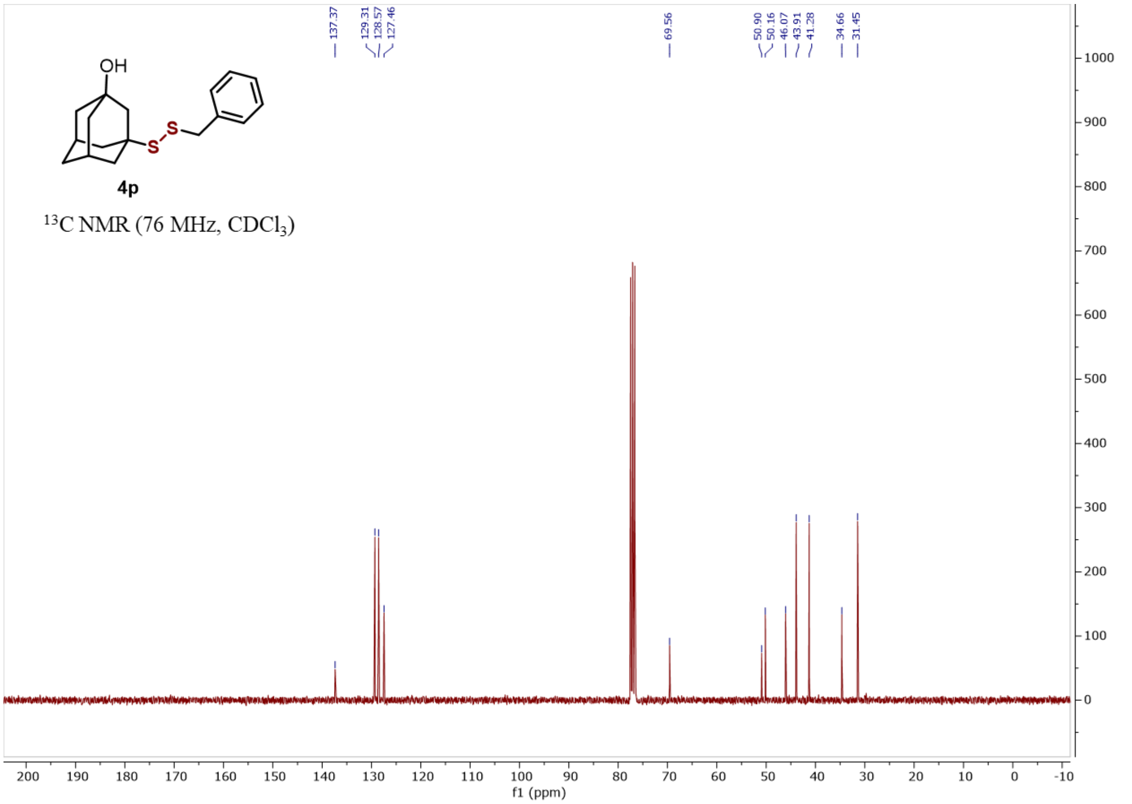

Supplementary Figure 50: ^{13}C NMR (76 MHz, $CDCl_3$) spectrum of compound **4p**.

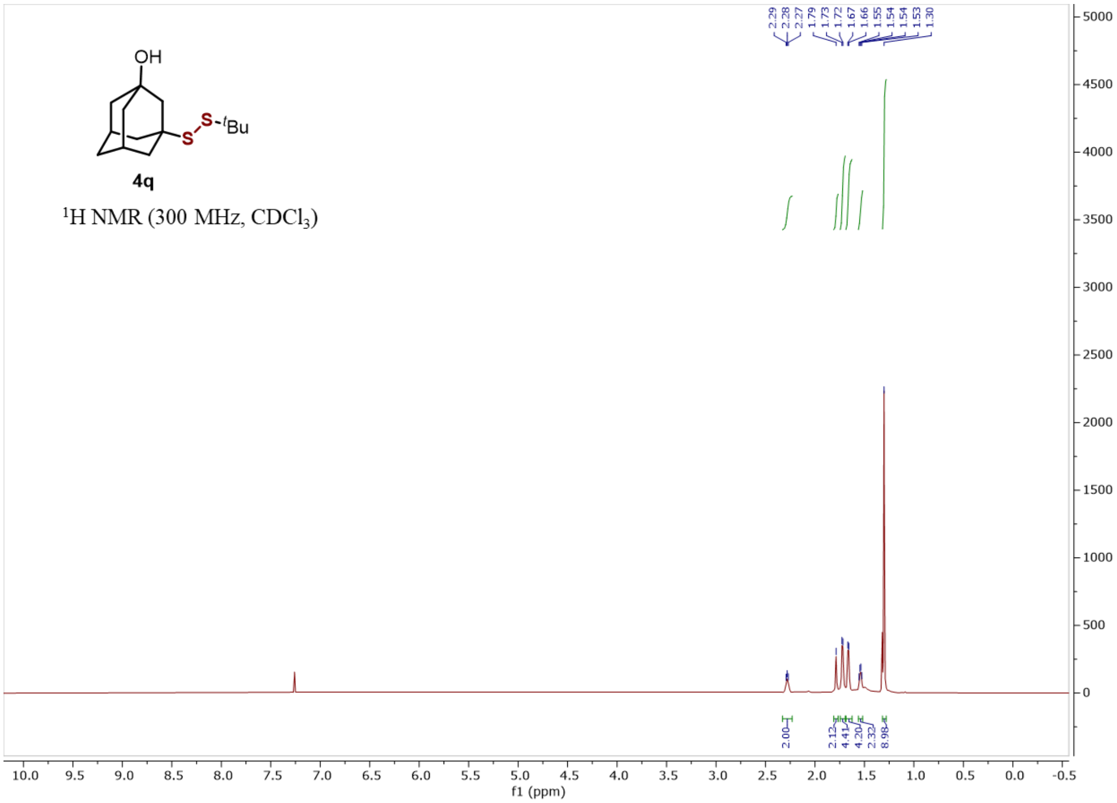

Supplementary Figure 51: ^{1}H NMR (300 MHz, $CDCl_3$) spectrum of compound **4q**.

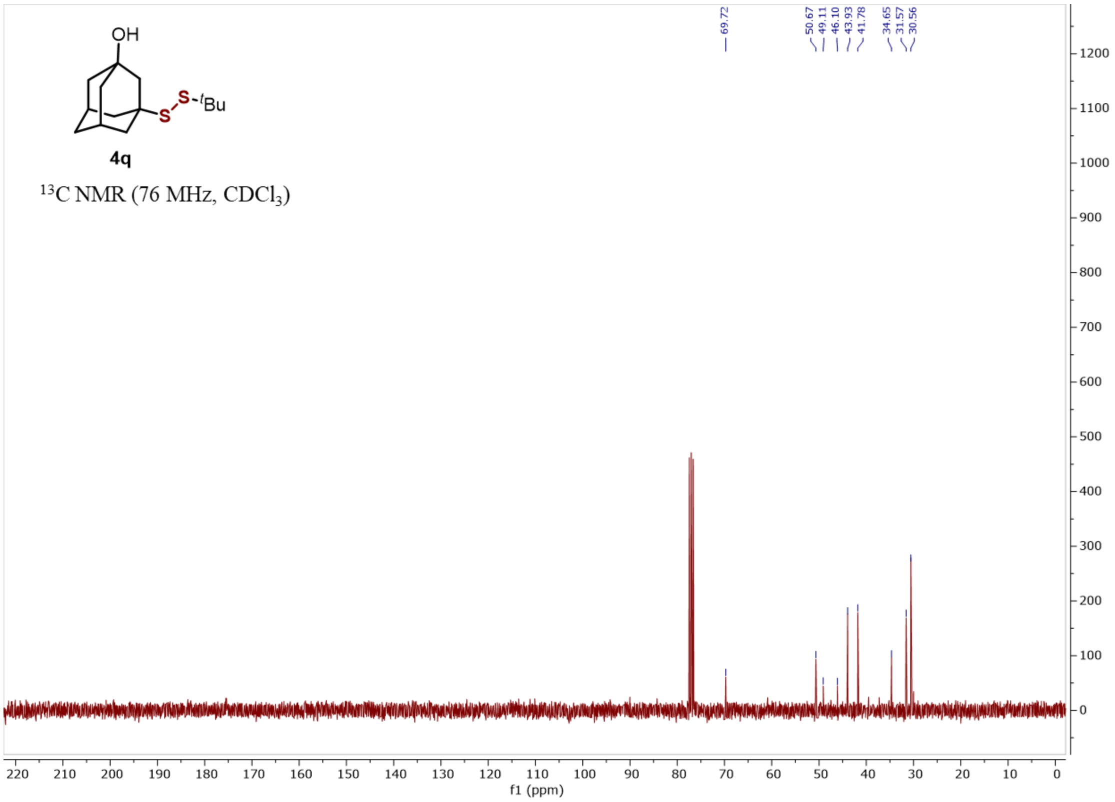

Supplementary Figure 52: ^{13}C NMR (76 MHz, $CDCl_3$) spectrum of compound **4q**.

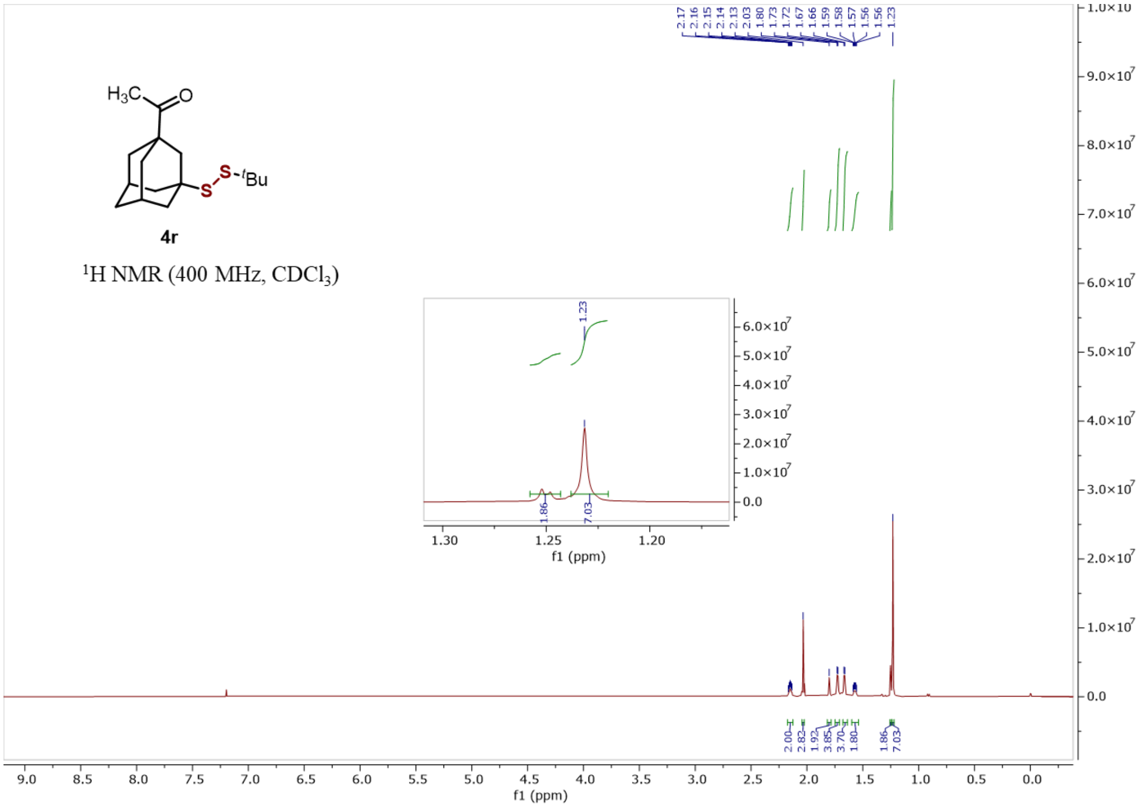

Supplementary Figure 53: ^{1}H NMR (400 MHz, $CDCl_3$) spectrum of compound **4r**.

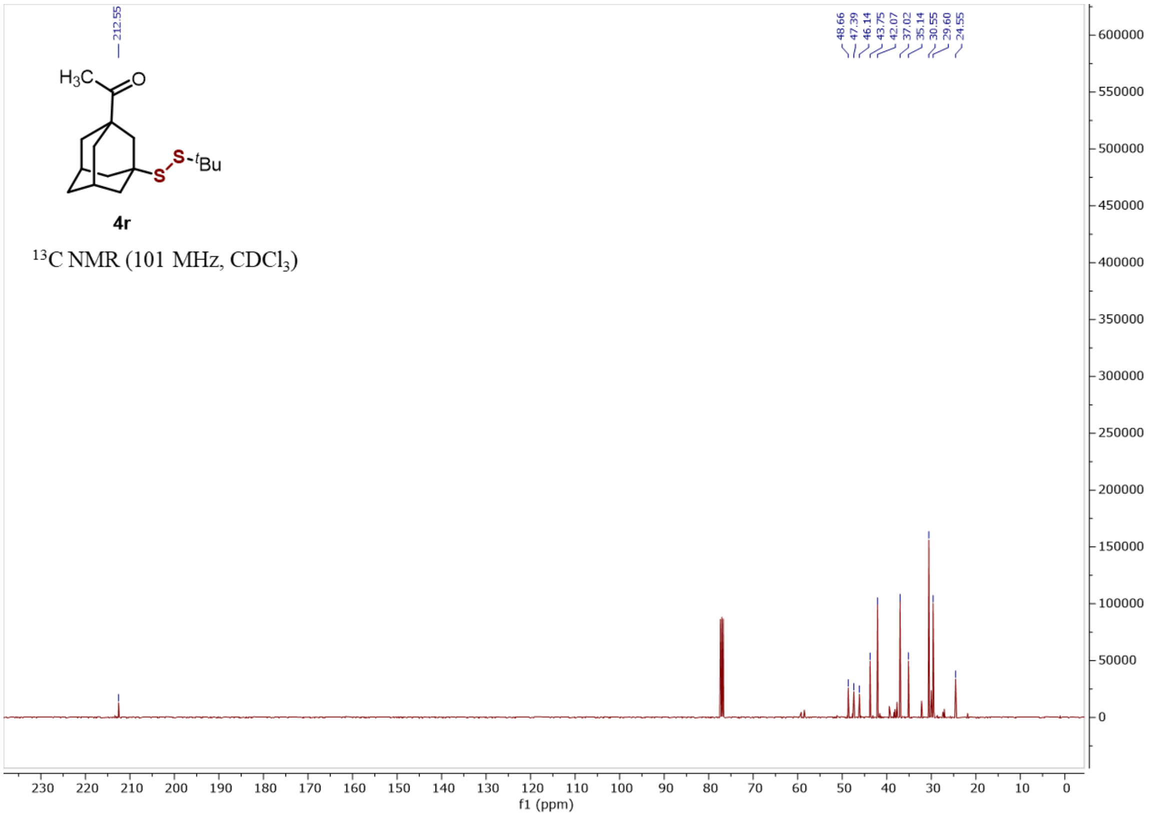

Supplementary Figure 54: ^{13}C NMR (101 MHz, $CDCl_3$) spectrum of compound **4r**.

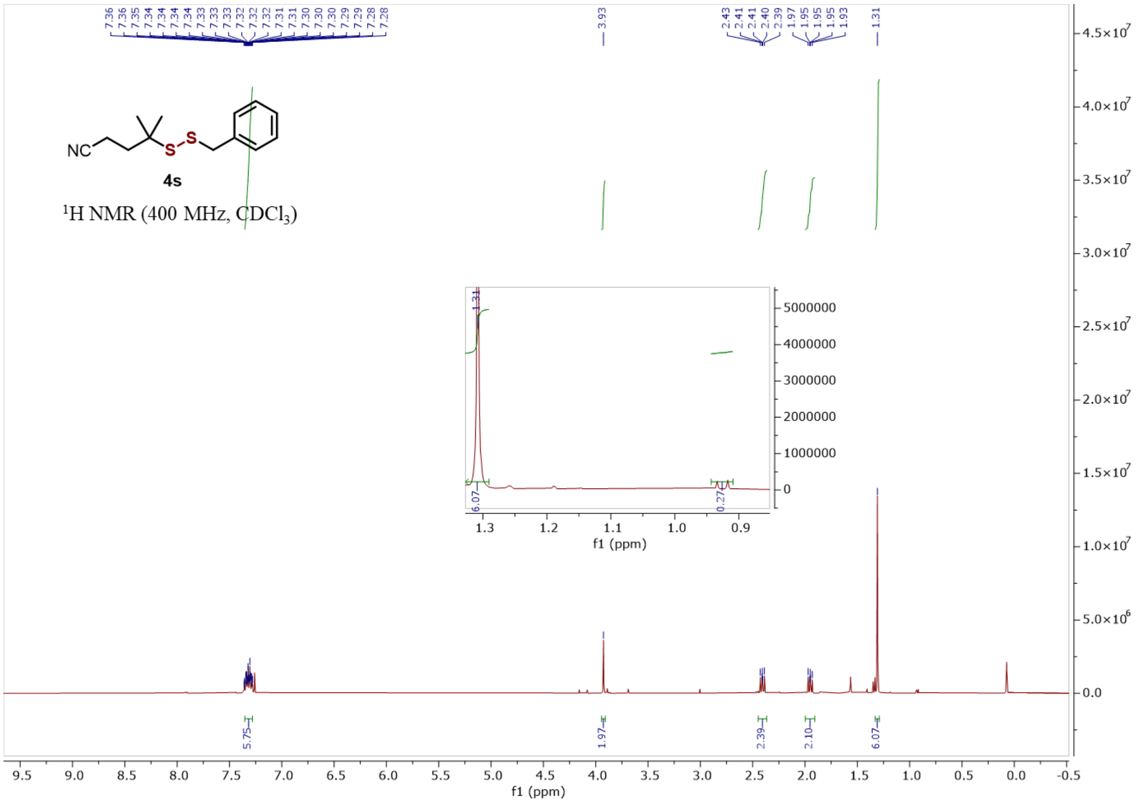

Supplementary Figure 55: ^{1}H NMR (400 MHz, $CDCl_3$) spectrum of compound **4s**.

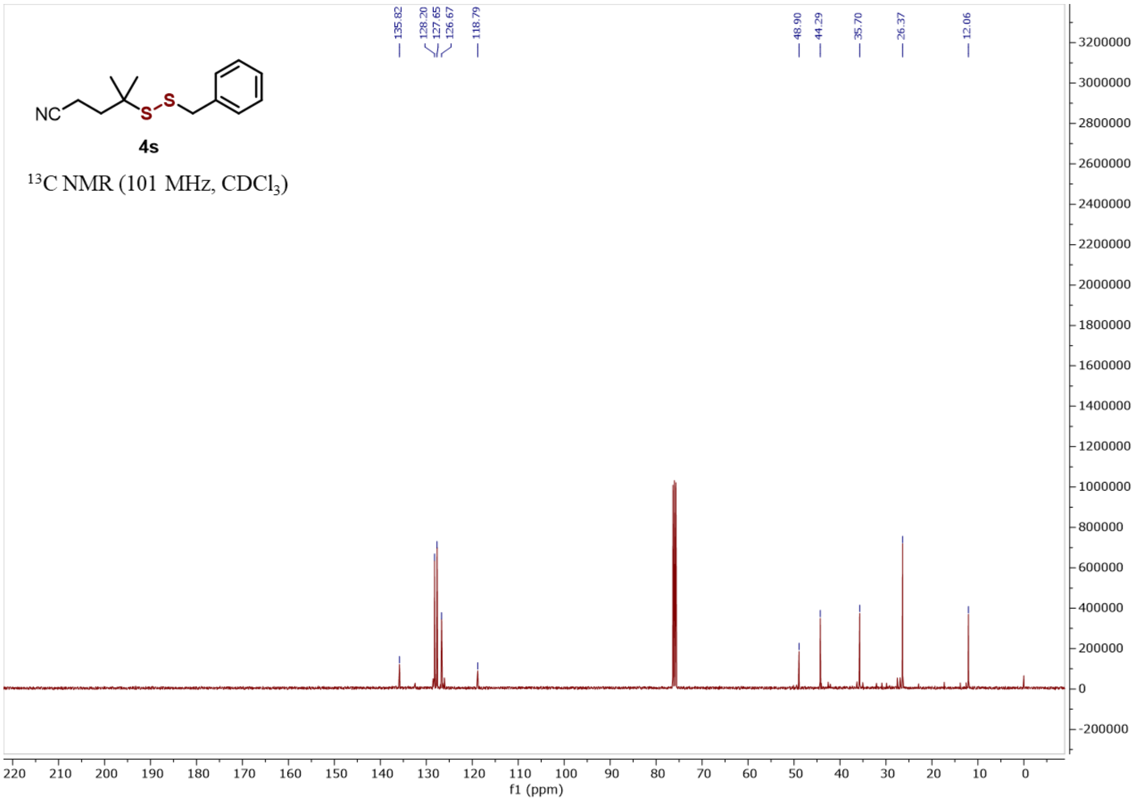

Supplementary Figure 56: ^{13}C NMR (101 MHz, $CDCl_3$) spectrum of compound **4s**.

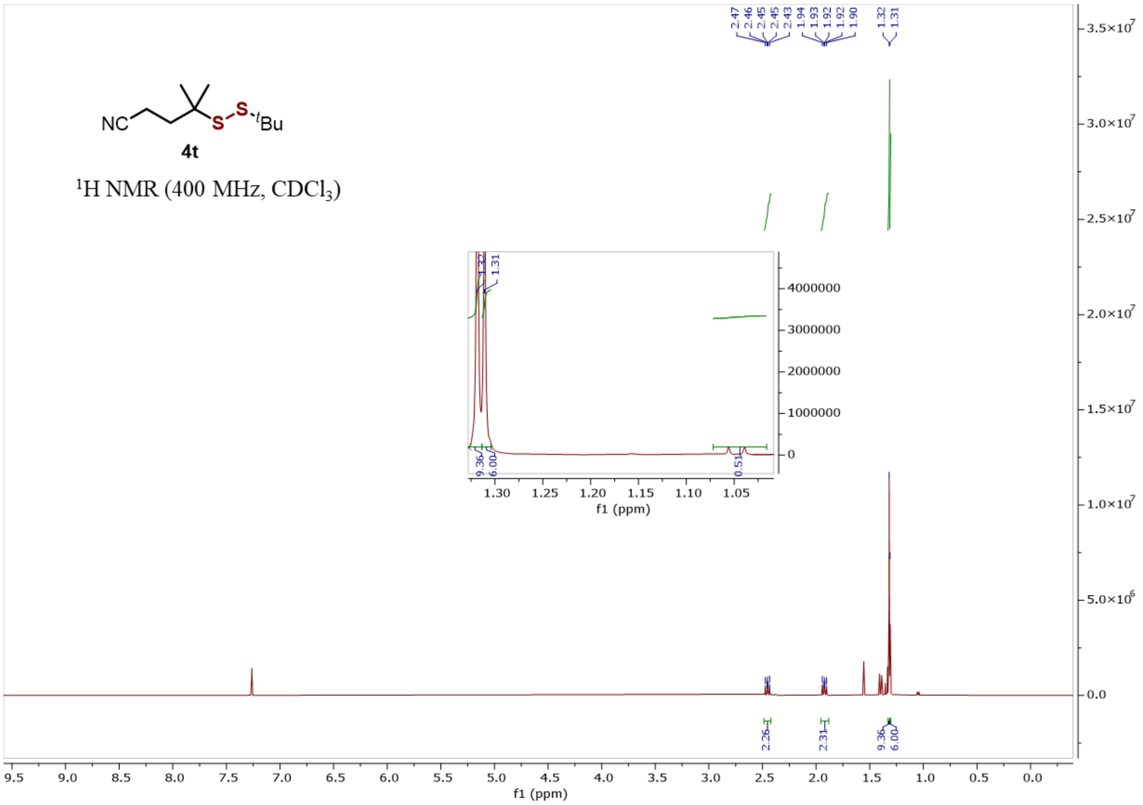

Supplementary Figure 57: ^{1}H NMR (400 MHz, $CDCl_3$) spectrum of compound **4t**.

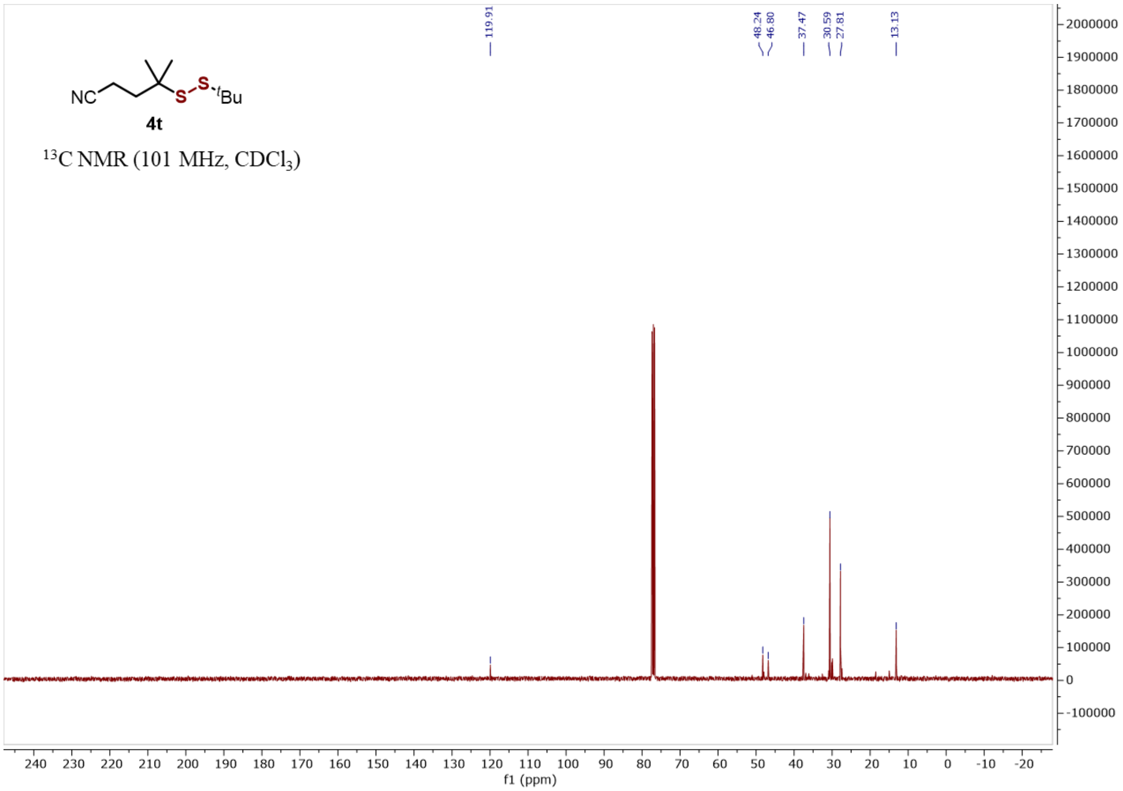

Supplementary Figure 58: ^{13}C NMR (101 MHz, $CDCl_3$) spectrum of compound **4t**.

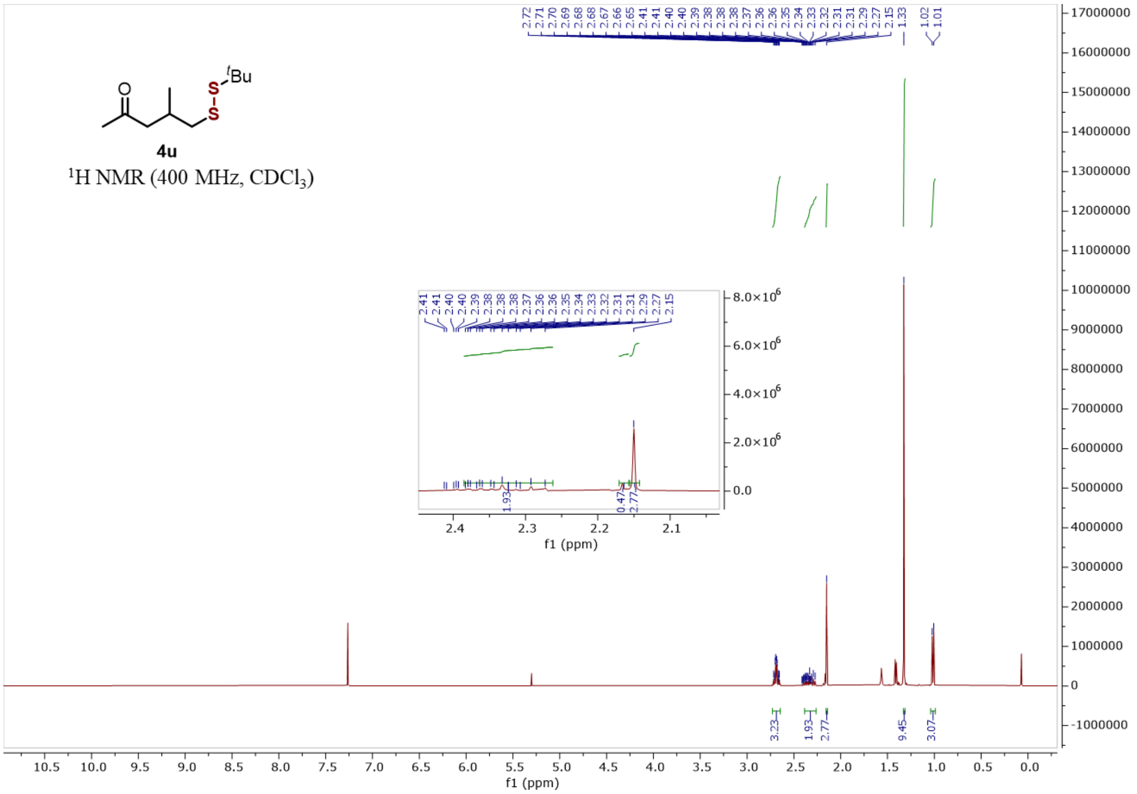

Supplementary Figure 59: ^{1}H NMR (400 MHz, $CDCl_3$) spectrum of compound **4u**.

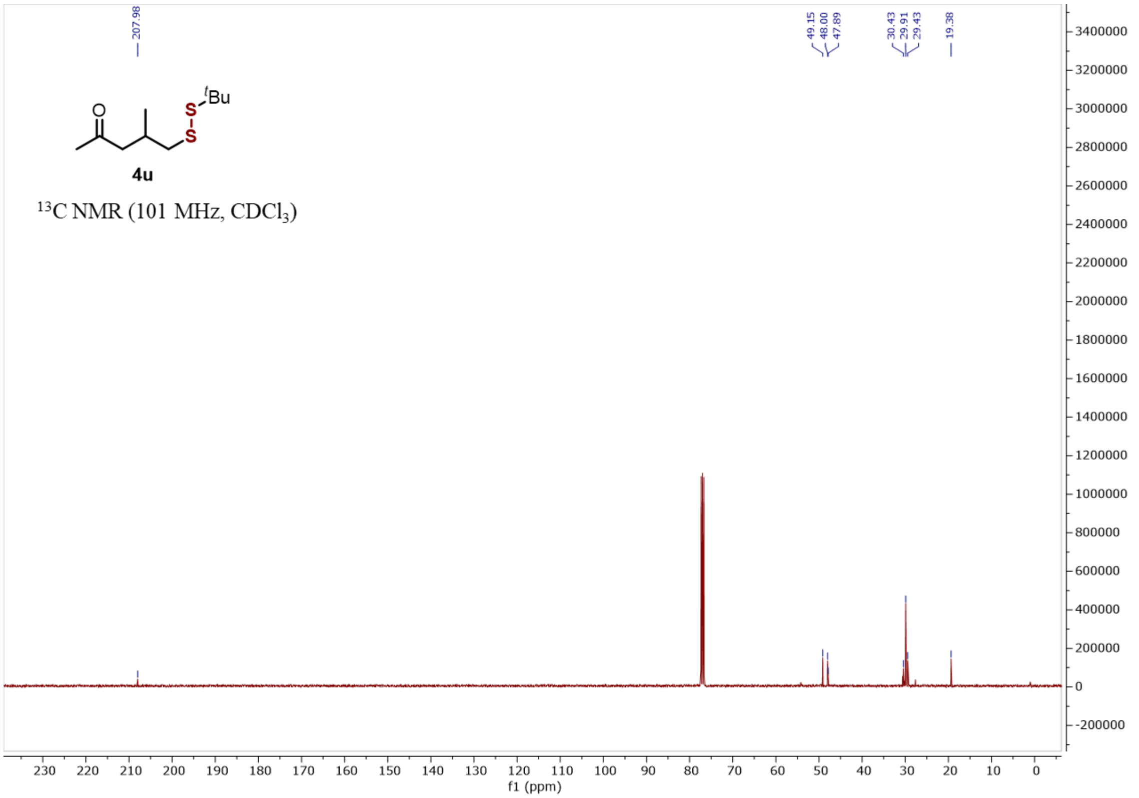

Supplementary Figure 60: ^{13}C NMR (101 MHz, $CDCl_3$) spectrum of compound **4u**.

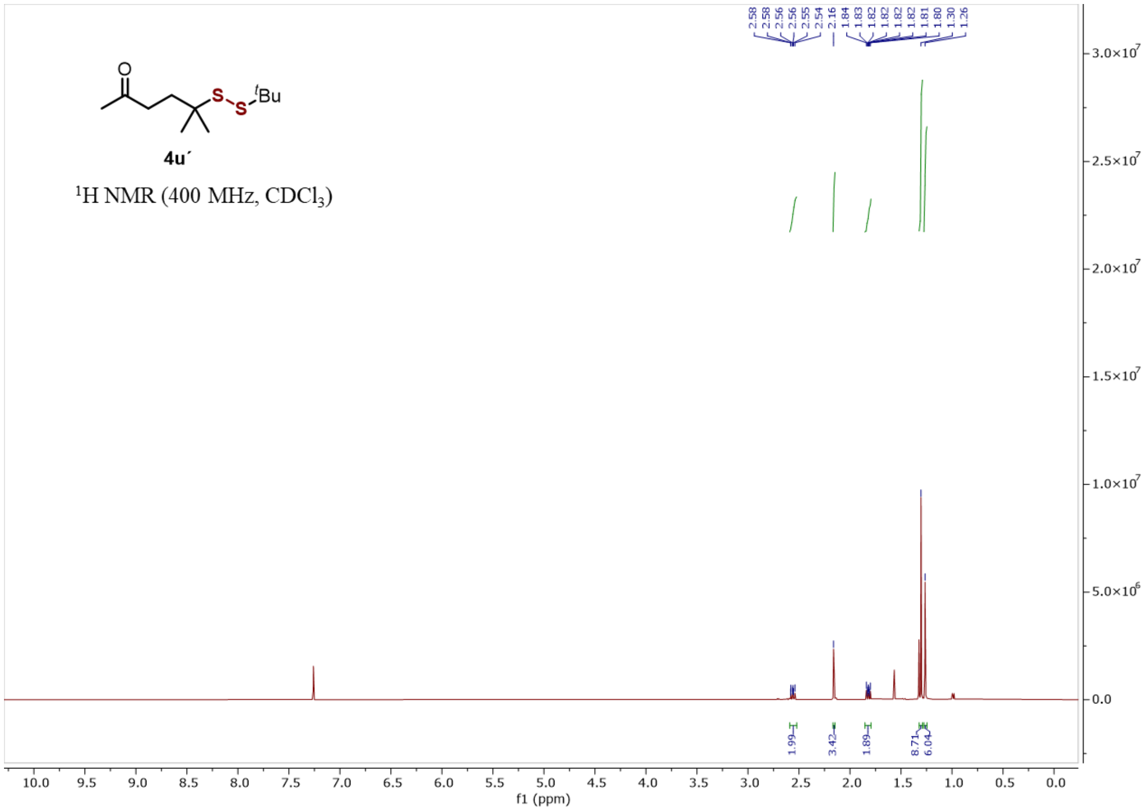

Supplementary Figure 61: ^{1}H NMR (400 MHz, CDCl$_3$) spectrum of compound **4u'**.

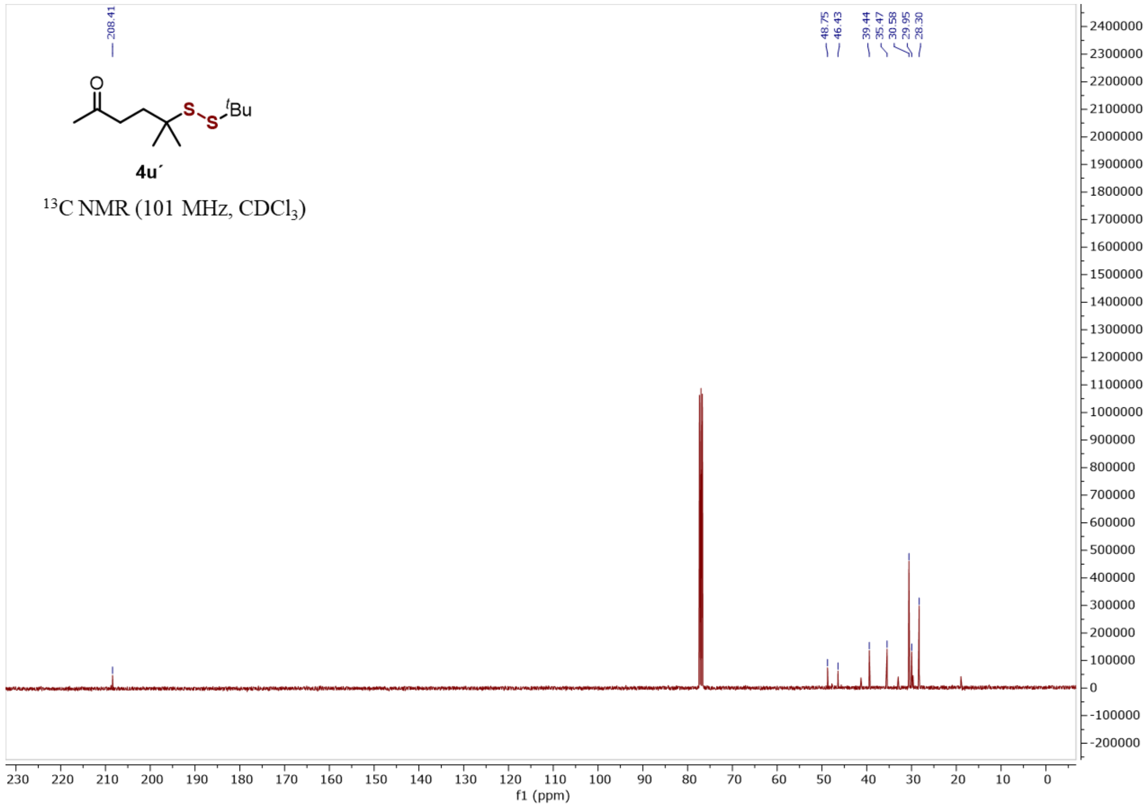

Supplementary Figure 62: ^{13}C NMR (101 MHz, CDCl$_3$) spectrum of compound **4u'**.

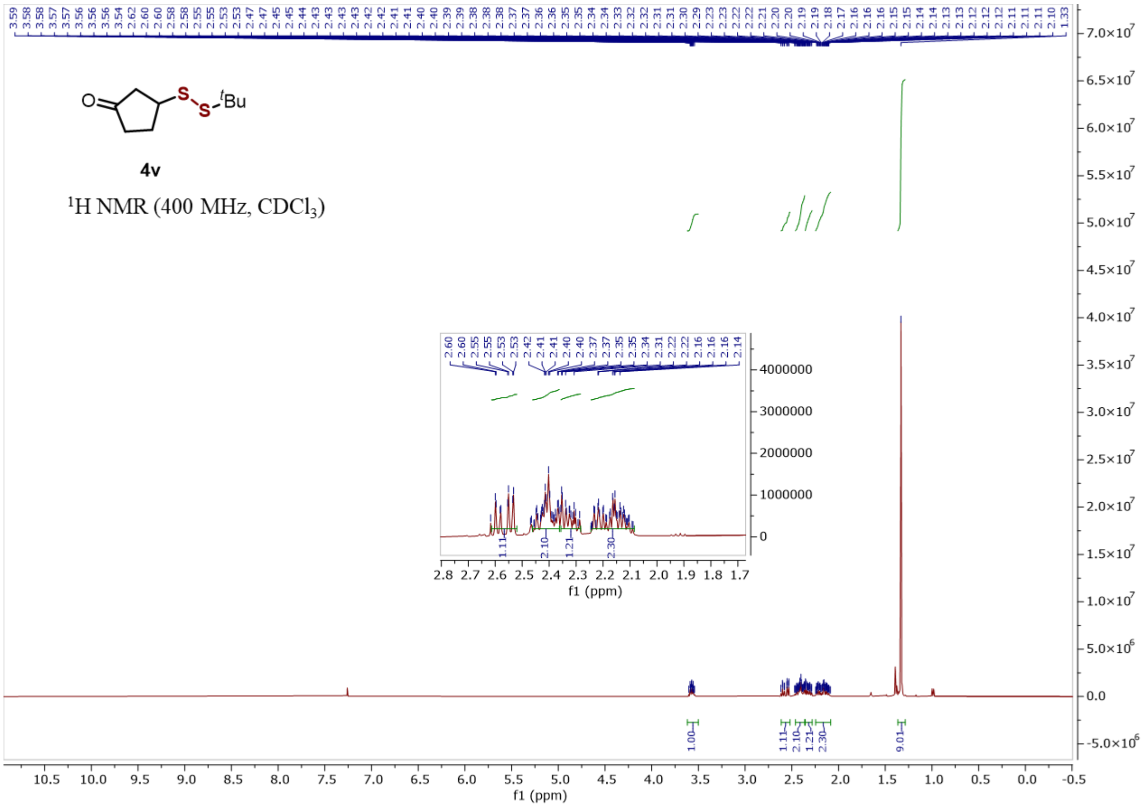

Supplementary Figure 63: ^{1}H NMR (400 MHz, CDCl$_3$) spectrum of compound **4v**.

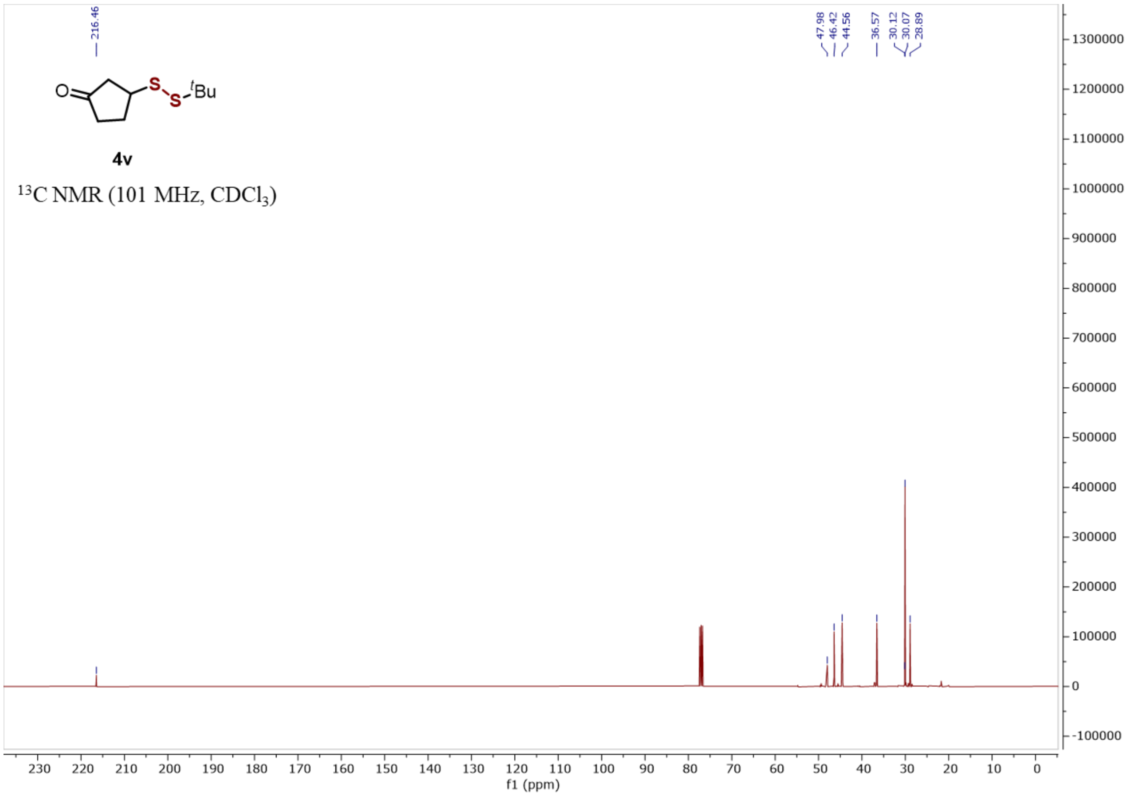

Supplementary Figure 64: ^{13}C NMR (101 MHz, CDCl$_3$) spectrum of compound **4v**.

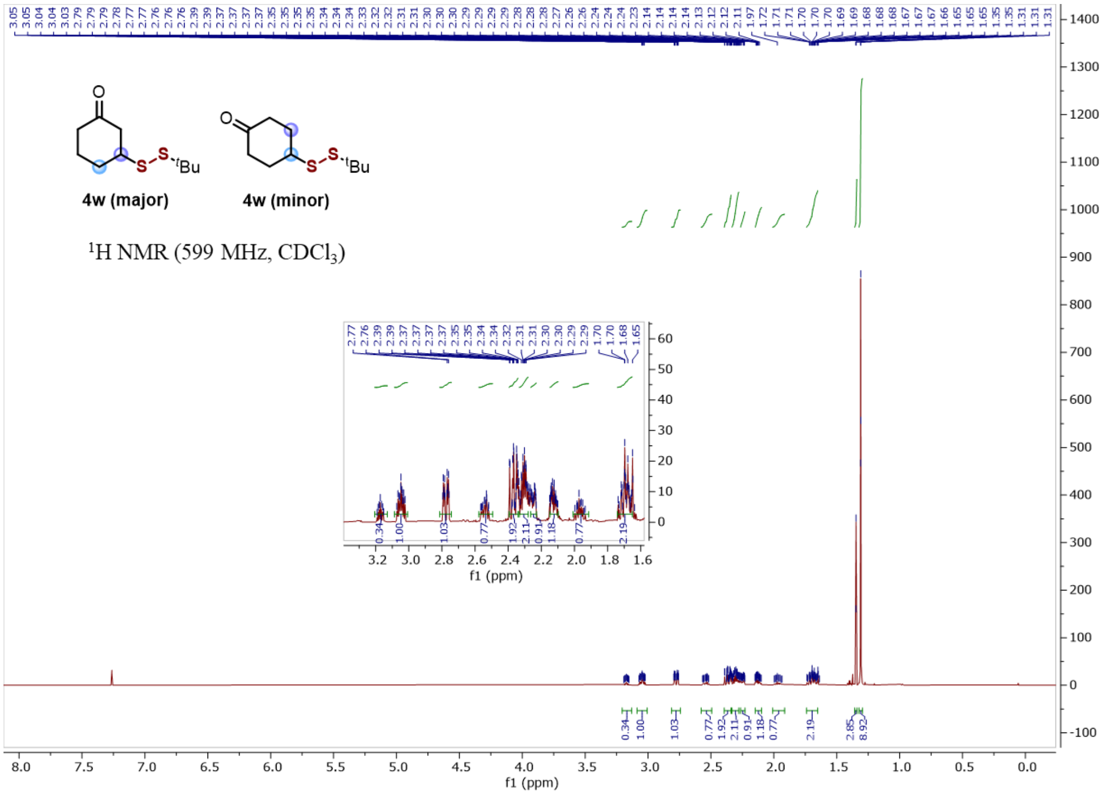

Supplementary Figure 65: ^{1}H NMR (599 MHz, $CDCl_3$) spectrum of compound **4w**.

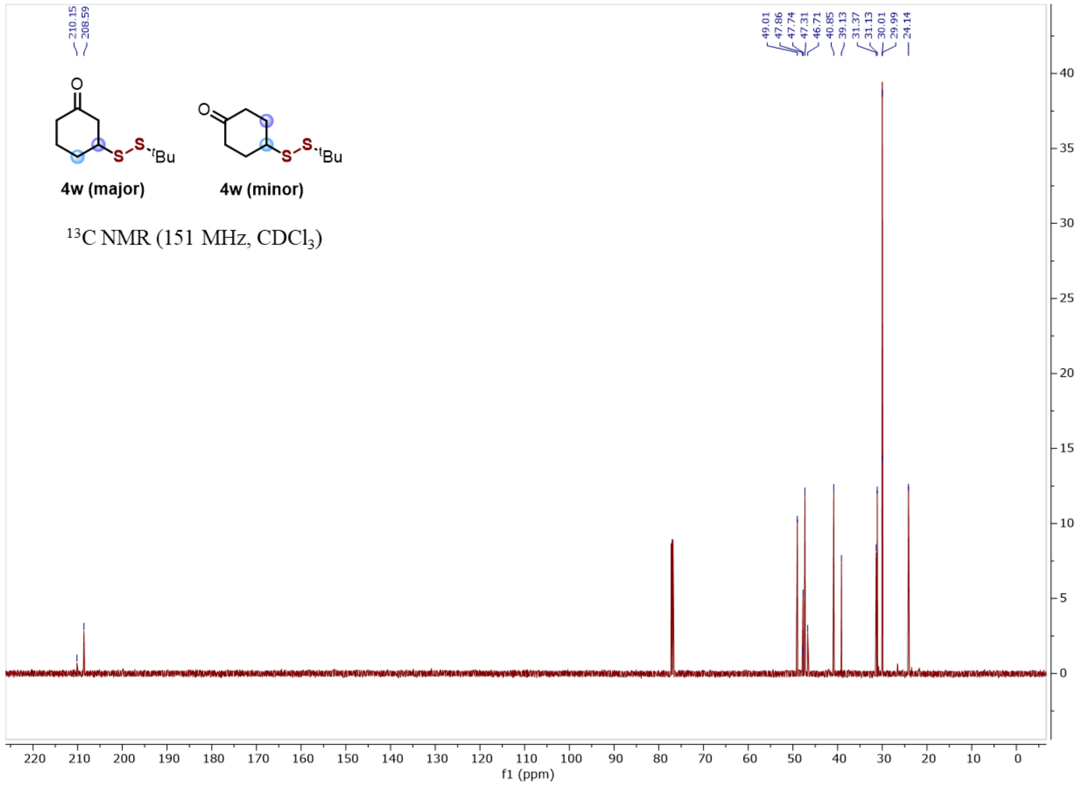

Supplementary Figure 66: ^{13}C NMR (151 MHz, $CDCl_3$) spectrum of compound **4w**.

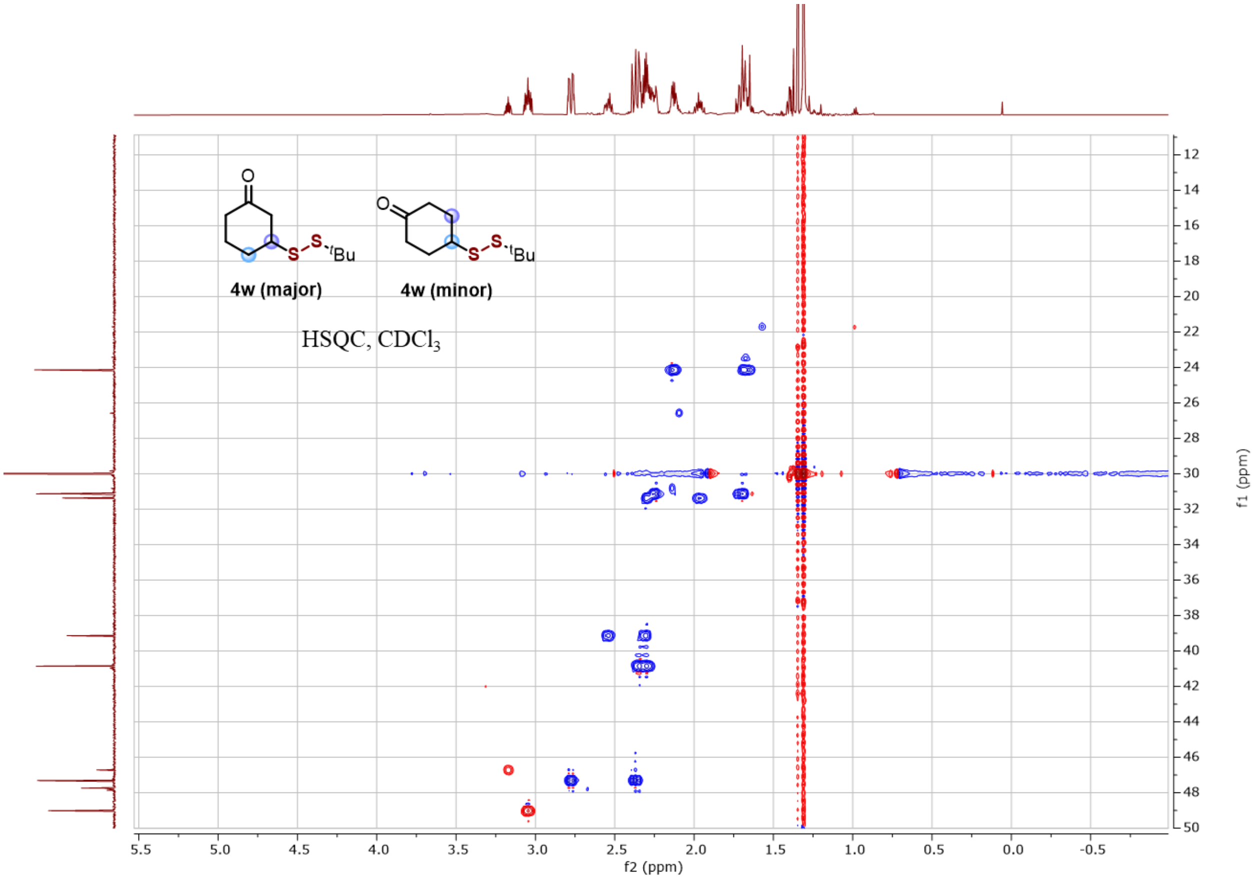

Supplementary Figure 67: HSQC spectrum of compound **4w**.

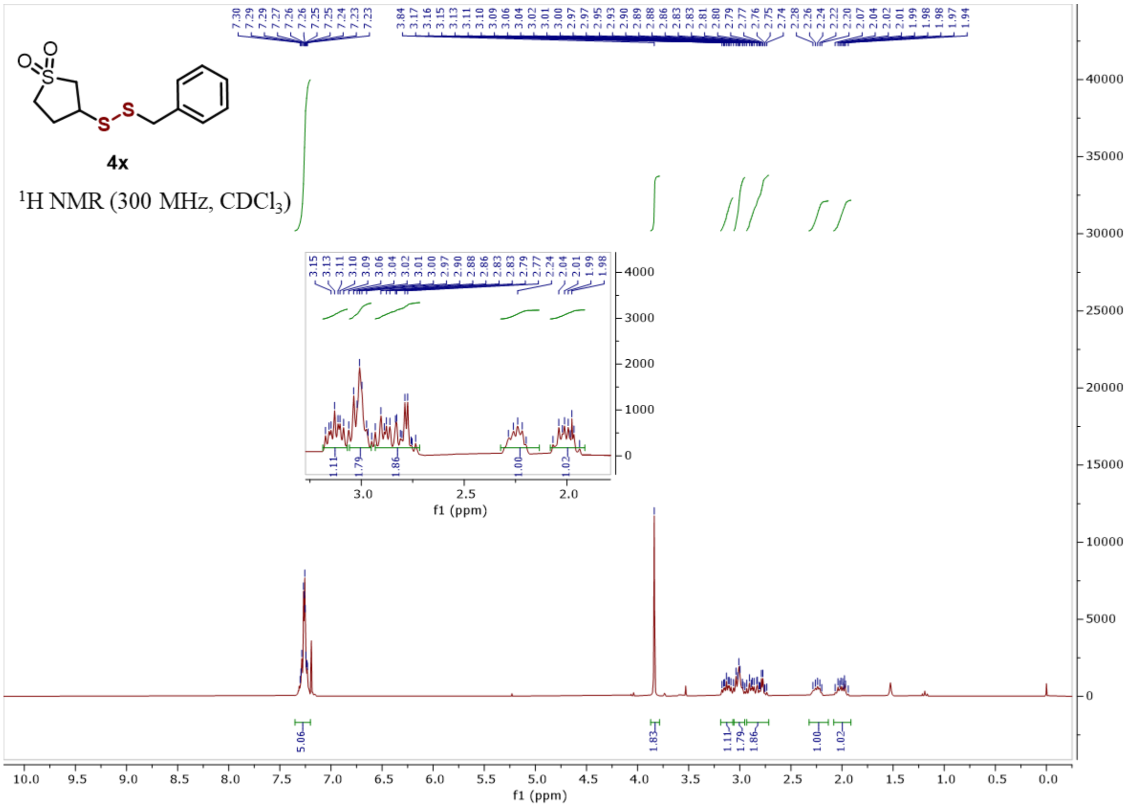

Supplementary Figure 68: ^{1}H NMR (300 MHz, $CDCl_3$) spectrum of compound **4x**.

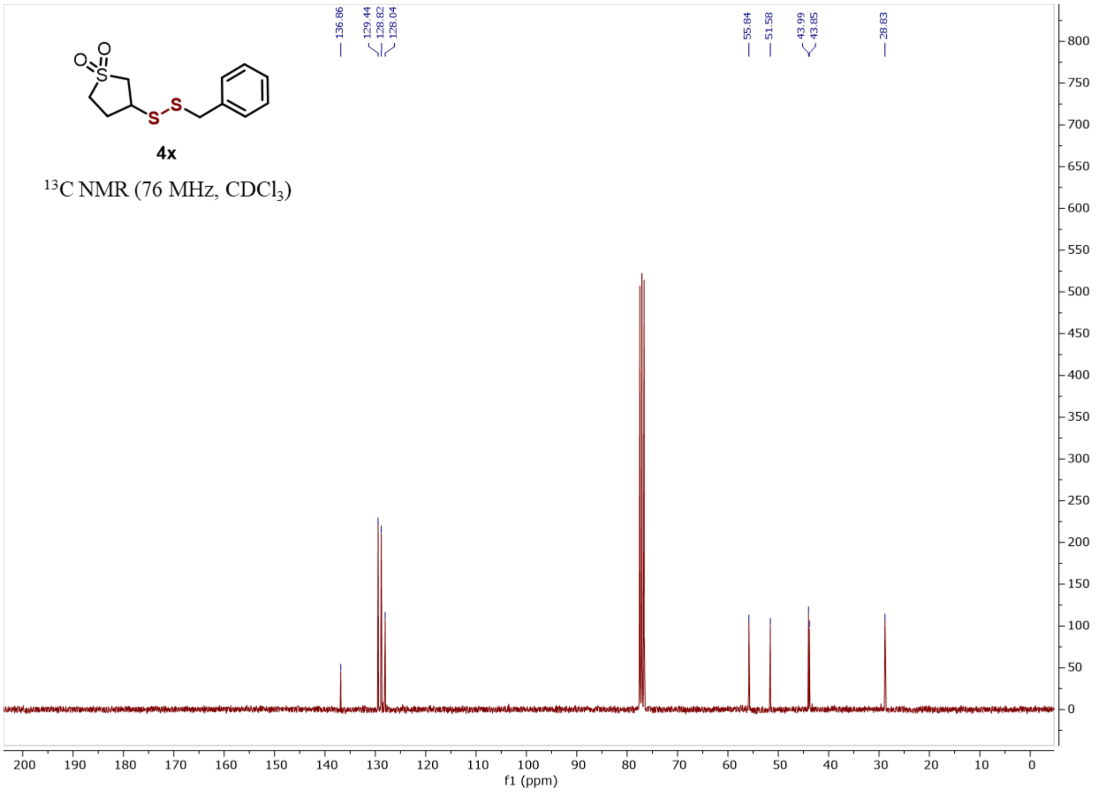

Supplementary Figure 69: ^{13}C NMR (76 MHz, $CDCl_3$) spectrum of compound **4x**.

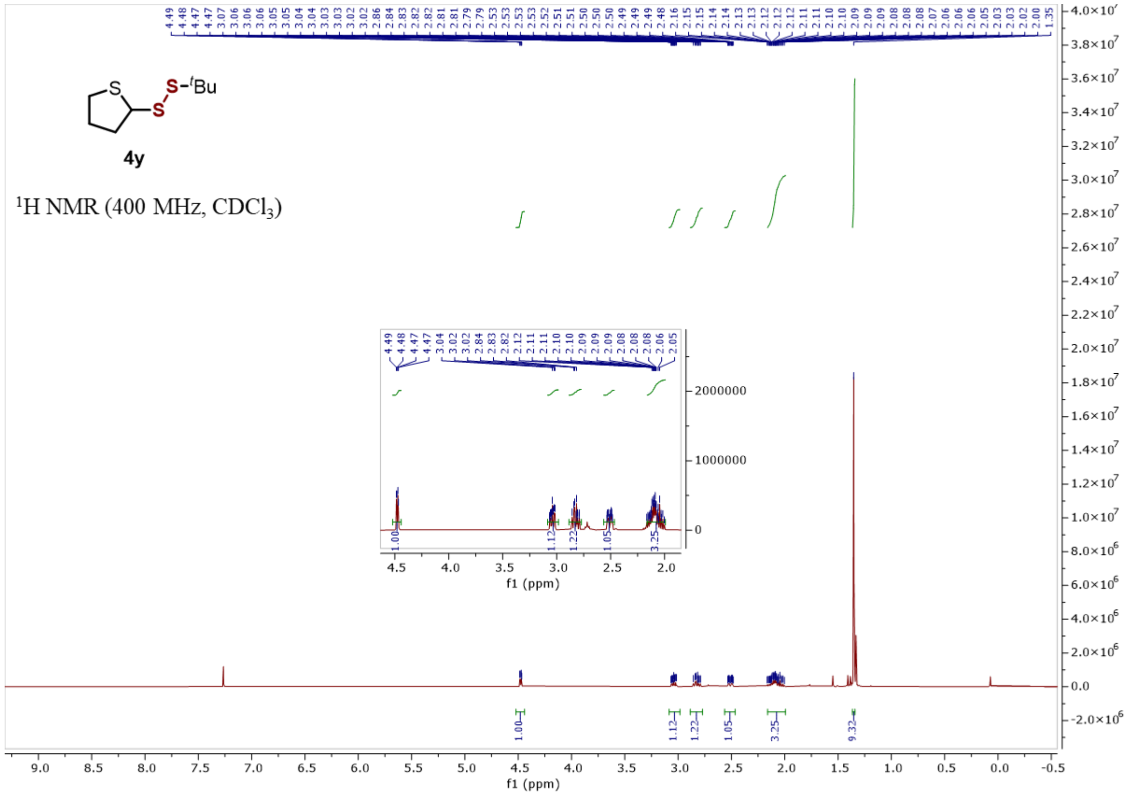

Supplementary Figure 70: ^{1}H NMR (400 MHz, $CDCl_3$) spectrum of compound **4y**.

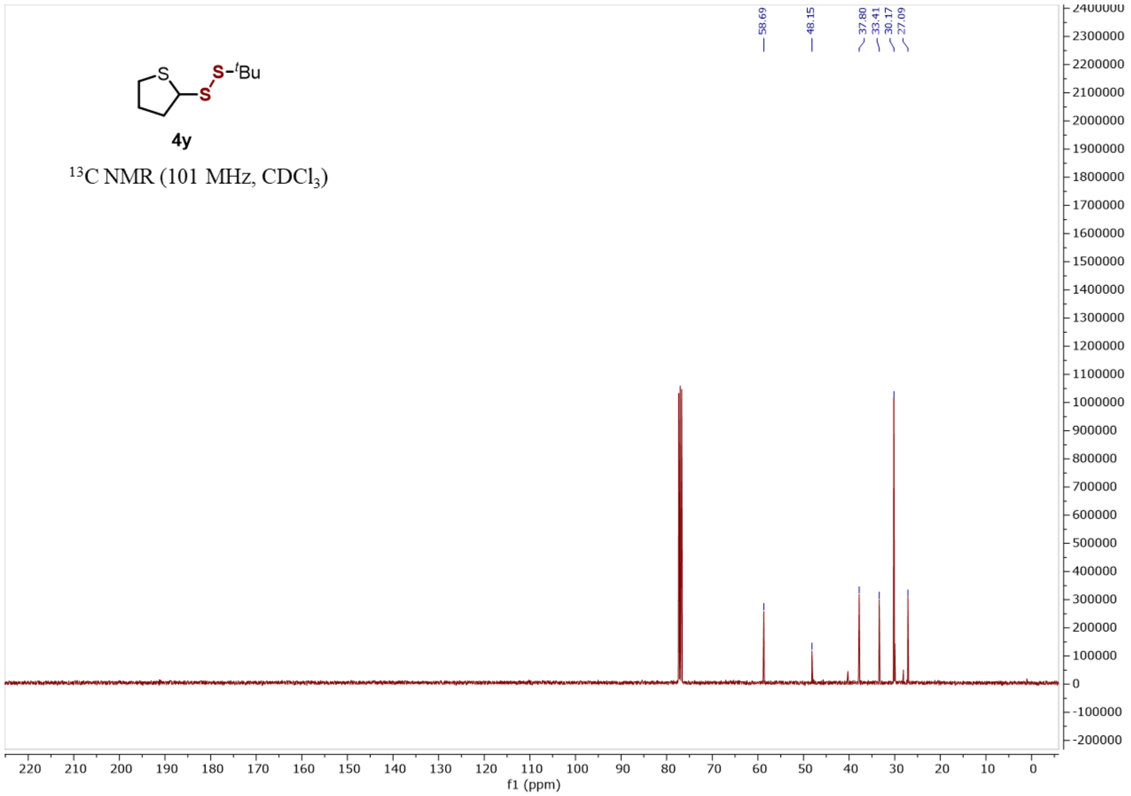

Supplementary Figure 71: ^{13}C NMR (101 MHz, $CDCl_3$) spectrum of compound **4y**.

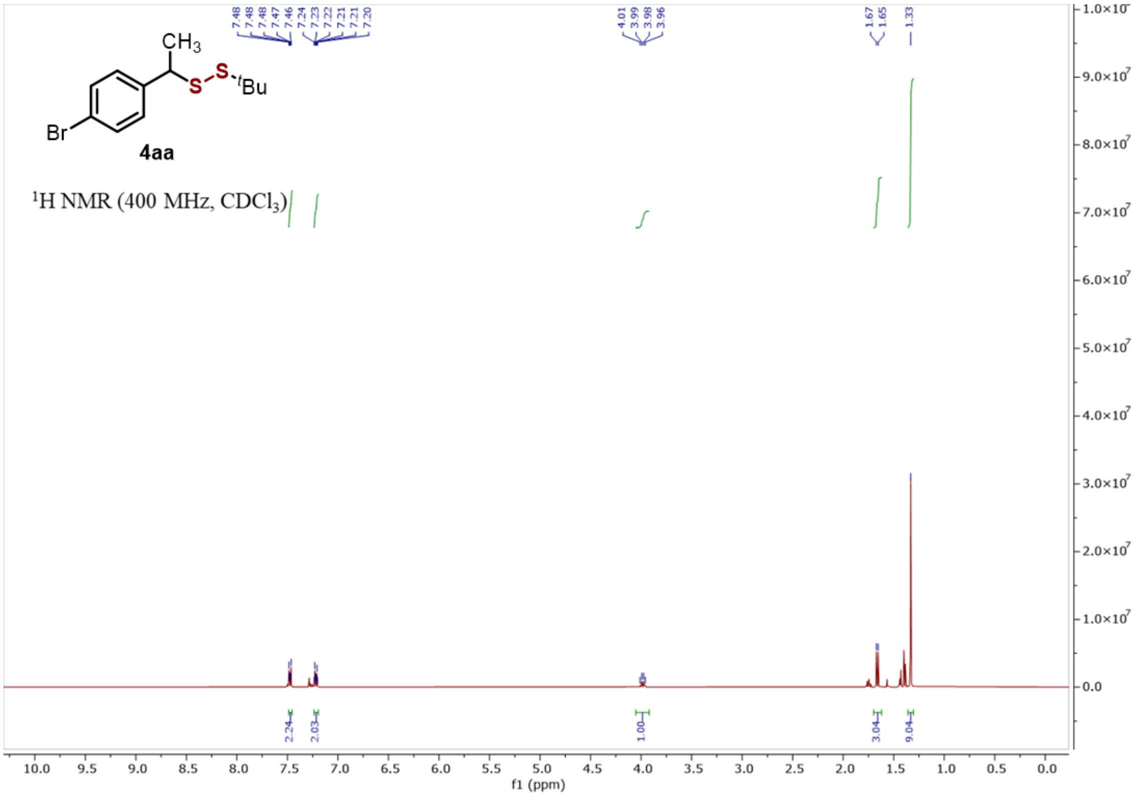

Supplementary Figure 72: ^{1}H NMR (400 MHz, $CDCl_3$) spectrum of compound **4aa**.

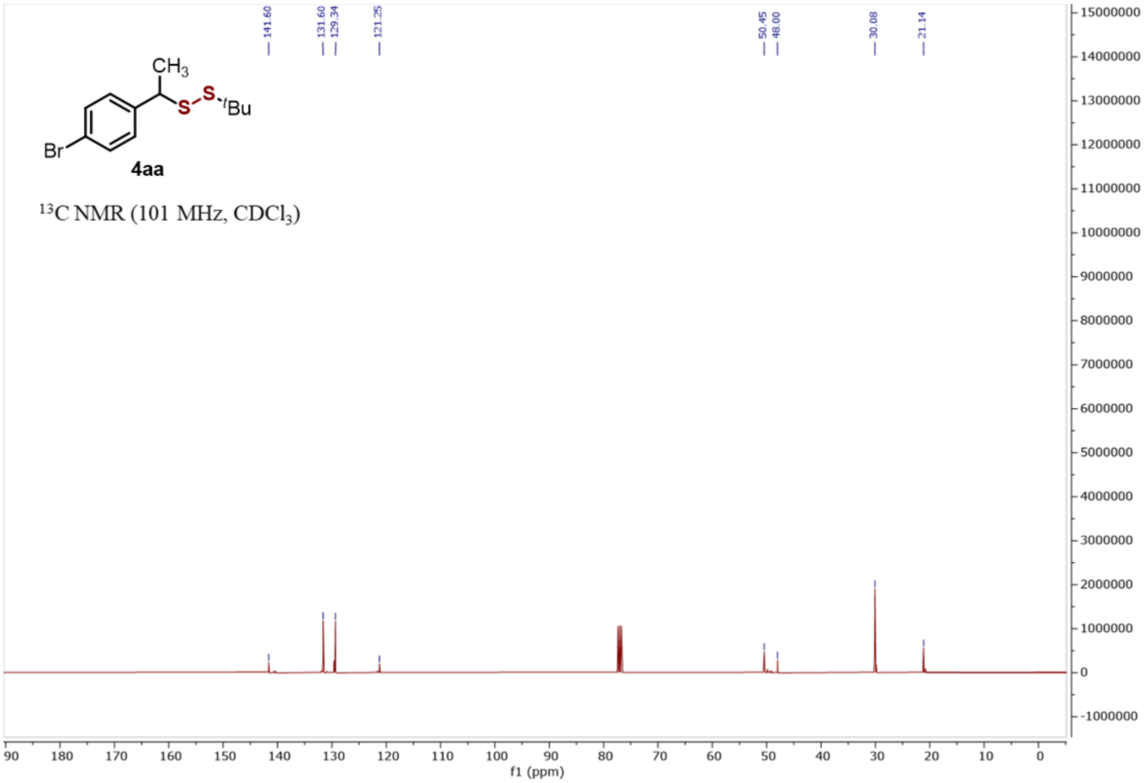

Supplementary Figure 73: ^{13}C NMR (101 MHz, $CDCl_3$) spectrum of compound **4aa**.

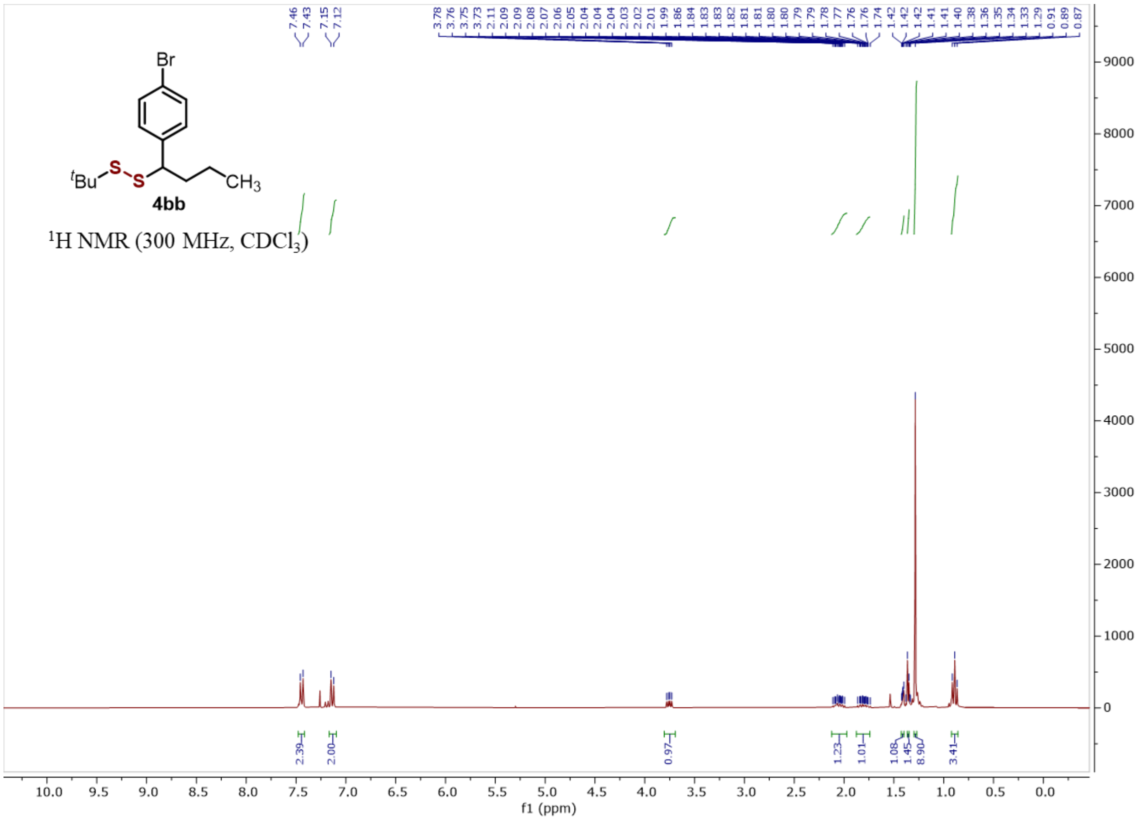

Supplementary Figure 74: ^{1}H NMR (300 MHz, $CDCl_3$) spectrum of compound **4bb**.

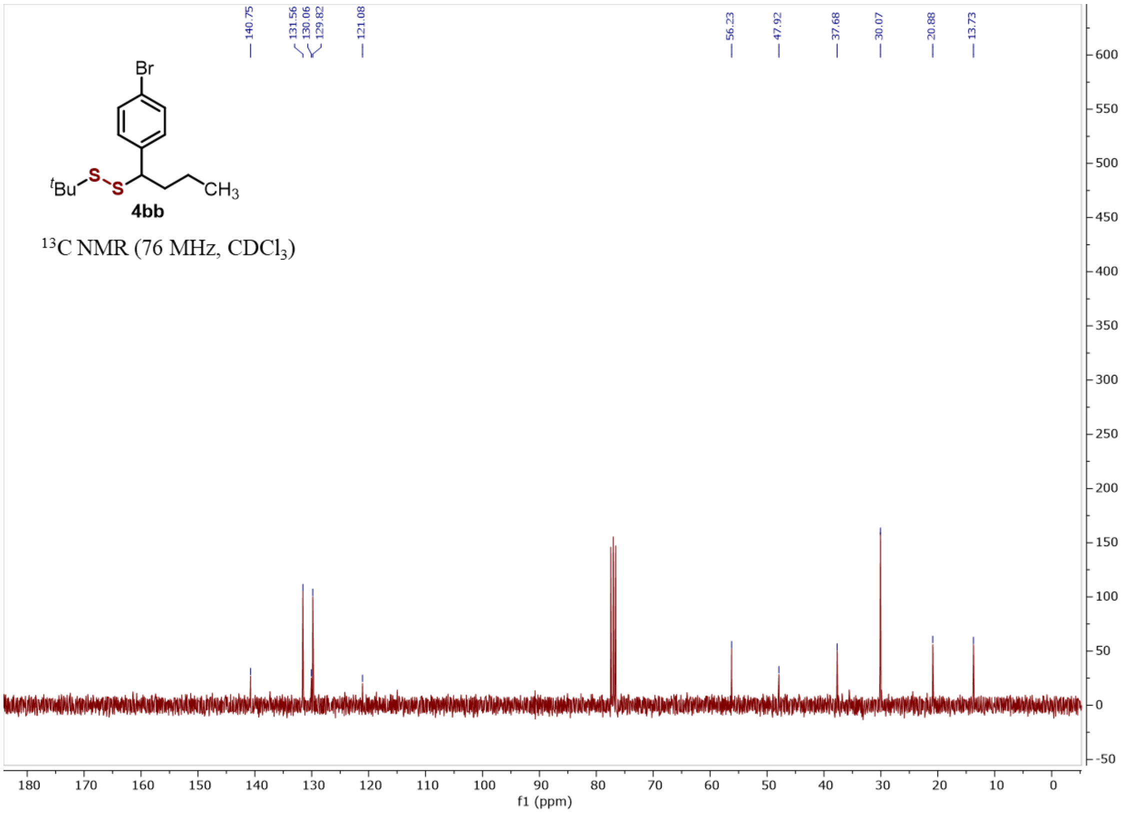

Supplementary Figure 75: ^{13}C NMR (76 MHz, $CDCl_3$) spectrum of compound **4bb**.

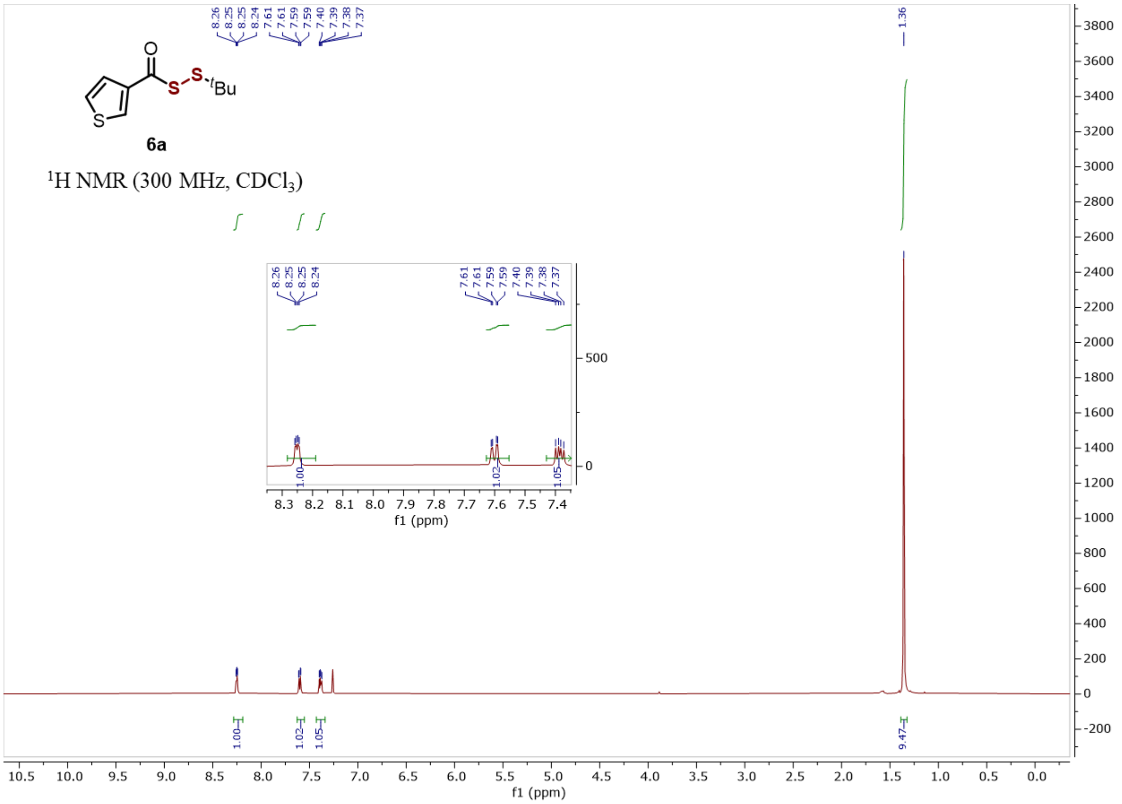

Supplementary Figure 76: ^{1}H NMR (300 MHz, $CDCl_3$) spectrum of compound **6a**.

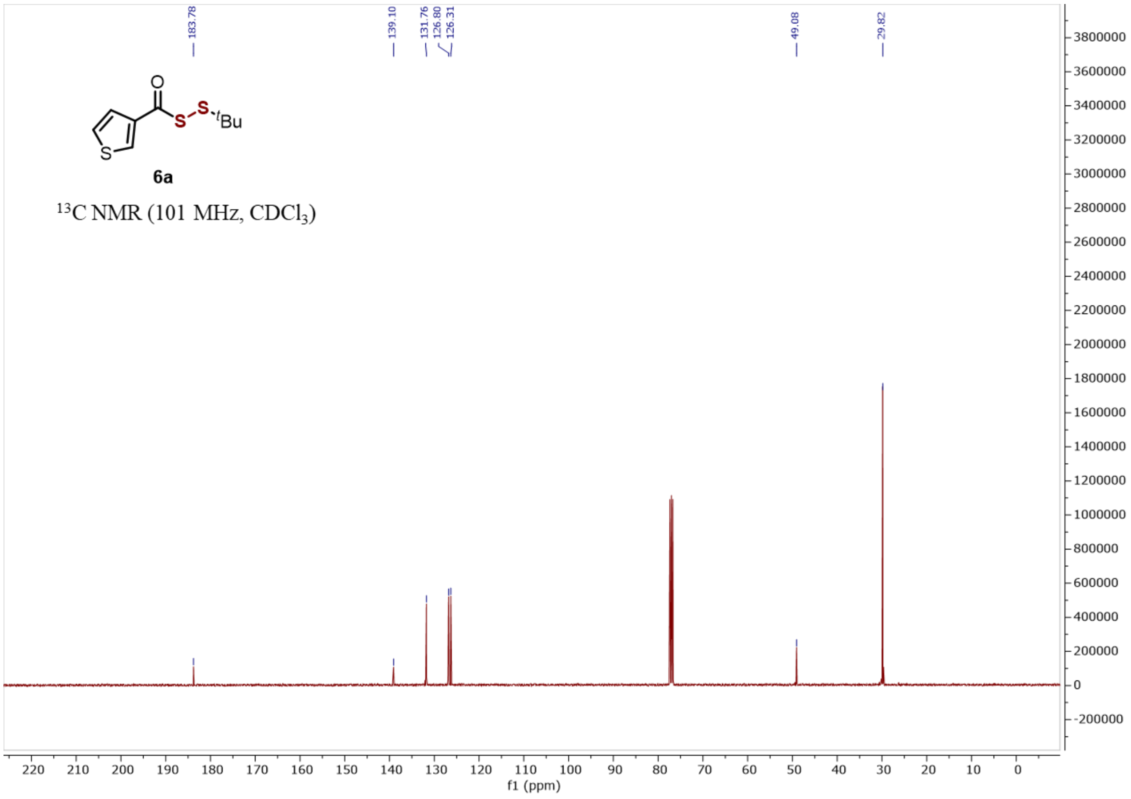

Supplementary Figure 77: ^{13}C NMR (101 MHz, $CDCl_3$) spectrum of compound **6a**.

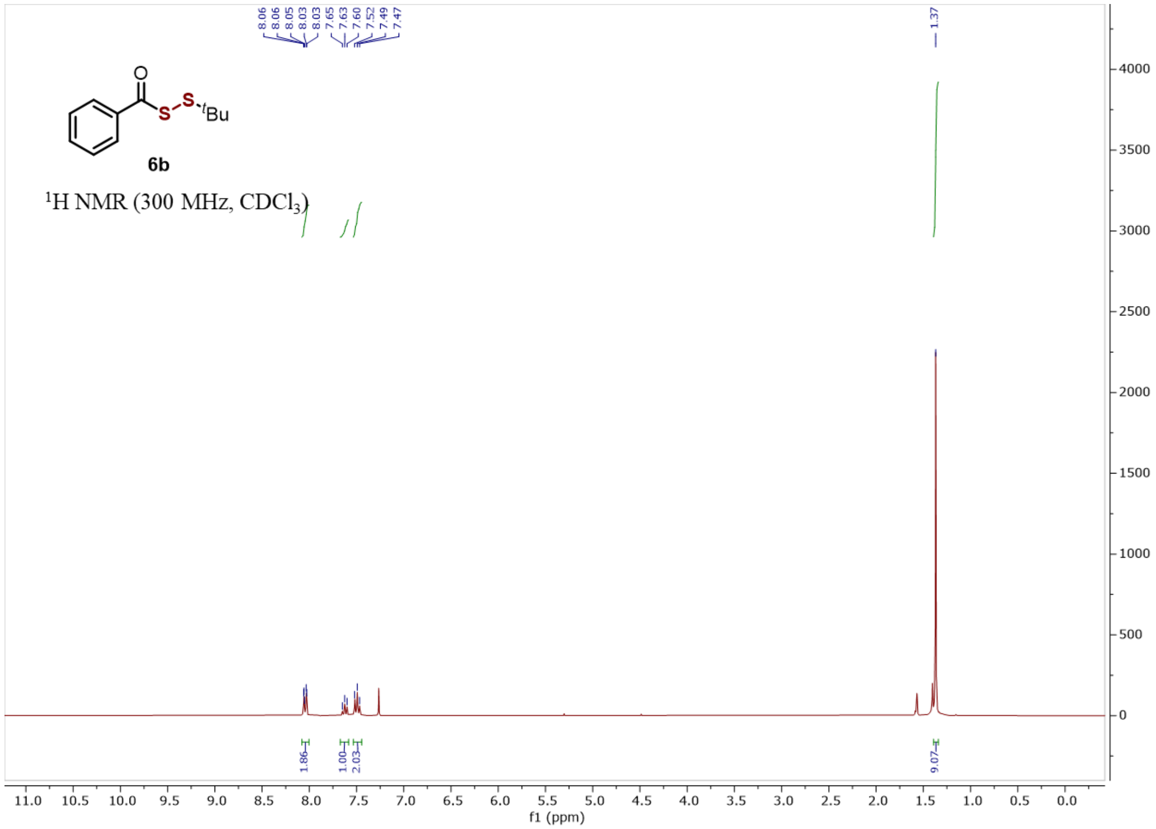

Supplementary Figure 78: ^{1}H NMR (300 MHz, $CDCl_3$) spectrum of compound **6b**.

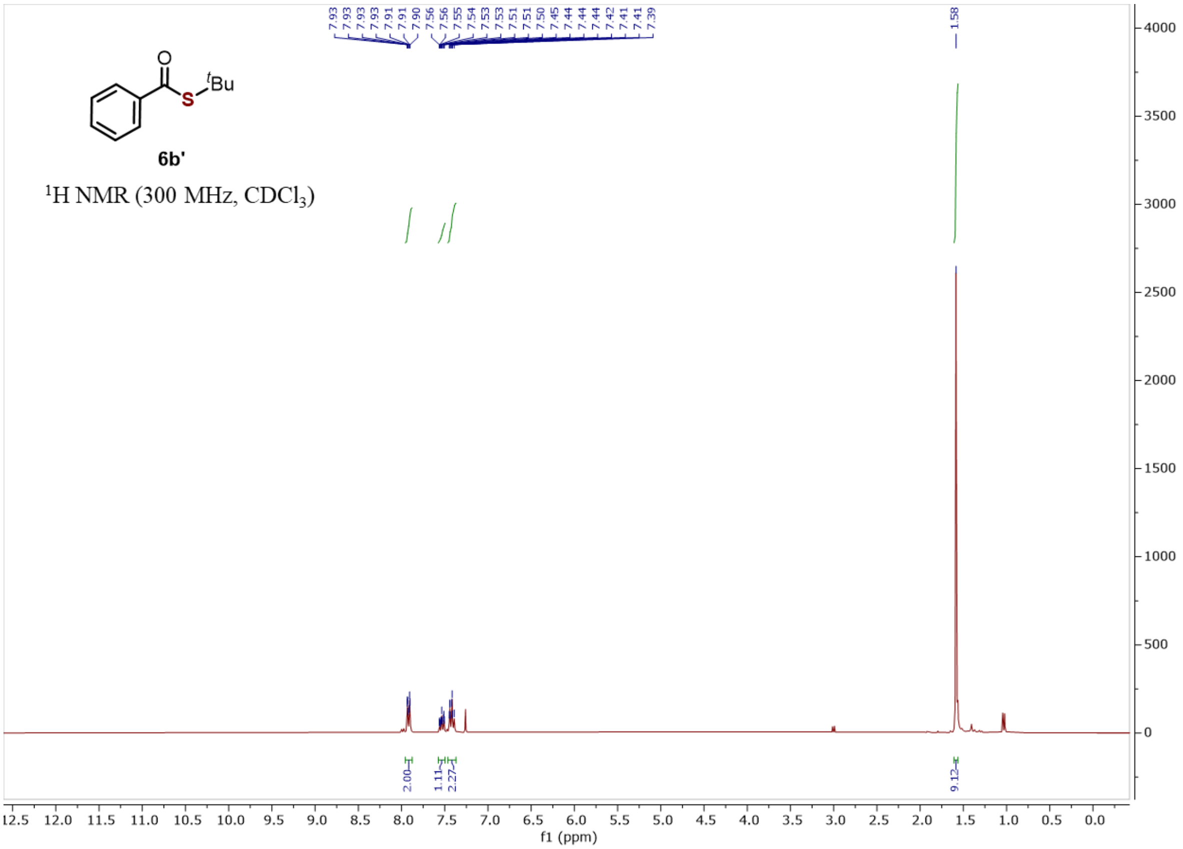

Supplementary Figure 79: ^{1}H NMR (300 MHz, CDCl$_3$) spectrum of compound **6b'**.

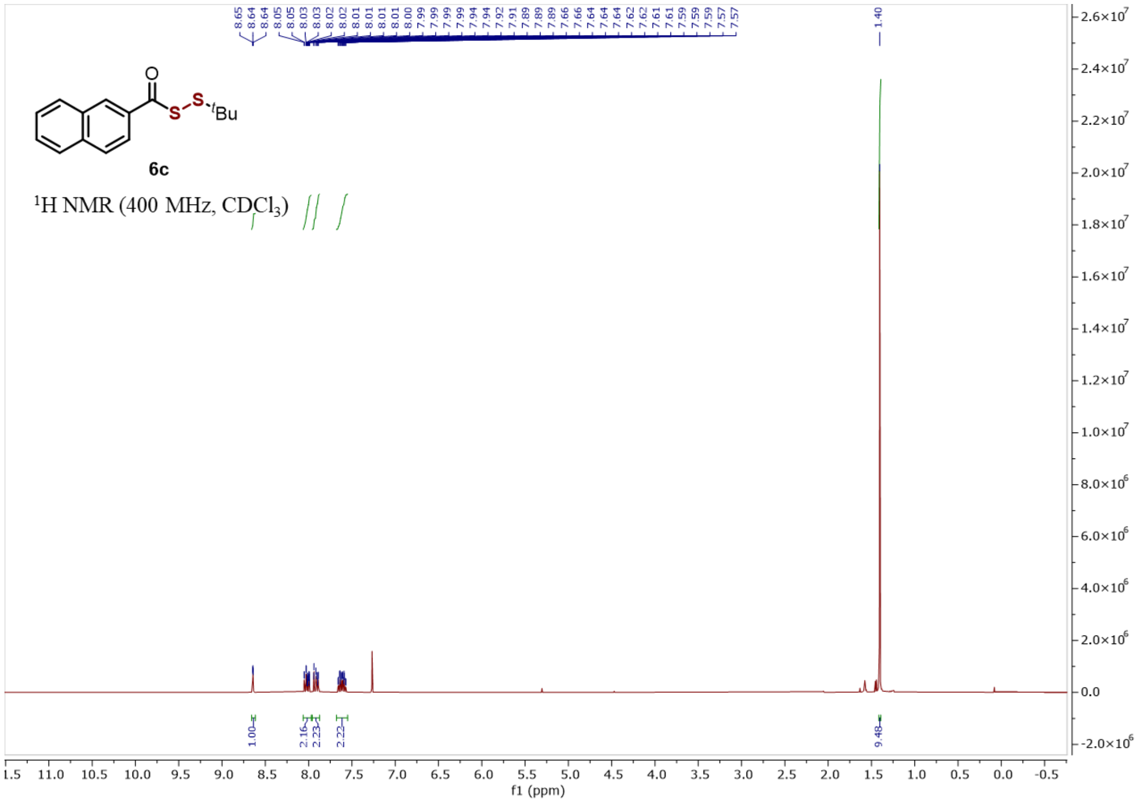

Supplementary Figure 80: ^{1}H NMR (400 MHz, $CDCl_3$) spectrum of compound **6c**.

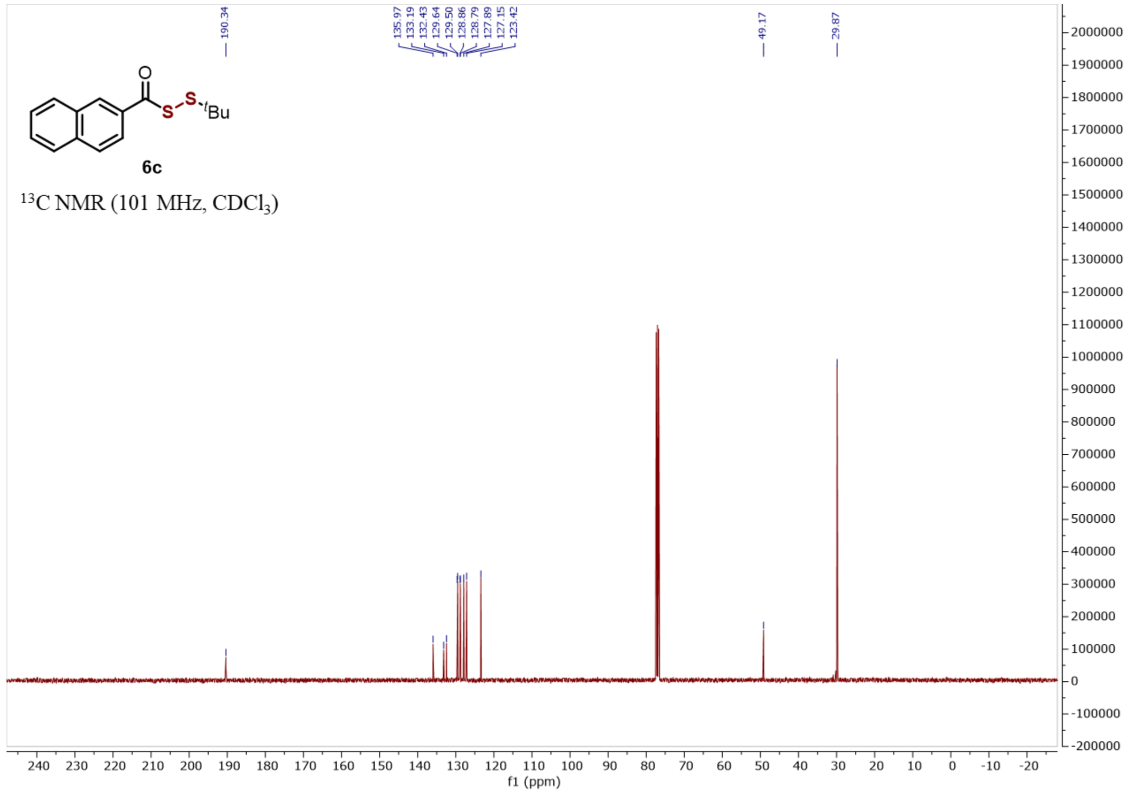

Supplementary Figure 81: ^{13}C NMR (101 MHz, $CDCl_3$) spectrum of compound **6c**.

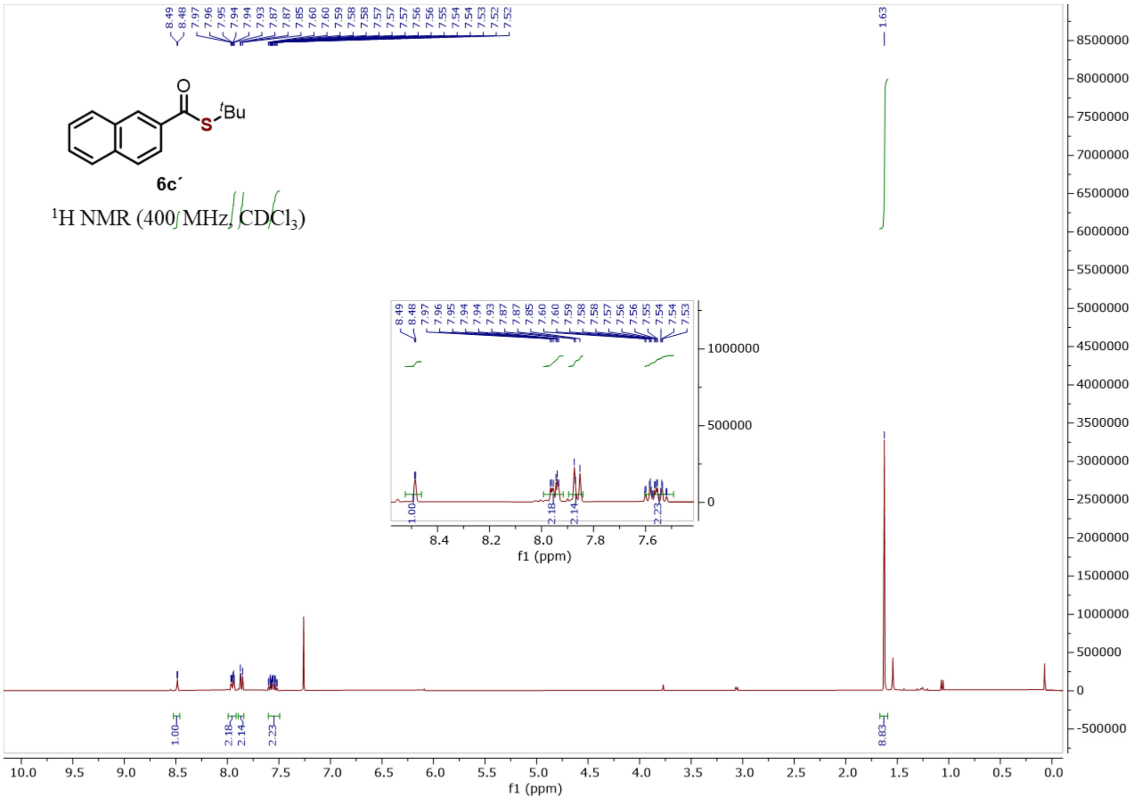

Supplementary Figure 82: ^{1}H NMR (400 MHz, $CDCl_3$) spectrum of compound **6c'**.

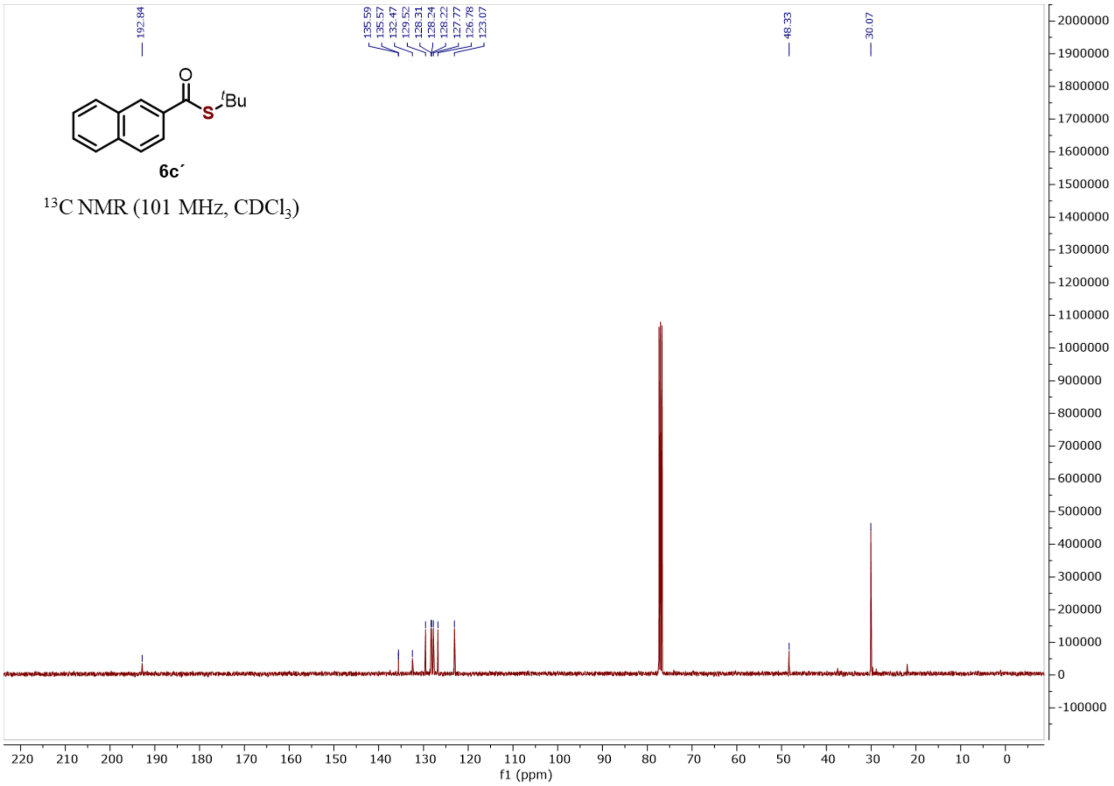

Supplementary Figure 83: ^{13}C NMR (101 MHz, $CDCl_3$) spectrum of compound **6c'**.

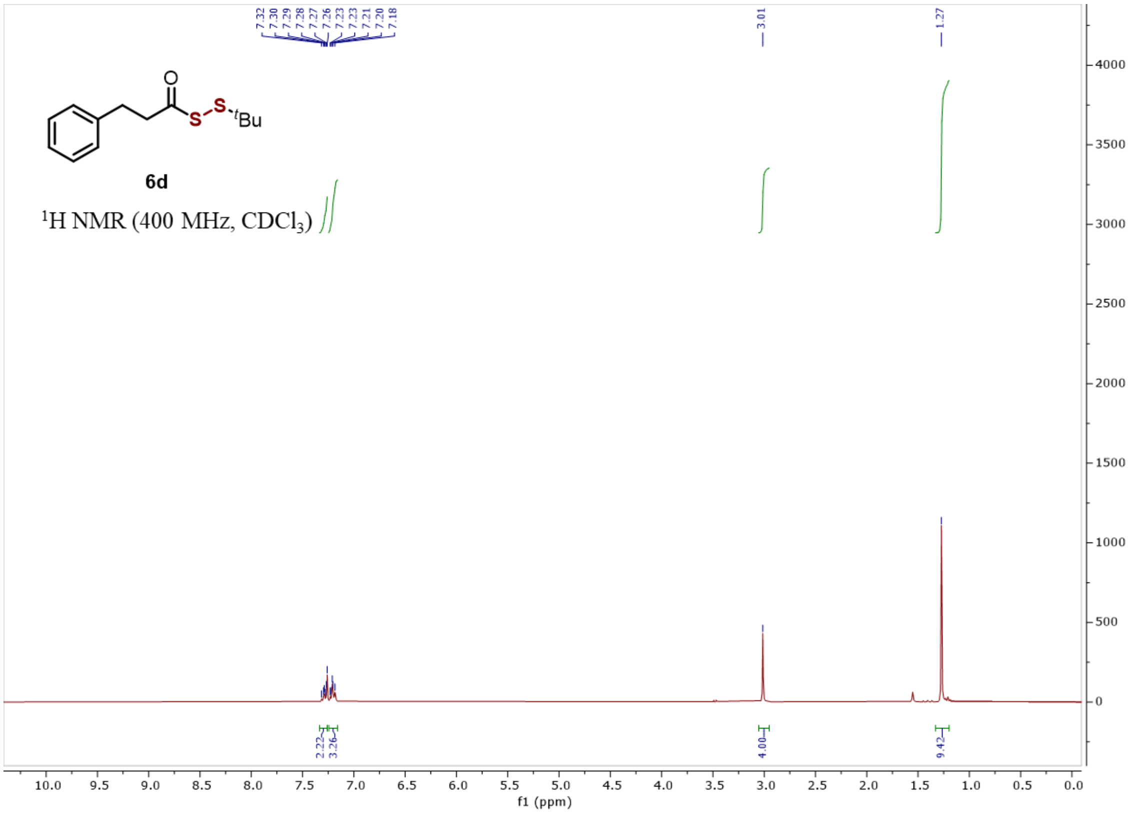

Supplementary Figure 84: ^{1}H NMR (400 MHz, $CDCl_3$) spectrum of compound **6d**.

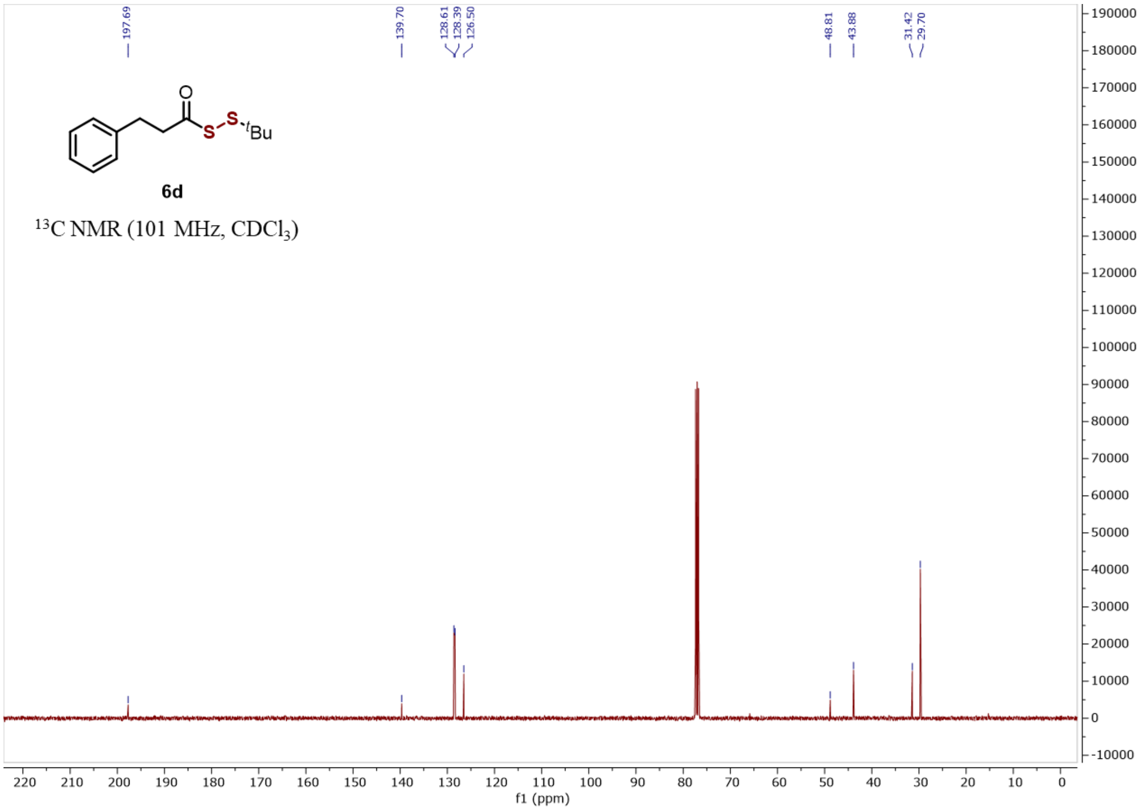

Supplementary Figure 85: ^{13}C NMR (101 MHz, $CDCl_3$) spectrum of compound **6d**.

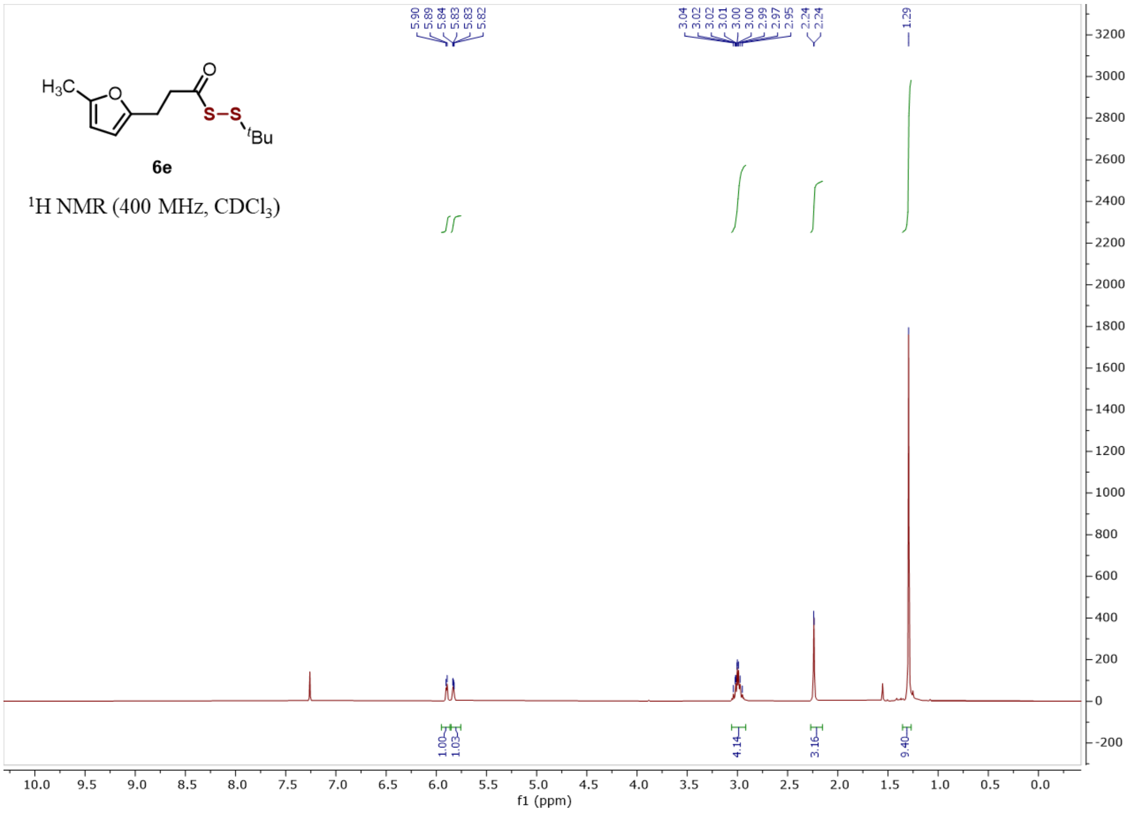

Supplementary Figure 86: ^{1}H NMR (400 MHz, $CDCl_3$) spectrum of compound **6e**.

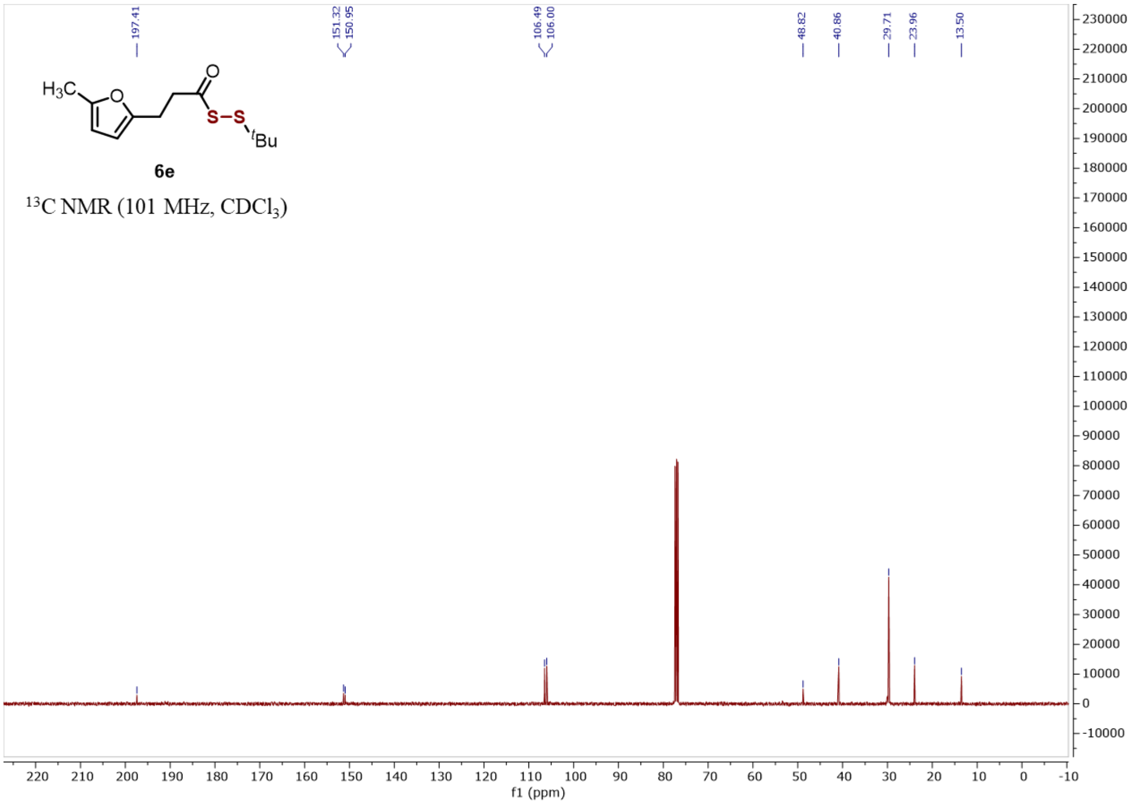

Supplementary Figure 87: ^{13}C NMR (101 MHz, $CDCl_3$) spectrum of compound **6e**.

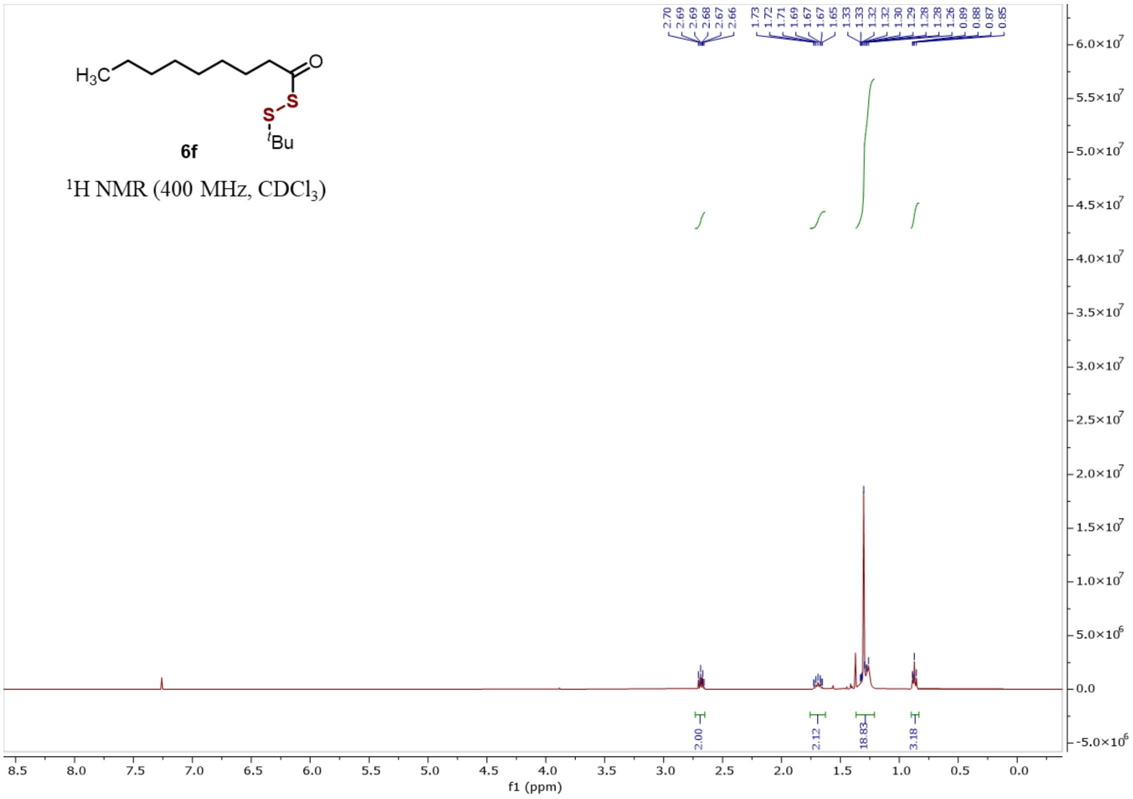

Supplementary Figure 88: ^{1}H NMR (400 MHz, $CDCl_3$) spectrum of compound **6f**.

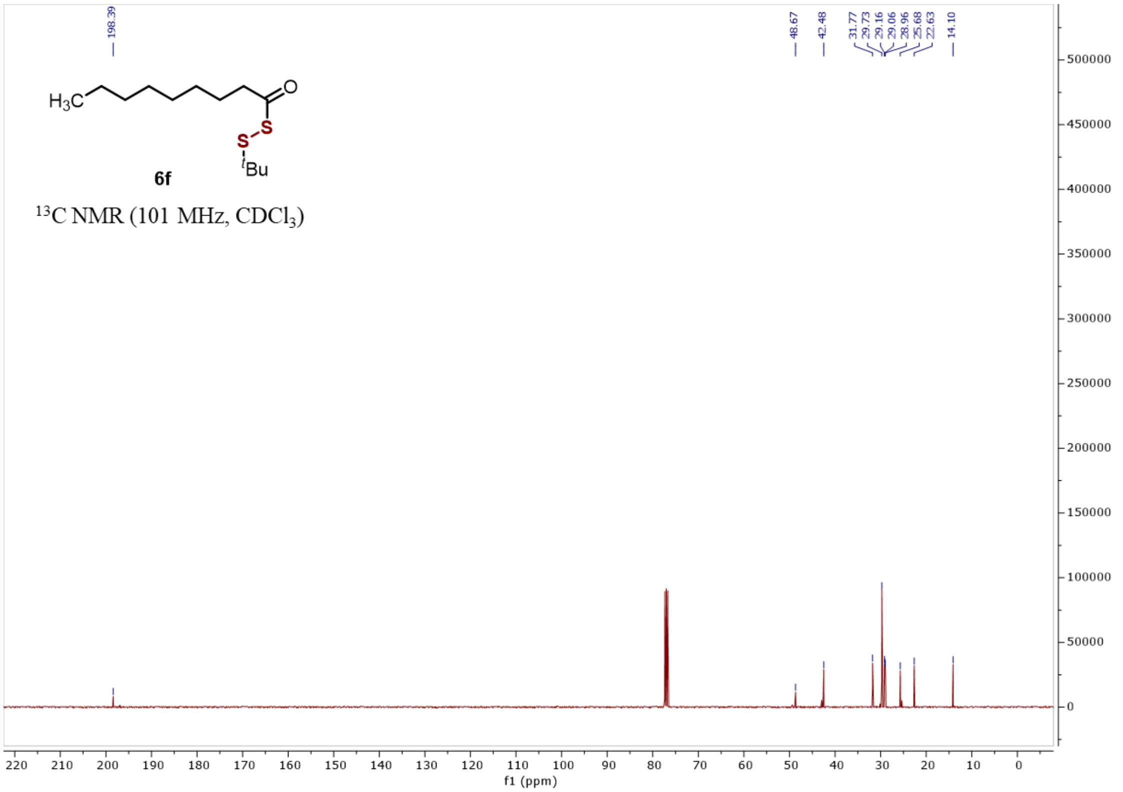

Supplementary Figure 89: ^{13}C NMR (101 MHz, $CDCl_3$) spectrum of compound **6f**.

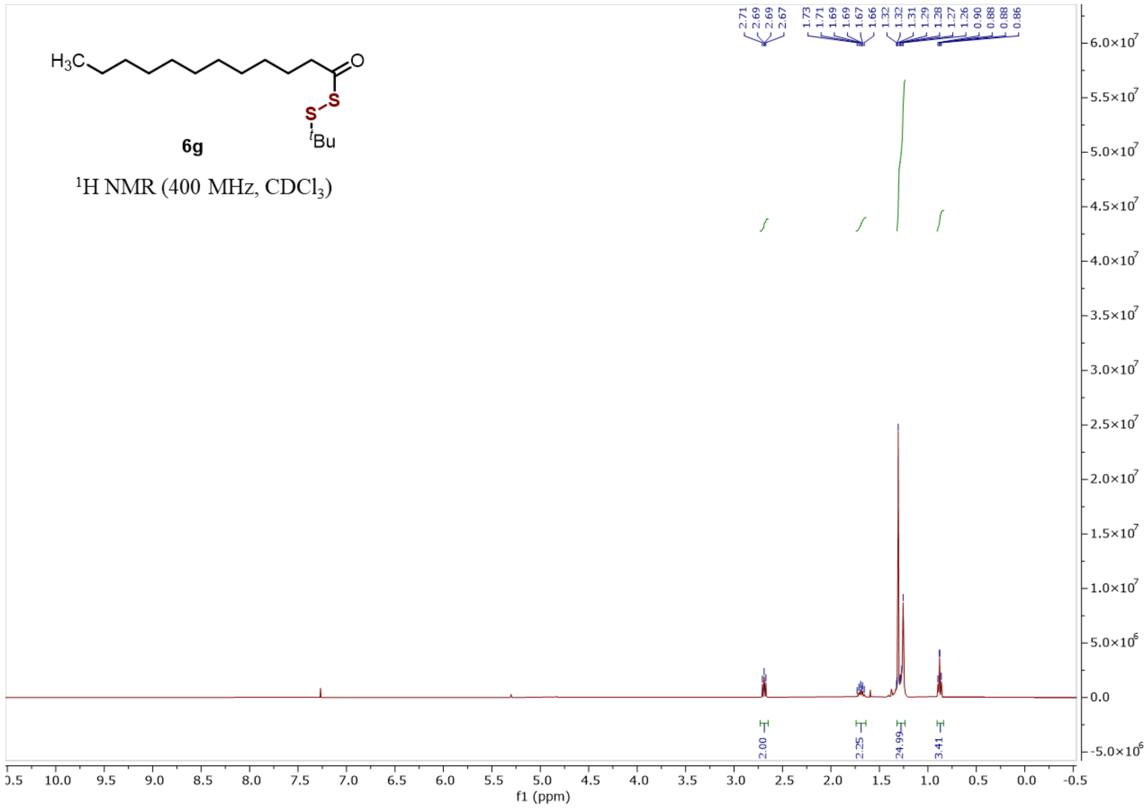

Supplementary Figure 90: ^{1}H NMR (400 MHz, $CDCl_3$) spectrum of compound **6g**.

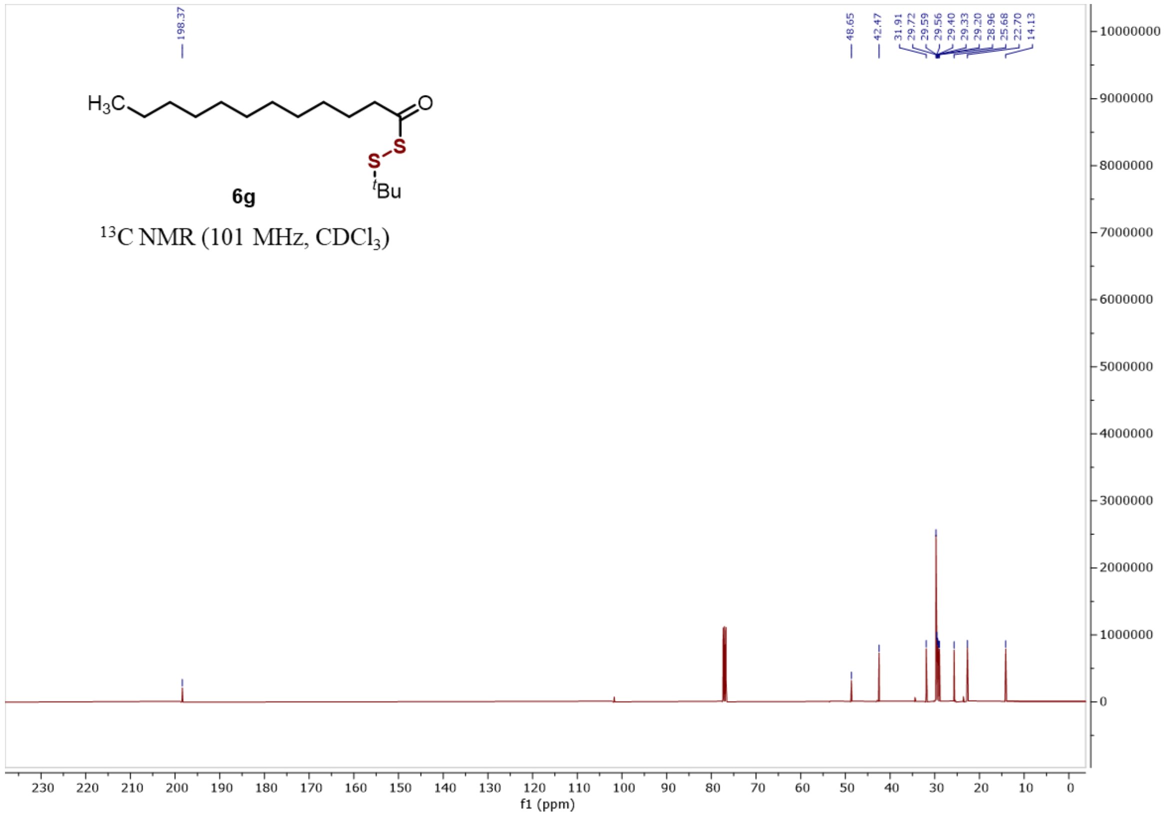

Supplementary Figure 91: ^{13}C NMR (101 MHz, $CDCl_3$) spectrum of compound **6g**.

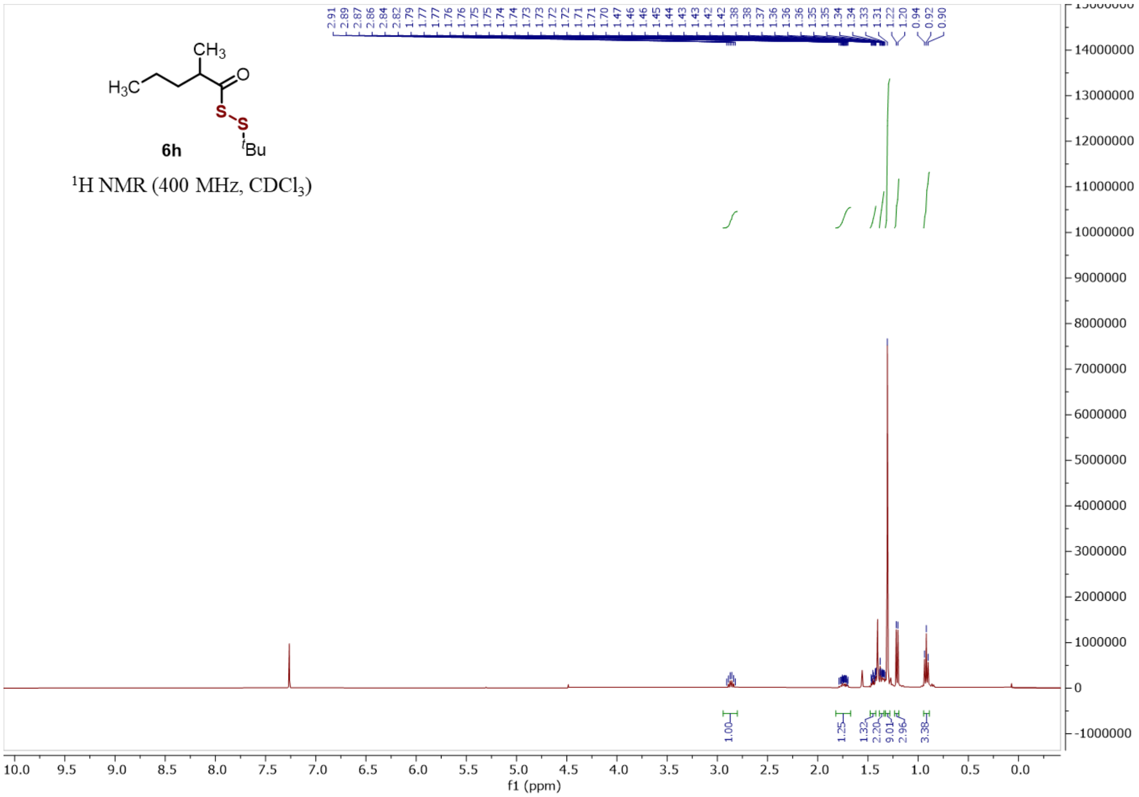

Supplementary Figure 92: ^{1}H NMR (400 MHz, $CDCl_3$) spectrum of compound **6h**.

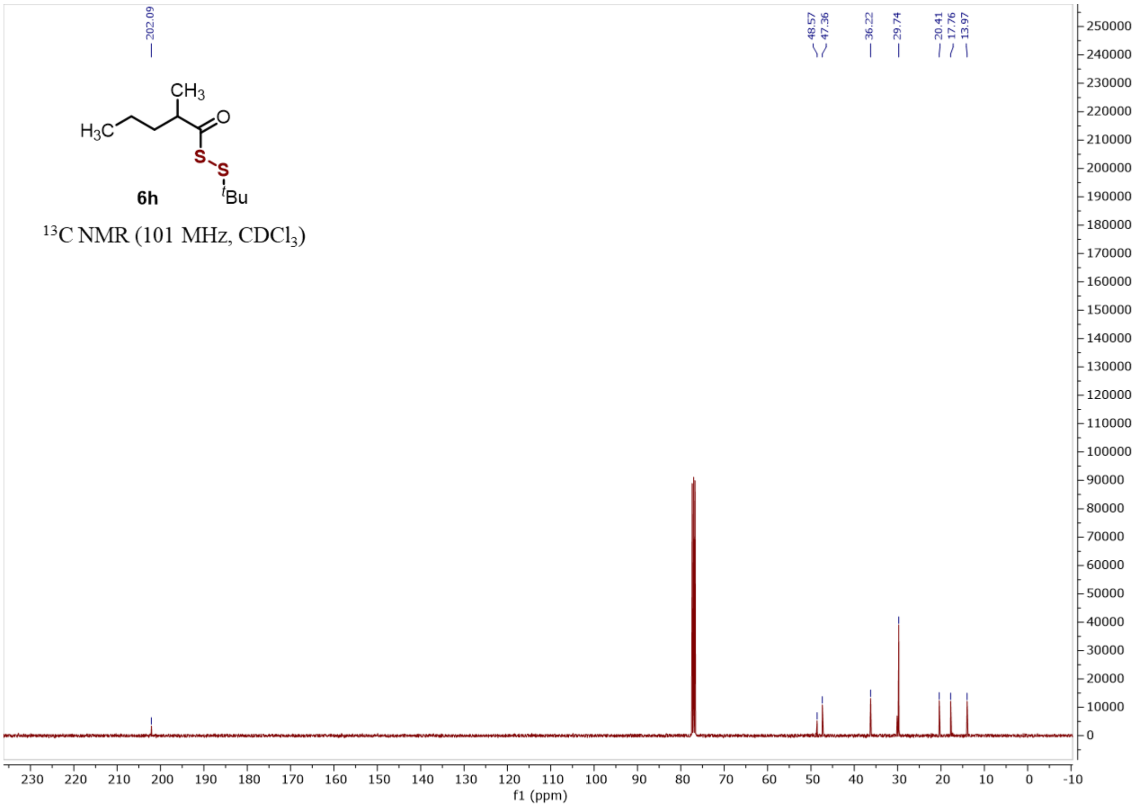

Supplementary Figure 93: ^{13}C NMR (101 MHz, $CDCl_3$) spectrum of compound **6h**.

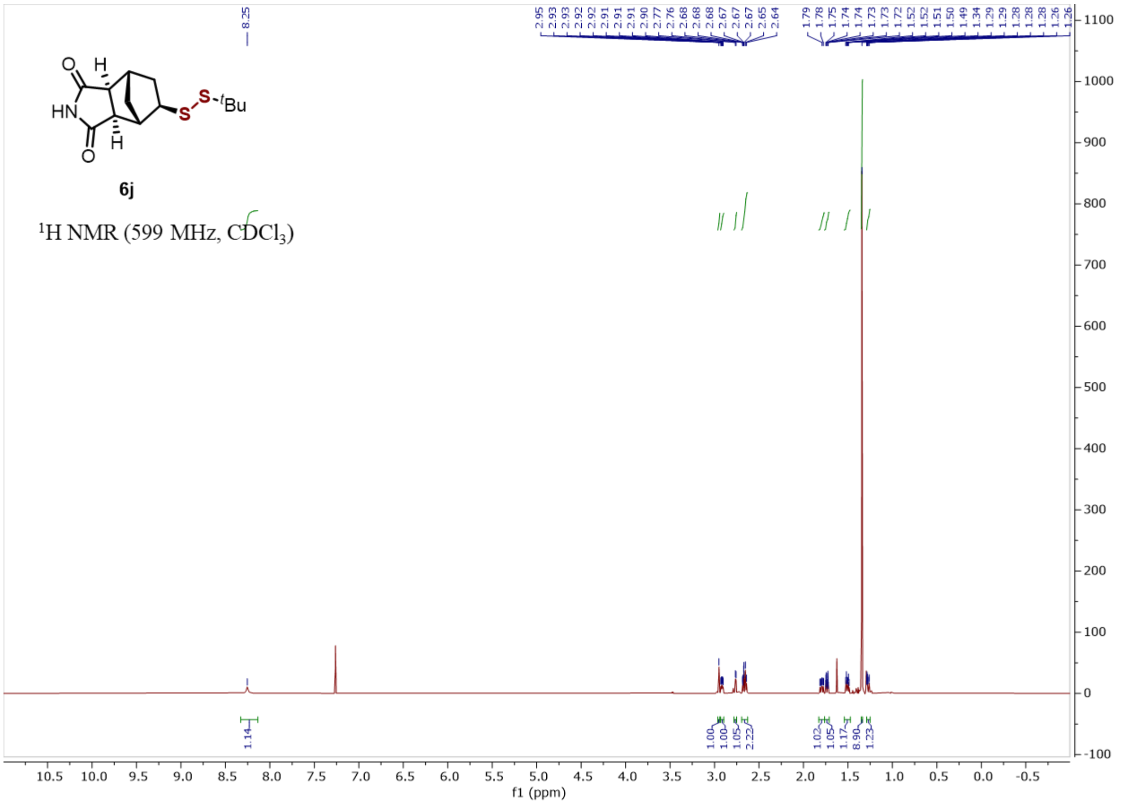

Supplementary Figure 94: ^{1}H NMR (599 MHz, $CDCl_3$) spectrum of compound **6j**.

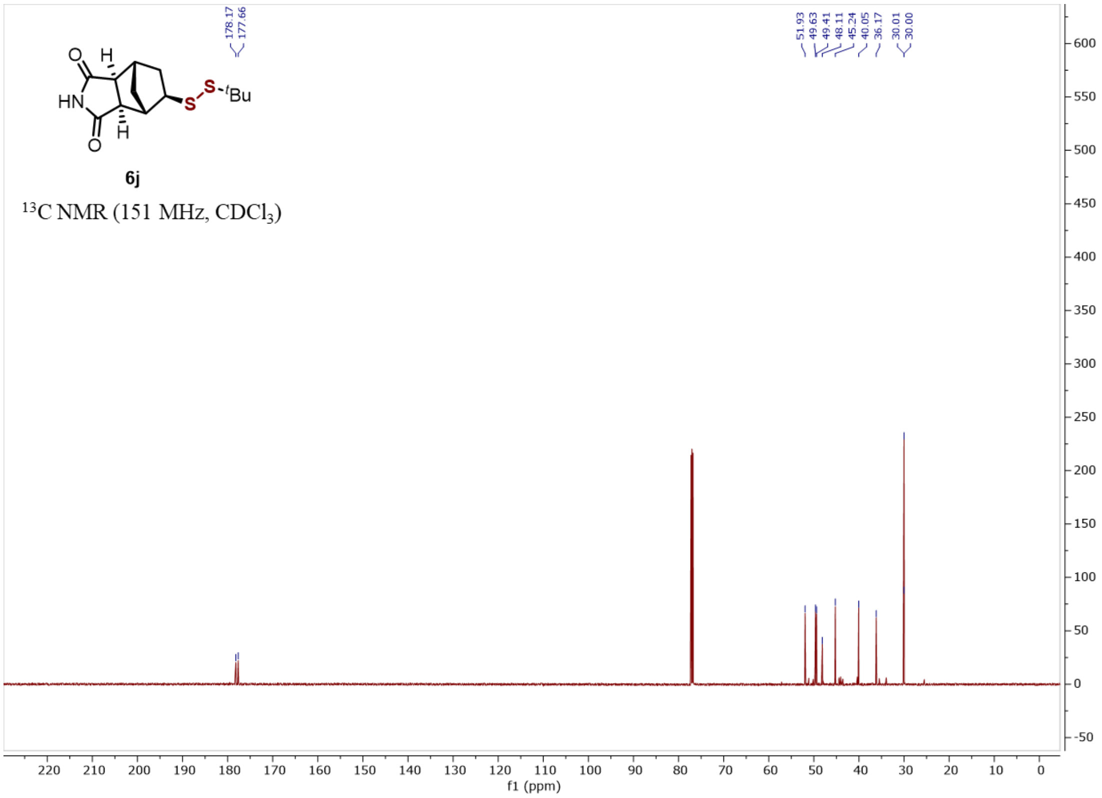

Supplementary Figure 95: ^{13}C NMR (151 MHz, $CDCl_3$) spectrum of compound **6j**.

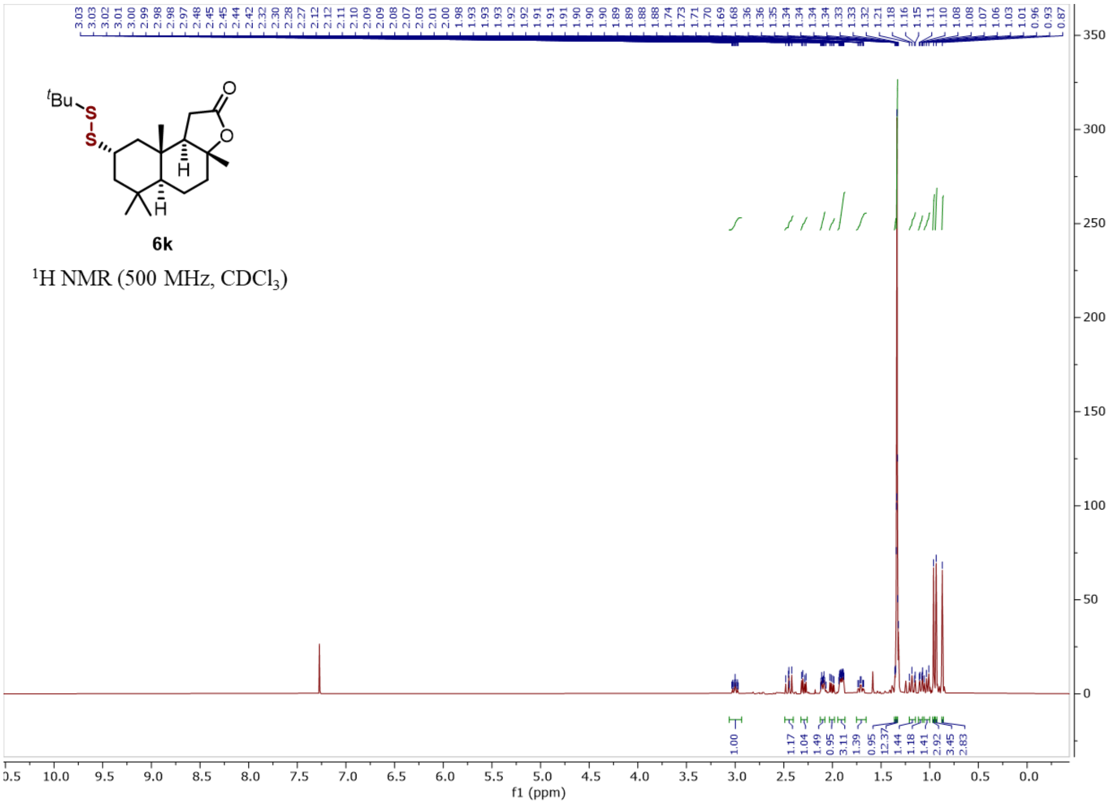

Supplementary Figure 96: ^{1}H NMR (500 MHz, $CDCl_3$) spectrum of compound **6k**.

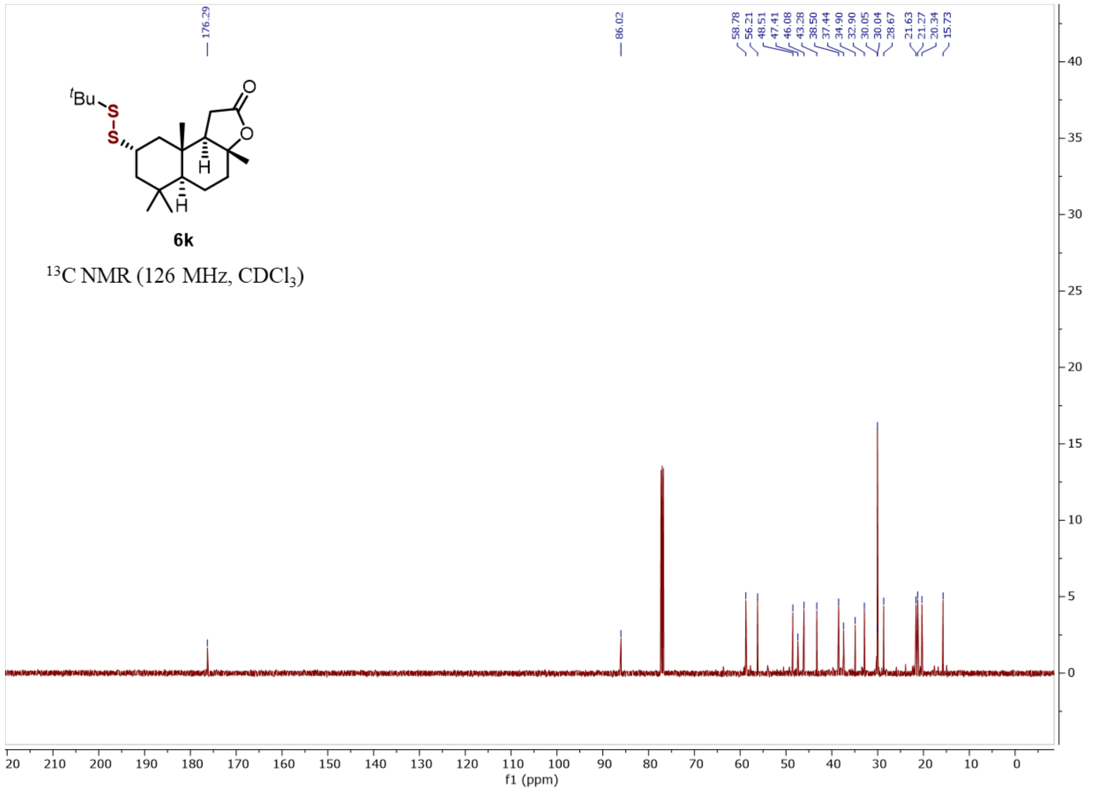

Supplementary Figure 97: ^{13}C NMR (126 MHz, $CDCl_3$) spectrum of compound **6k**.

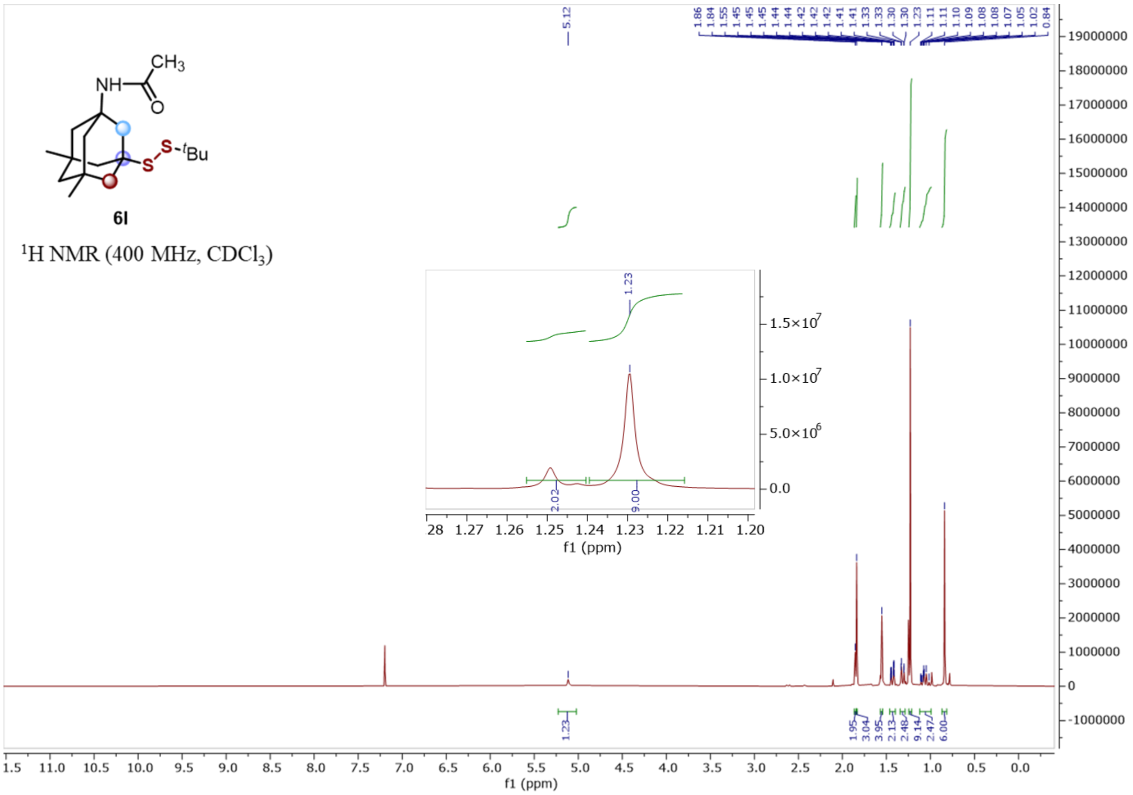

Supplementary Figure 98: ^{1}H NMR (400 MHz, $CDCl_3$) spectrum of compound **6l**.

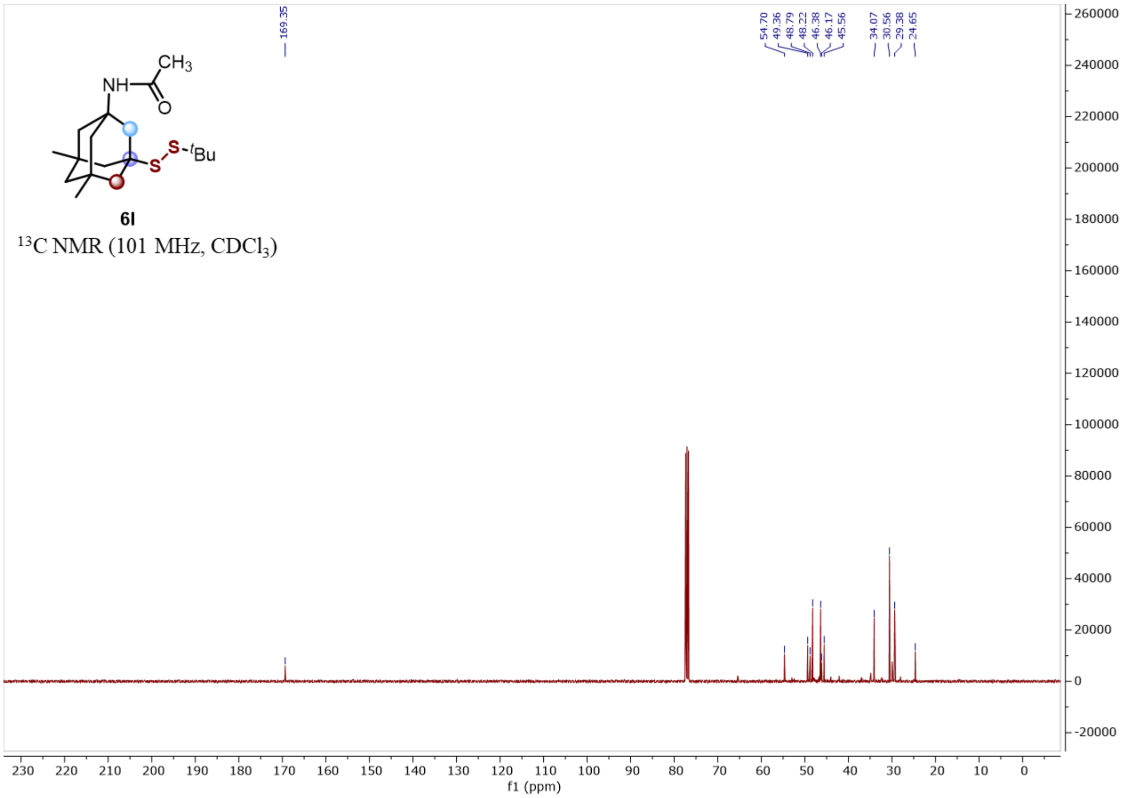

Supplementary Figure 99: ^{13}C NMR (101 MHz, $CDCl_3$) spectrum of compound **6l**.

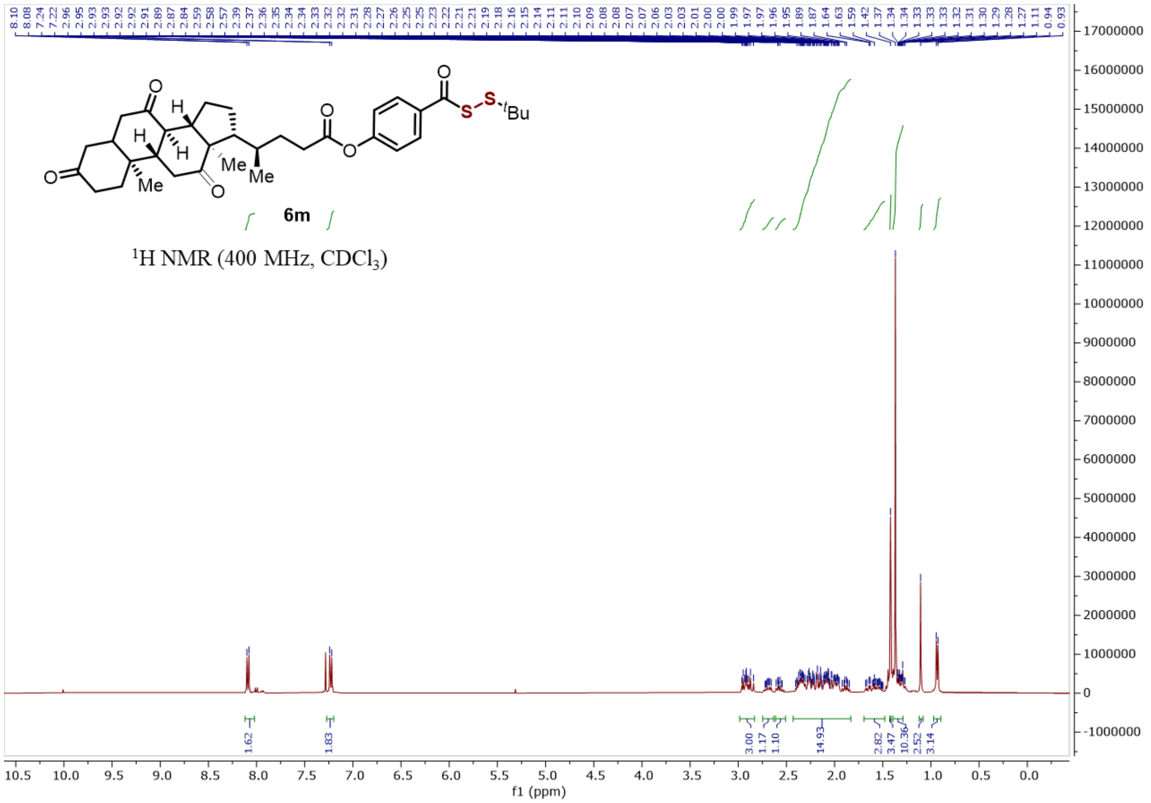

Supplementary Figure 100: ^{1}H NMR (400 MHz, CDCl$_3$) spectrum of compound **6m**.

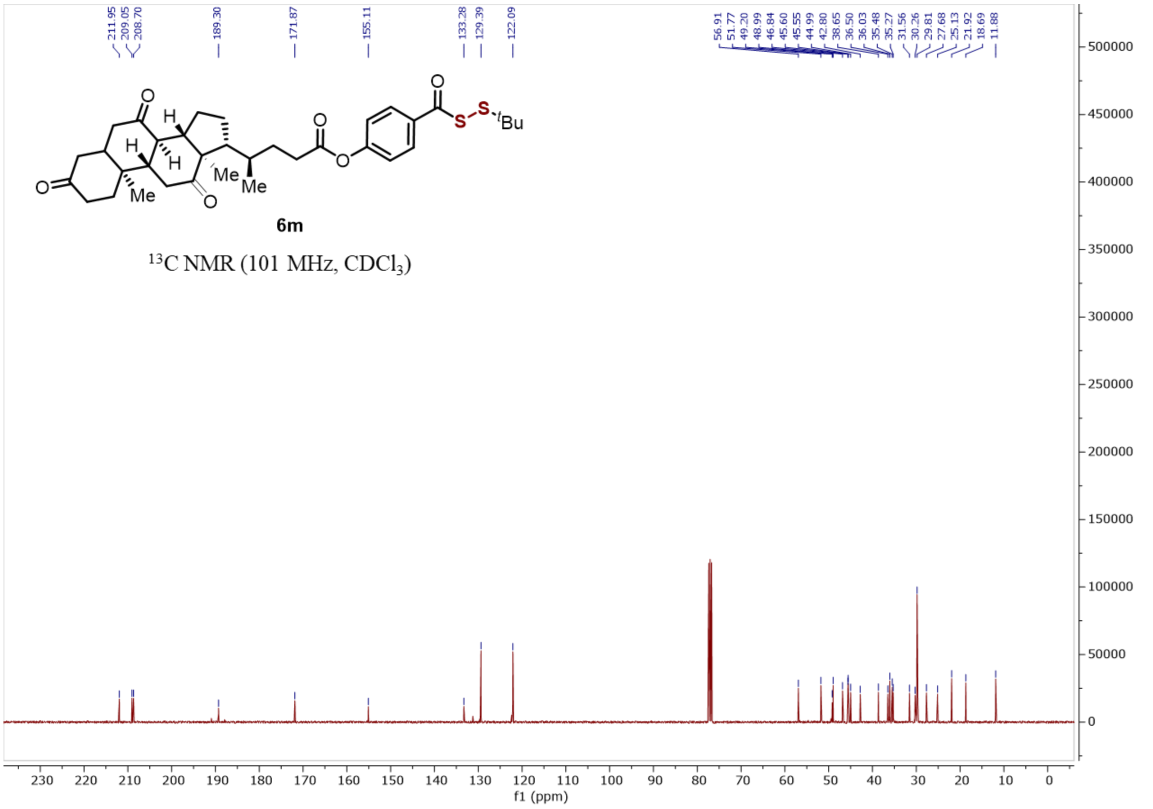

Supplementary Figure 101: ^{13}C NMR (101 MHz, CDCl$_3$) spectrum of compound **6m**.

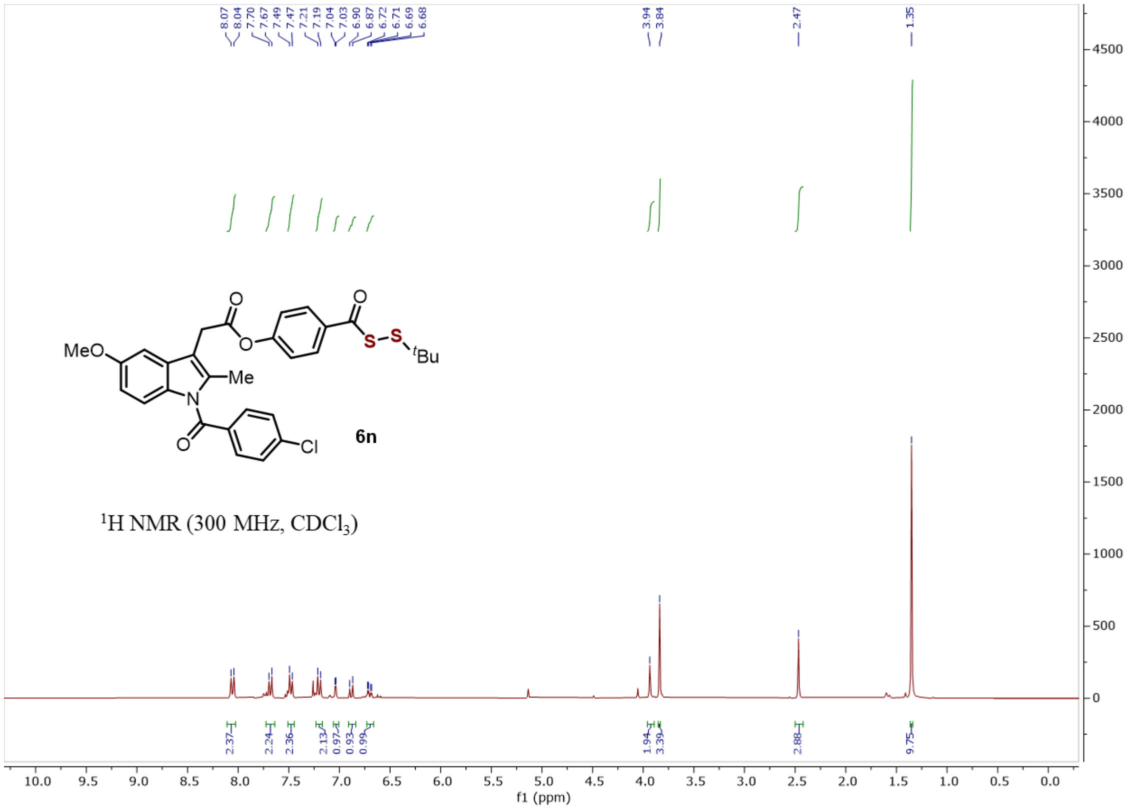

Supplementary Figure 102: ^{1}H NMR (300 MHz, CDCl$_3$) spectrum of compound **6n**.

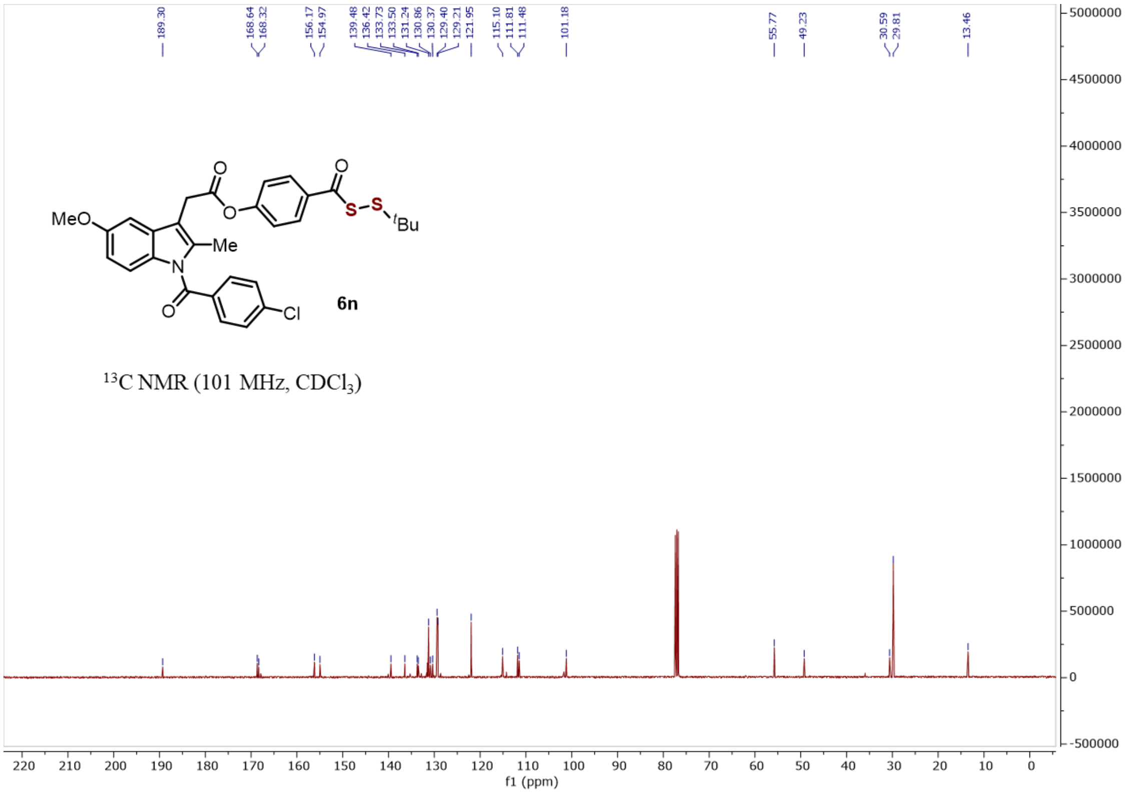

Supplementary Figure 103: ^{13}C NMR (101 MHz, CDCl$_3$) spectrum of compound **6n**.

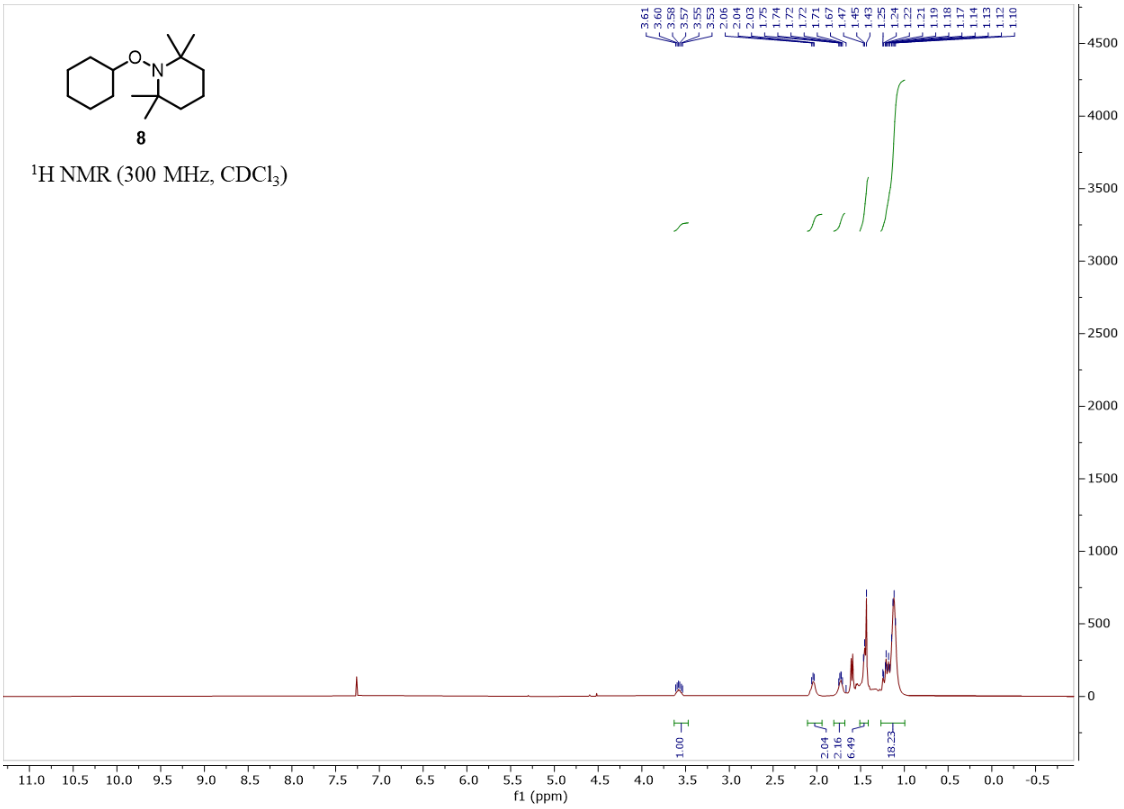

Supplementary Figure 104: ^{1}H NMR (300 MHz, $CDCl_3$) spectrum of compound **8**.

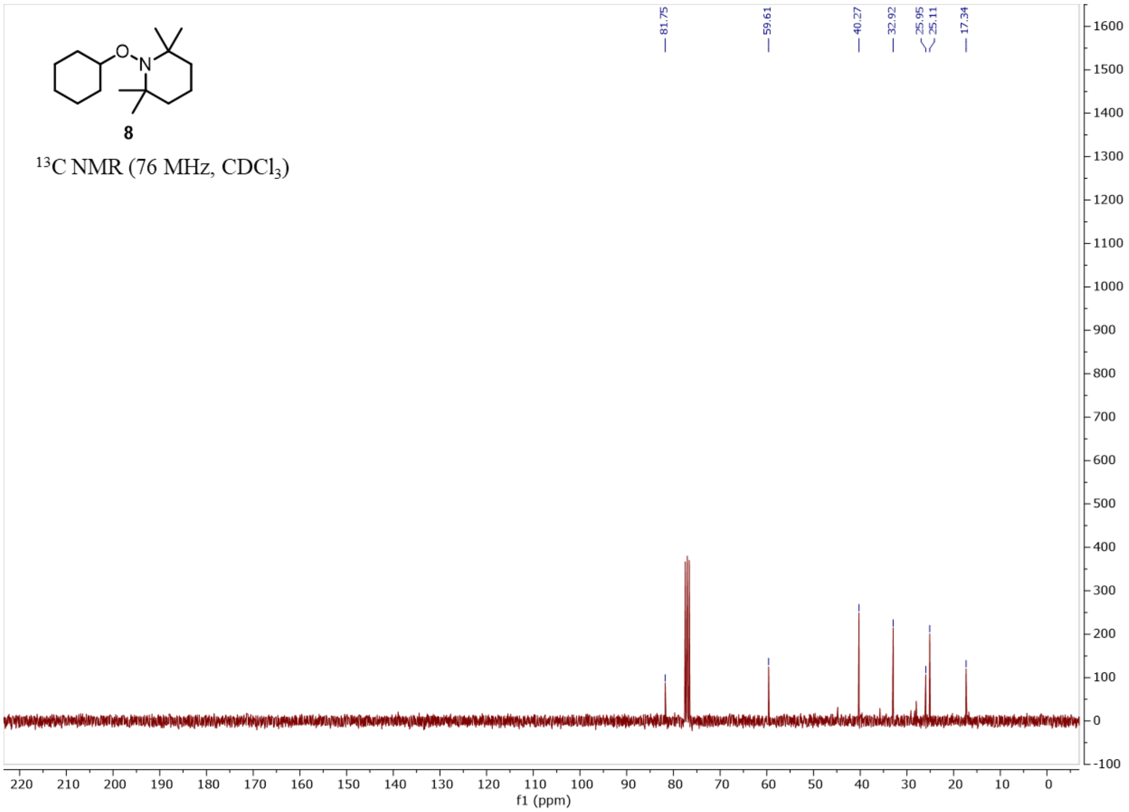

Supplementary Figure 105: ^{1}H NMR (300 MHz, $CDCl_3$) spectrum of compound **8**.

3. Supplementary References

1. Wu, Z. & Pratt, D. A. Radical Substitution Provides a Unique Route to Disulfides. *J. Am. Chem. Soc.* **142**, 10284-10290 (2020).

2. Chen, S., *et al.* Sandmeyer-Type Reductive Disulfuration of Anilines. *Org. Lett.* **23**, 7428-7433 (2021).

3. Bolton, S. G., Cerda, M. M., Gilbert, A. K. & Pluth, M. D. Effects of Sulfane Sulfur Content in Benzyl Polysulfides on Thiol-triggered H_2S Release and Cell Proliferation. *Free Radical Biol. Med.* **131**, 393-398 (2019).

4. Zysman-Colman, E. & Harpp, D. N. Optimization of the Synthesis of Symmetric Aromatic Tri- and Tetrasulfides. *J. Org. Chem.* **68**, 2487-2489 (2003).

5. Rao, M. V., Reese, C. B. & Zhao, Z. Dibenzoyl Tetrasulphide - A Rapid Sulphur Transfer Agent in the Synthesis of Phosphorothioate Analogues of Oligonucleotides. *Tetrahedron Lett.* **33**, 4839-4842 (1992).

6. Cerda, M. M., Hammers, M. D., Earp, M. S., Zakharov, L. N. & Pluth, M. D. Applications of Synthetic Organic Tetrasulfides as H_2S Donors. *Org. Lett.* **19**, 2314-2317 (2017).

7. Gong, K., Zhou, Y. & Jiang, X. From Symmetrical Tetrasulfides to Trisulfide Dioxides via Photocatalysis. *Green Chem.* **23**, 9865-9869 (2021).

8. Schwarz, J. L., Huang, H.-M., Paulisch, T. O. & Glorius, F. Dialkylation of 1,3-Dienes by Dual Photoredox and Chromium Catalysis. *ACS Catal.* **10**, 1621-1627 (2020).

9. Rostoll-Berenguer, J., Blay, G., Pedro, J. R. & Vila, C. Photocatalytic Giese Addition of 1,4-Dihydroquinoxalin-2-ones to Electron-Poor Alkenes Using Visible Light. *Org. Lett.* **22**, 8012-8017 (2020).

10. de Pedro Beato, E., Spinnato, D., Zhou, W. & Melchiorre, P. A General Organocatalytic System for Electron Donor–Acceptor Complex Photoactivation and Its Use in Radical Processes. *J. Am. Chem. Soc.* **143**, 12304-12314 (2021).

11. Wan, T., *et al.* Decatungstate-Mediated C(sp^3)–H Heteroarylation via Radical-Polar Crossover in Batch and Flow. *Angew. Chem. Int. Ed.* **60**, 17893-17897 (2021).

12. Duncan, D. C. & Fox, M. A. Early Events in Decatungstate Photocatalyzed Oxidations: A Nanosecond Laser Transient Absorbance Reinvestigation. *J. Phys. Chem. A* **102**, 4559-4567 (1998).

13. Bao, Y., Mo, X., Xu, X., He, Y., Xu, X. & An, H. Stability Studies of Anticancer Agent Bis(4-fluorobenzyl)trisulfide and Synthesis of Related Substances. *J. Pharm. Biomed. Anal.* **48**, 664-671 (2008).

14. Abu-Yousef, I. A., Rys, A. Z. & Harpp, D. N. Preparation and Reactivity of Unsymmetrical Di- and Trisulfides. *J. Sulfur Chem.* **27**, 15-24 (2006).

15. Bołt, M. & Żak, P. Application of Bulky NHC–Rhodium Complexes in Efficient S–Si and S–S Bond Forming Reactions. *Inorg. Chem.* **60**, 17579-17585 (2021).

16. Qiu, X., Yang, X., Zhang, Y., Song, S. & Jiao, N. Efficient and Practical Synthesis of Unsymmetrical Disulfides via Base-catalyzed Aerobic Oxidative Dehydrogenative Coupling of Thiols. *Org. Chem. Front.* **6**, 2220-2225 (2019).

17. Martín, M. T., Marín, M., Maya, C., Prieto, A. & Nicasio, M. C. Ni(II) Precatalysts Enable Thioetherification of (Hetero)Aryl Halides and Tosylates and Tandem C−S/C−N Couplings. *Chem. Eur. J.* **27**, 12320-12326 (2021).

18. Tsai, C.-H., *et al.* PIFA-Mediated Synthesis of Acylsulfenic Acid Alkyl Esters and Benzoyl Alkyl Disulfides from Thioacids. *Synthesis* **48**, 4459-4464 (2016).

19. Xu, T., Cao, T., Yang, M., Xu, R., Nie, X. & Liao, S. Decarboxylative Thiolation of Redox-Active Esters to Thioesters by Merging Photoredox and Copper Catalysis. *Org. Lett.* **22**, 3692-3696 (2020).

20. Boeini, H. Z. & Mobin, M. One-Step Conversion of Alcohols into Thioesters. *Synlett* **2010**, 2861-2866 (2010).
